# Predicting personalised absolute treatment effects in individual participant data meta‐analysis: An introduction to splines

**DOI:** 10.1002/jrsm.1546

**Published:** 2022-01-18

**Authors:** Michail Belias, Maroeska M. Rovers, Jeroen Hoogland, Johannes B. Reitsma, Thomas P. A. Debray, Joanna IntHout

**Affiliations:** ^1^ Health Evidence Radboud University Medical Center Nijmegen The Netherlands; ^2^ Julius Center for Health Sciences and Primary Care University Medical Center Utrecht, Utrecht University Utrecht The Netherlands; ^3^ Cochrane Netherlands University Medical Center Utrecht, Utrecht University Utrecht The Netherlands

## 1 INTRODUCTION

One of the main goals of an individual participant data meta‐analysis (IPD‐MA) of intervention studies is to investigate whether treatment effect differences are present, and how they are associated with patient characteristics.^1^ Examining treatment heterogeneity due to a continuous covariable (e.g., BMI or age) may be challenging, since there is often no prior knowledge on functional form of the conditional association between the outcome and the continuous variable.

Naïve but often‐used approaches are to ignore the possibly complex nature of the functional form and assume a linear effect, or to provide a step‐wise approximation based on categorisation of the continuous variable. Categorisation leads to loss of information, reduced power, inflation of the type I error rates, and biased results.^2^, ^3^, ^4^, ^5^ Linear modelling may also lead to biased results since the model may be too simplistic for the data. Therefore, there is a clear need for methods that can capture non‐linear relations in IPD‐MA context.

Modelling treatment effect differences whilst accounting for non‐linear functional shapes may provide the opportunity to accurately make inferences whether a patient should be treated or not. To account for non‐linearities we may estimate the functional shape of the associations and investigate potential treatment effect differences.^6^ So far, a variety of methods that account for non‐linear functional shapes has been proposed.^7^, ^8^, ^9^, ^10^, ^11^, ^12^, ^13^, ^14^, ^15^, ^16^ In this manuscript, we focus on the use of splines since they can capture both non‐linear main effects and non‐linear treatment‐covariable interaction effects without the need to pre‐specify their functional form. The class of spline methods is still broad and includes fully parametric, semi‐parametric and even non‐parametric approaches, and allows for penalisation and clustering. Thus, splines are very flexible and can address a great variety of fitting problems. We focus on four widely used types of splines; restricted cubic splines as described by Harrell,^17^ natural B‐splines,^18^, ^19^ smoothing splines^20^ and P‐splines.^21^

Splines are being used in single studies, both in intervention and prediction studies, and are also available in IPD‐MA context. In IPD‐MA, models can be estimated in either one or two stages. A generalised additive mixed effects model (GAMM)^22^ provides a one‐stage IPD‐MA method that incorporates the flexibility provided by splines. GAMMs fit a generalised additive model using covariables with or without spline transformation, while adjusting for within‐study clustering of the participants based on random effects. In two‐stage IPD‐MA, appropriate spline‐based models are fitted per study in the first stage. Subsequently, study specific estimates are extracted and pooled in the second stage. Pooling study specific predictions and their standard errors is referred to as pointwise meta‐analysis.^14^ Pooling of study‐specific model coefficients and their variance–covariance matrix is referred to as multivariate meta‐analysis.^23^

While the methodology has progressed, the use of splines in IPD‐MA is relatively uncommon. A possible explanation is that the available guidance is limited. Perperoglou et al. provided a review of splines approaches, but limited to single studies.^24^ White et al.^25^ compared pointwise meta‐analysis and multivariate meta‐analysis techniques in presence of non‐linear associations, but used fractional polynomials instead of splines. Gasparrini et al.^23^ described the use of B‐splines in combination with multivariate meta‐analysis. They mentioned that multivariate meta‐analysis may be combined with other approaches to account for non‐linearities but do not provide details. Riley et al.^26^ described both multivariate meta‐analysis and one‐stage mixed effects modelling. However, most of the examples were limited to either linear associations or a combination of restricted cubic splines based on truncated power series and multivariate meta‐analysis.

Our goal is to explain and illustrate how to predict a conditional absolute treatment effect, as this measure is most relevant for clinical decision‐making.^27^ We use the four spline approaches in scenarios with multiple studies, using artificial datasets for illustration. We generated the data such that no confounder adjustment was necessary, but if needed the methods can also be implemented after individual‐level confounder adjustment. We describe the various spline approaches and their application in IPD‐MA using pointwise meta‐analysis,^14^ multivariate meta‐analysis,^23^ and GAMMs,^22^ and we provide the corresponding R‐code. We also describe the results of the aforementioned spline and pooling methods using an empirical individual participant data‐set, investigating the effect of antibiotics in children with acute otitis media (AOM).^28^

## 2 ILLUSTRATIVE EXAMPLES

**FIGURE 1:**
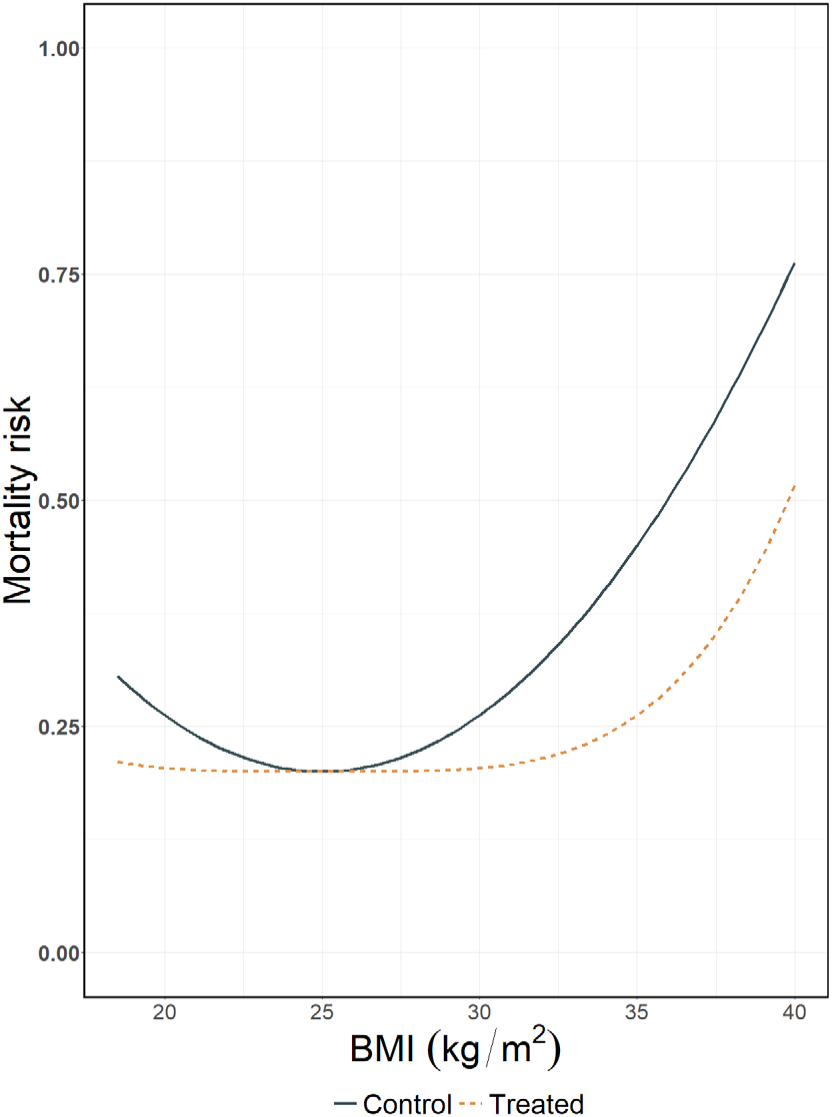
Simulated association between mortality risk and BMI in a single study [Colour figure can be viewed at wileyonlinelibrary.com]

In order to illustrate the aforementioned spline approaches in IPD‐MA we generated artificial data to mimic a previously reported non‐linear association between BMI and mortality.^29^, ^30^ We consider the case where the outcome is binary, but note that splines may be used to other types of outcomes such as continuous and time‐to‐event outcomes. For the control group we generated a (quadratic) J‐shaped association showing increased mortality for underweight and overweight participants. For the experimental group we assume a (quartic) levelled J‐shaped association, where the association between BMI and mortality is much weaker, especially for those with a < 30 BMI (Figure 1).

**FIGURE 2:**
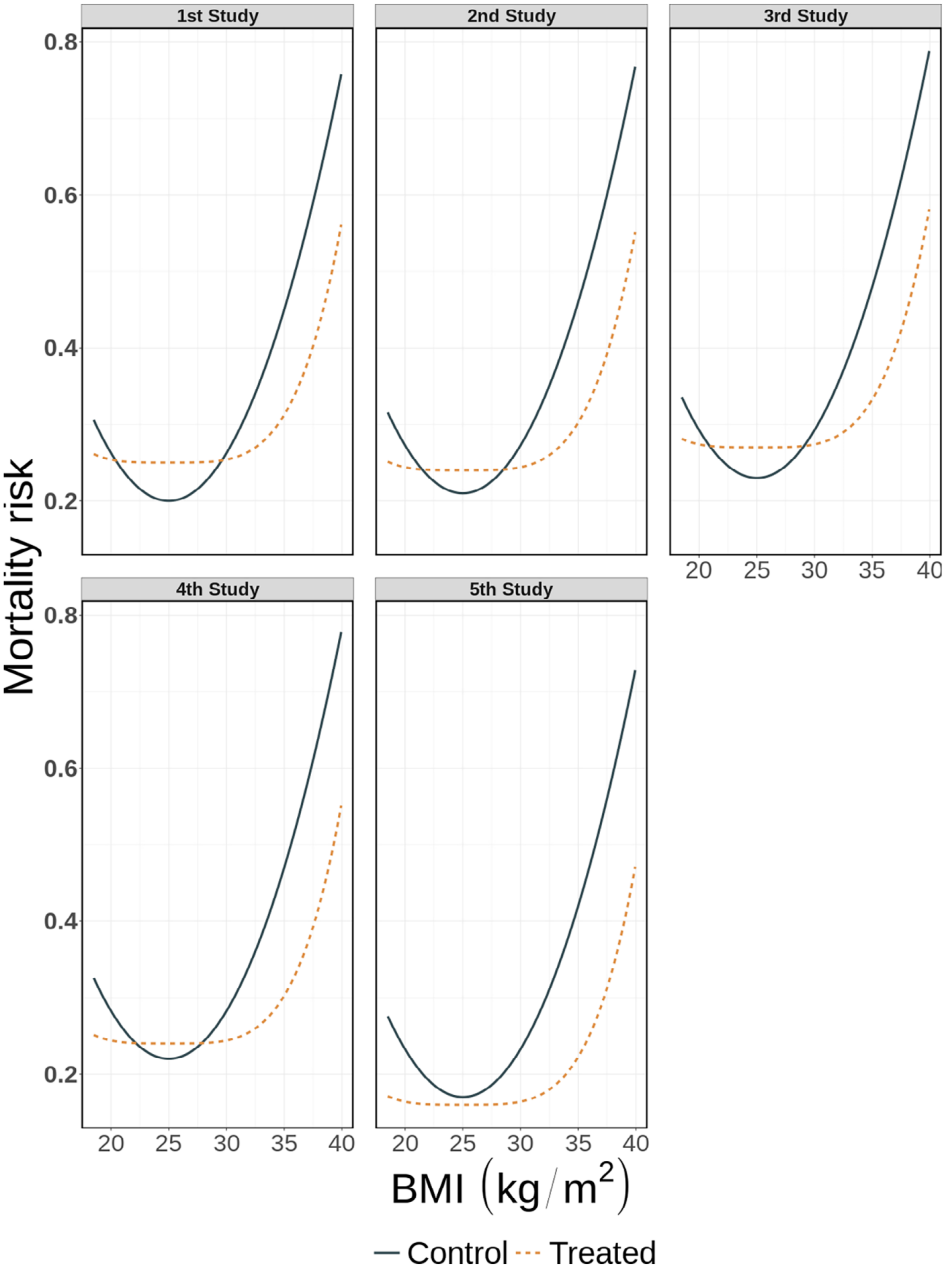
Association between mortality risk and BMI per study in the heterogeneous dataset with equal BMI ranges [Colour figure can be viewed at wileyonlinelibrary.com]

**FIGURE 3:**
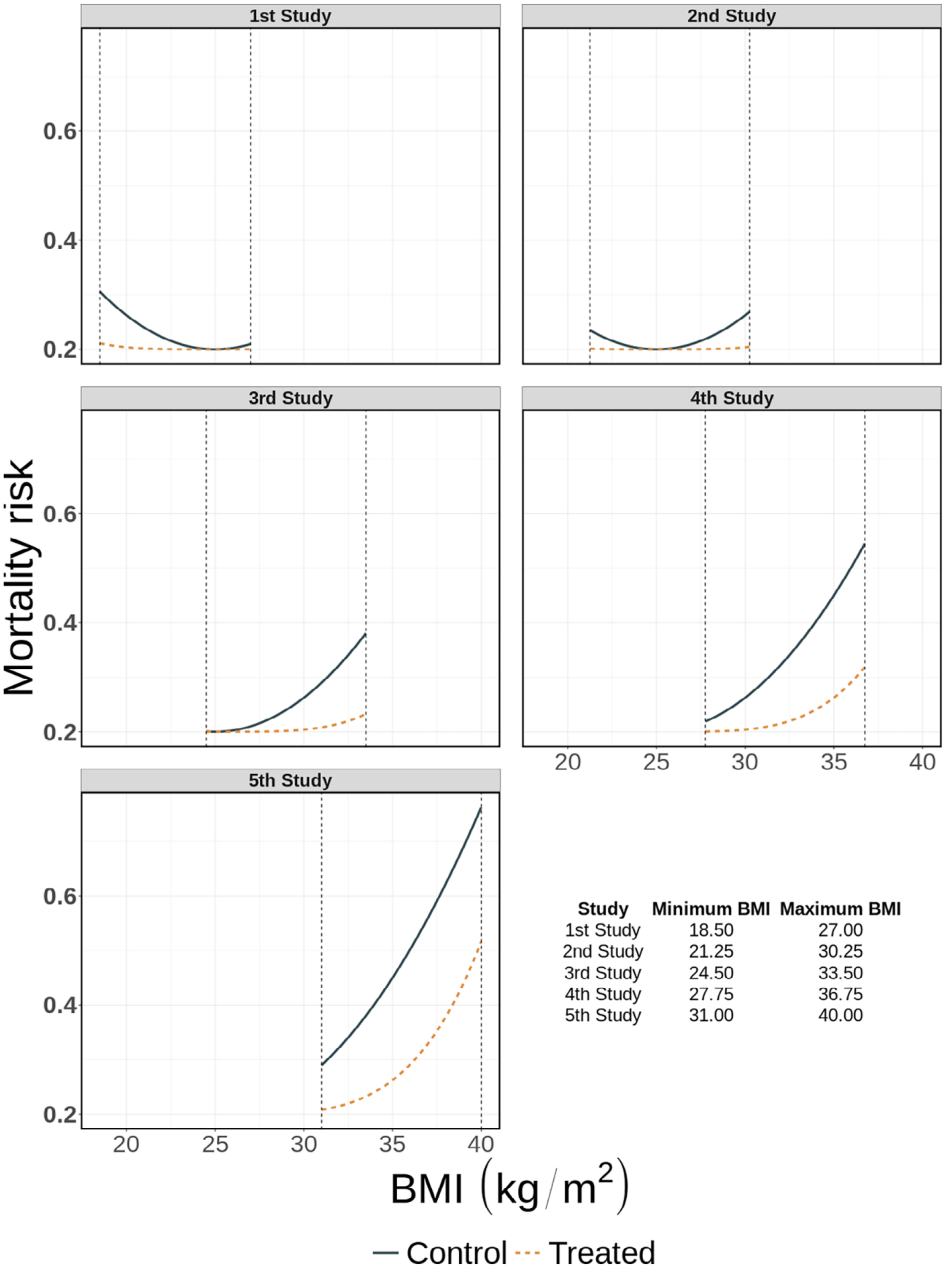
Association between mortality risk and BMI per study in the non‐heterogeneous dataset with different BMI ranges [Colour figure can be viewed at wileyonlinelibrary.com]

**FIGURE 4:**
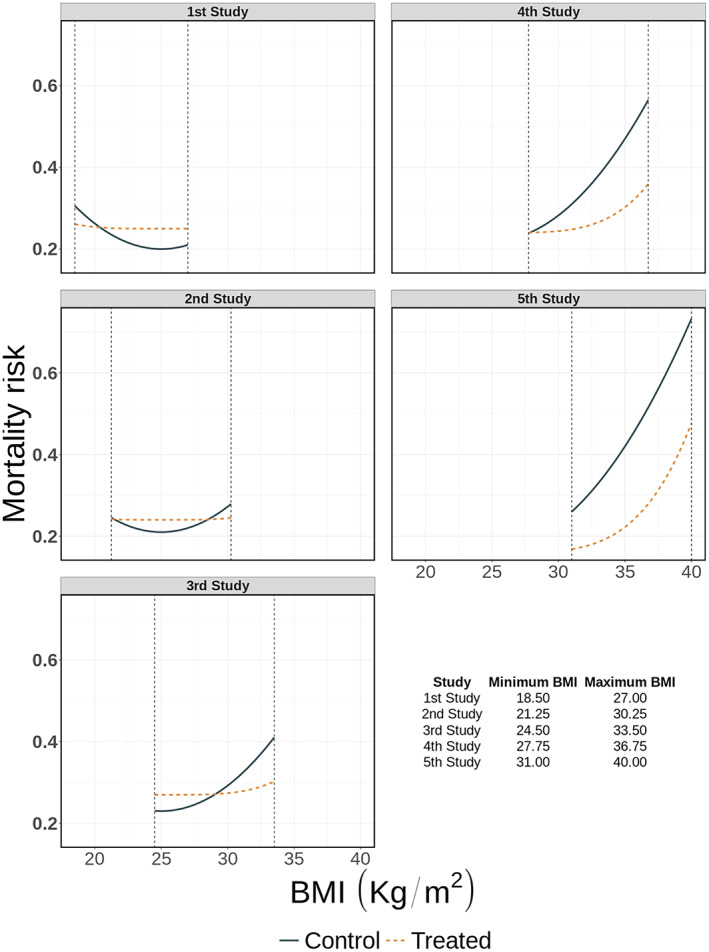
Association between mortality risk and BMI per study in the combined dataset with different BMI ranges and between study differences in the mortality risks [Colour figure can be viewed at wileyonlinelibrary.com]

To illustrate the performance of splines in IPD‐MA we generated three distinct IPD‐MA scenarios, each consisting of five RCTs comparing two interventions (1:1 randomisation) with 500 participants per study. In the first scenario, which we refer to as the heterogeneous IPD‐set with equal BMI ranges, the association between BMI and mortality is different across studies, see Figure 2, but the distribution and ranges of BMI are the same. In the second scenario, which we refer to as the non‐heterogeneous IPD‐set with different BMI ranges, the parameter values of the association for both the treated and control group are identical across all studies, but the ranges of available BMIs vary across studies (see Figure 3). In the third scenario, to which we refer as the combined IPD‐set with different BMI ranges and between study differences in mortality risks, both the ranges of BMI and the association of BMI with the mortality risk vary across studies, see Figure 4. Exact equations are given in the online Appendix sections 3 and 4.

## 3 TREATMENT EFFECT (MEASURE) MODIFICATION

“Treatment effect modification”, also called “treatment effect measure modification”^31^, ^32^ is the phenomenon where the effect of a treatment varies across the levels or strata of a certain variable. We prefer the term “treatment effect measure modification” since effect modification may be present for one measure (e.g., risk difference) but not for another (e.g. odds ratio, risk ratio).^31^, ^32^, ^33^, ^34^, ^35^ The scale in which the results are presented is therefore a vital first decision.

A commonly applied approach to investigate treatment effect measure modification is to model the interaction of a potential effect modifier with the treatment. In case of non‐linear associations, a spline‐transformed version of the continuous modifier can be used. Therefore, we model the association between the modifier and the outcome by including a spline‐transformed version of the modifier, both as main effect and in interaction with the treatment. In case of a binary outcome like mortality, a logit link function can be used in the model. In order to calculate the absolute risk difference between the treatment arms, we back‐transform the predicted outcome per treatment arm with the inverse logit function. To calculate the confidence interval of the difference in absolute risk, we use the approach proposed by Newcombe.^36^

## 4 SPLINE APPROACHES IN A SINGLE STUDY

In a setting where the association between an outcome and a continuous variable X is non‐linear, one of the options is to use splines. Splines represent a continuous variable as a linear additive combination of (often)‐local parts, which each have a simple mathematical form and are known as basis functions. Numerous basis functions have been developed involving various mathematical forms, such as polynomials, radials and Fourier series. In this paper, we focus on basis functions based on piecewise polynomials. As the term piecewise implies, the range of X is divided into intervals, using cut‐offs called knots. Within each interval, a d‐degree polynomial of X is used to model the association between the outcome Y and X. These polynomials are connected across adjacent intervals. This way, instead of estimating a global non‐linear association over the full range of data, we estimate within intervals the linear association between the outcome and transformations of X.

**FIGURE 5:**
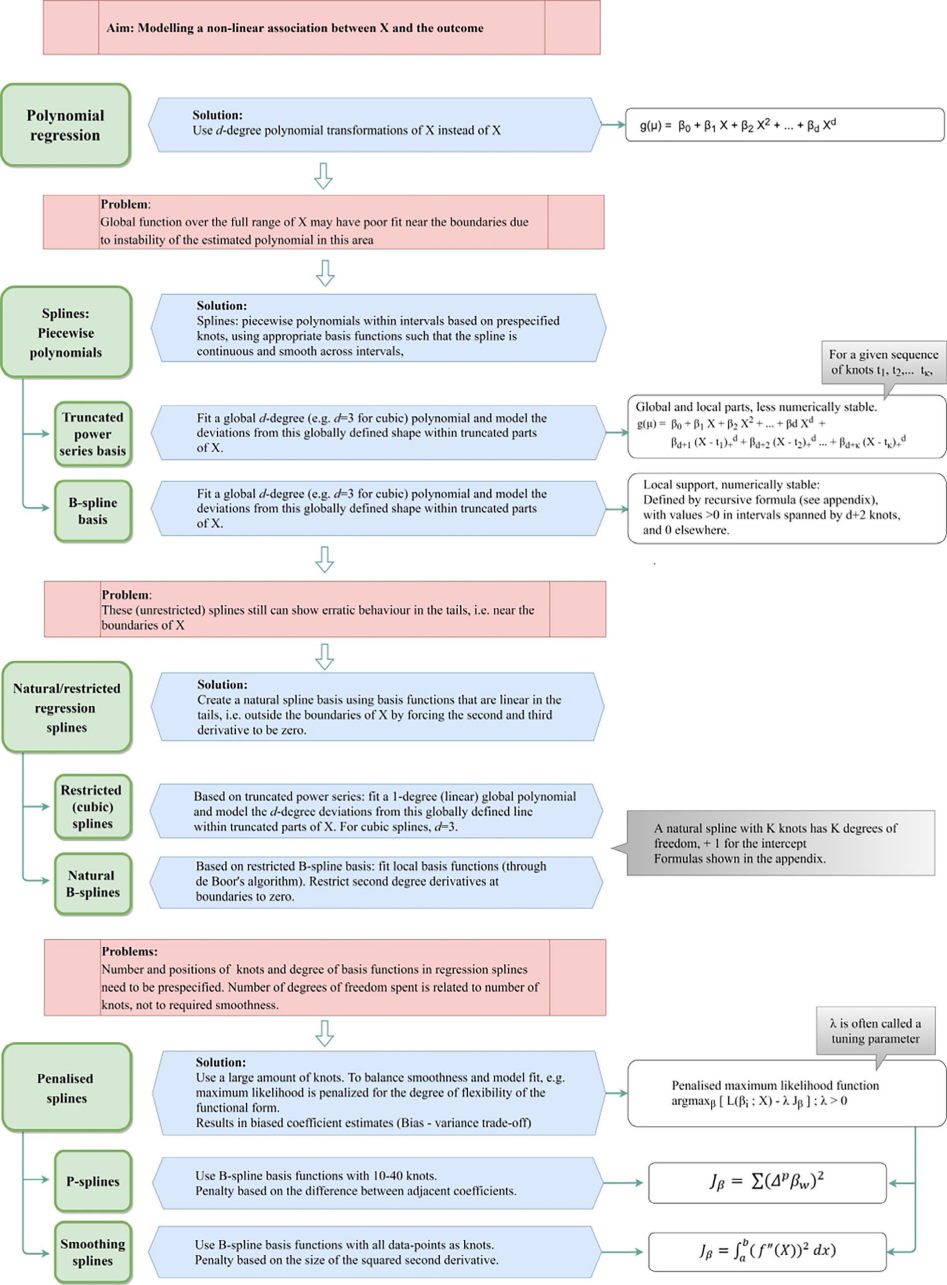
Flowchart of four types of splines [Colour figure can be viewed at wileyonlinelibrary.com]

Two important choices have to be made, in addition to the degree of the basis functions: (1) the number and the position of the knots, and (2) whether a penalty should be applied. Splines calculated with the use of pre‐specified knots and without penalties are often called regression splines. The most commonly used regression splines are restricted or natural splines^17^ and B‐splines.^18^, ^37^, ^38^ Splines where a penalty is applied are called penalised splines. The most commonly used penalised splines are P‐splines^21^ and smoothing splines.^20^ A short summary of these four types of splines is presented below. Details are presented in the online Appendix, sections 1 (Regression splines) and 2 (Penalised splines). Figure 5 shows how the aforementioned spline methods are associated with each other. Perperoglou et al. provide more details on the pros and cons of the different spline approaches.^24^

### 4.1 Regression splines

In order to understand the rationale for the use of splines based on piecewise polynomials such as the ones of interest here, we first shortly introduce global and piecewise polynomials. Global transformations of X (i.e., a function on the full range of X) include classical methods such as regular polynomials of X. Polynomials with successively higher degrees are able to capture successively more non‐linearity, but at the cost of instability, especially near the boundaries of X. The reason is twofold. First, each non‐linear part of a polynomial is more variable near the boundaries (e.g., think of a cubic function), such that small errors in the estimated coefficients have large impact. Second, each coefficient has an effect along the entire range of X, so the errors accumulate. To avoid this, polynomials fitted on different intervals of X, also called piecewise polynomials, may be preferred to global functions. Forcing the piecewise polynomials to be continuous over the knots leads to “piecewise polynomial” splines, in short “splines” (from a craftsman's tool). To accomplish continuity, several approaches have been proposed. One approach is to fit a global polynomial, whilst including terms that model the deviance from this globally defined shape within truncated parts of X, for example a truncated power series.^17^ Another approach is to locally generate transformations of X, for example B‐splines.^39^ The number and location of the knots may be based on clinical knowledge or on descriptive statistics. For instance, Harrell suggests the use of quantiles.^17^ Depending on the available sample size and required complexity of the functional shape, we may use a different number of knots.

Smoothing is improved when also the derivatives of the spline function are continuous. Typically continuity up to the second derivative offers sufficient smoothness.^24^ Since splines borrow information from adjacent intervals there still can be erratic behaviour near the (global) boundaries. To avoid this one may restrict the second derivative of the spline function to be zero at the boundaries. These splines are often called natural or restricted. Note that several types of regression splines may be combined with the “natural” property. For instance, Harrell describes a restricted truncated power series spline of third degree,^17^ Wood describes a restricted cubic spline based on cardinal basis functions,^39^ while Chambers and Hastie describe a natural B‐spline.^38^ In our manuscript, we discuss restricted splines as described by Harrell and natural B‐splines as described by Chambers and Hastie.

#### 4.1.1 Restricted splines based on truncated power series

A restricted spline based on truncated power series is constructed from a linear (first‐degree) global polynomial over the full range of X, where the deviations from this global polynomial are modelled with truncated transformations of X. We refer to truncated power series that are constrained with the “natural” or “restricted” property, as restricted cubic splines, following the terminology of Harrell.^17^

**FIGURE 6:**
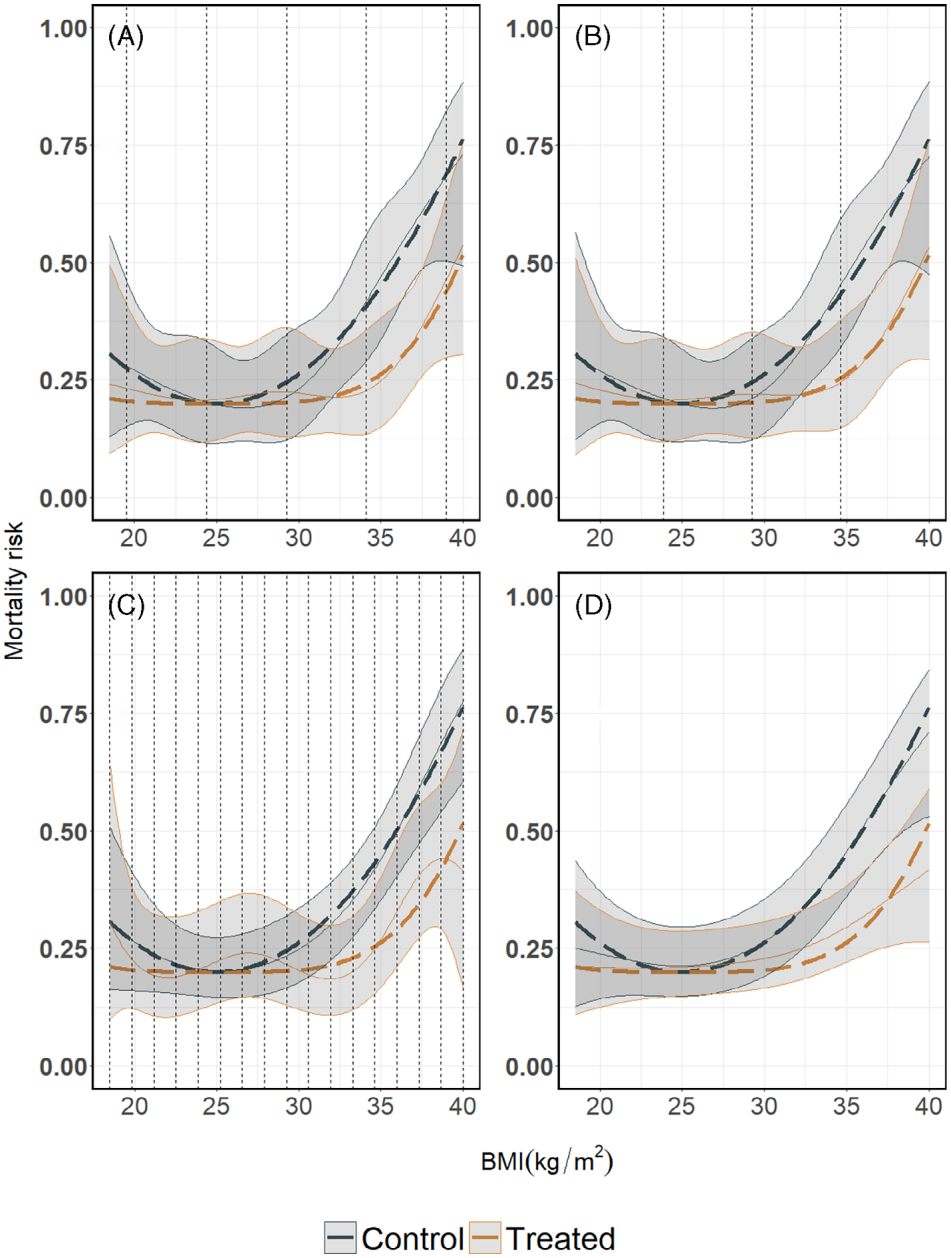
Mortality risk per treatment arm in the illustrative single study example, using (a) restricted cubic splines, (b) natural B‐splines, (c) P‐splines and d) Smoothing splines. Dashed vertical lines indicate the position of the knots. For restricted cubic splines we used five knots placed at the 5%, 27.5%, 50%, 72.5% and 95% quantiles of BMI, for the natural B‐splines we used five equidistant knots; three inner knots at BMI values 23.875, 29.250 and 34.625 plus the boundary BMI values 18.5 and 40 and for P‐splines 17 equidistant knots; 15 inner knots plus the BMI values 18.5 and 40, and for Smoothing splines as many knots as there were observations (not shown). For the penalised splines (P‐splines and Smoothing splines) the tuning parameter λ is selected through a ‘leave one out’ GCV process. Thick dashed curves indicate the true underlying mortality risk, while the thin continuous curves indicate the estimated risk [Colour figure can be viewed at wileyonlinelibrary.com]

**FIGURE 7:**
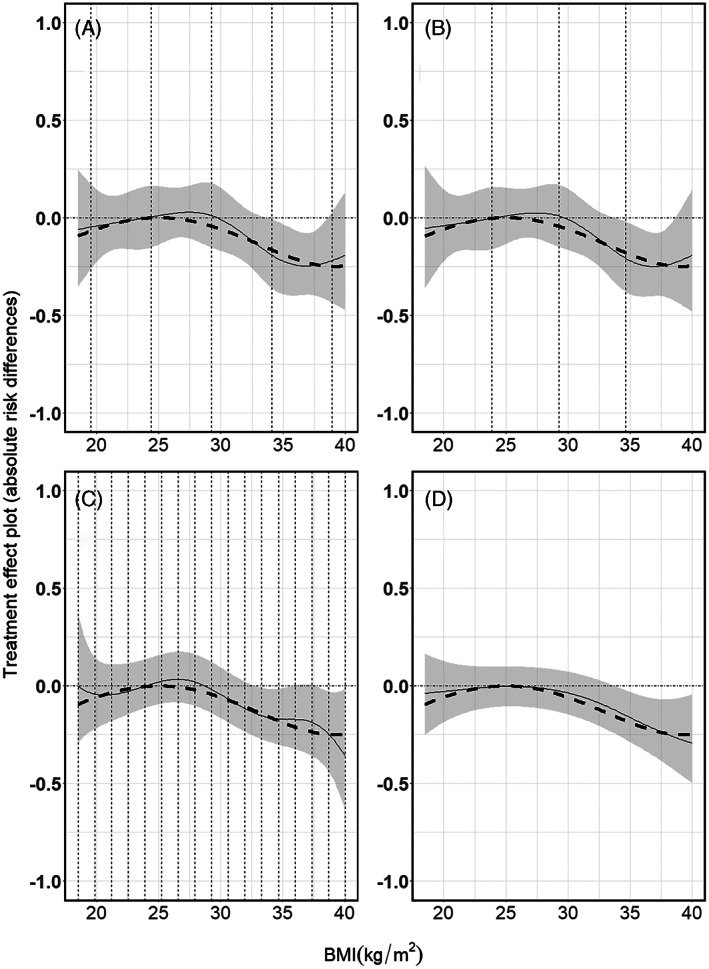
Treatment effect plots derived from subtracting the predicted risk of the experimental arm from the control arm as presented in Figure 6, estimated by (a) restricted cubic splines, (b) natural B‐splines, (c) P‐splines, and (d) Smoothing splines. Absolute treatment effects were calculated as described in section 3. The thick dashed curve is the true underlying risk difference, the thin curve is the estimated risk difference, the dashed vertical lines indicate the knot positions and the dashed horizontal line reflects the null effect (zero risk difference) line

In our single study example, we used restricted cubic spline transformations of X both as main effects and as interactions with the treatment. We placed five knots at values corresponding to 5%, 27.5%, 50%, 72.5%, and 95% quantiles of X. In Figure 6a, we present the predicted mortality risks per treatment arm, conditional on BMI, along with the 95% confidence intervals. Subsequently, we calculated the effect of the treatment conditional on BMI, by calculating the conditional risk for the control minus the conditional risk for the treated, see Figure 7a. To calculate the absolute risk difference's 95% confidence intervals, we followed the proposal of Newcombe, see section 3. Note that in our artificial data the boundaries and distribution of BMI‐values for the treated and control group are the same. In practice, this may not be true and knots may be placed at different positions for the main effects and the interaction terms.

#### 4.1.2 Natural B‐splines

B‐splines are another commonly applied regression spline approach. As opposed to the global nature of restricted cubic splines, the basis functions of B‐splines are generated locally, which improves numerical stability.^19^ We refer to B‐splines that are constrained with the “natural” or “restricted” property as natural B‐splines, following the terminology of Chambers and Hastie.^38^

In Figure 6b we show the results of the natural B‐splines approach for the simulated single study data. In order for natural B‐splines and restricted cubic splines to be comparable in terms of the degrees of freedom, we used third degree B‐spline transformations of X both for the main effects and for the interactions with the treatment. We used five equidistant knots (three inner knots at BMI plus two at the boundaries 18.5 and 40). Subsequently, we calculated the effect of the treatment conditional on BMI, see Figure 7b, similar as for the restricted cubic splines.

#### 4.1.3 Common properties of regression splines

The main advantages of regression splines are their relative simplicity and the fact that they can be represented by a formula. As a consequence, the estimated regression coefficients can be reported and used in further analysis, for example, meta‐analysis. Also both restricted splines and natural B‐splines are readily implemented in common models such as generalised linear models (GLMs) and have low computational cost. Natural B‐splines provide greater local support and numerical stability than restricted splines, since their basis functions are generated locally.^24^ Both restricted splines and natural B‐splines with κ knots require κ+1 degrees of freedom.^24^, ^38^ The main disadvantage of regression splines is that their model fit heavily depends on the number and position of the knots, thus careful modelling is required to avoid under‐ or overfitting. In some occasions, clinical knowledge on the expected curvature or descriptive statistics may be used to define the knots, but in others it is unclear how many knots should be used and where they should be placed. Commonly used criteria such as Akaike's information criterion (AIC)^40^ can be used for a databased choice of the number and position of the knots. Alternatively, penalised splines have been proposed to avoid these issues.

### 4.2 Penalised splines

The two commonly applied penalised splines that we discuss, P‐splines and smoothing splines, increase the number of knots to a large set (usually, 10–40 for P‐splines) or even to be equal to the number of observations (smoothing splines). This way they circumvent the problem of choosing the number and positions of the knots. Since estimating one parameter for each observation would clearly lead to a perfect fit and thus generate functional shapes with extreme variability, penalised splines introduce in their optimisation functions a penalty term ($J_{\beta }$) multiplied by a non‐negative λ, often called a tuning parameter. As the term, “tuning” implies, changing the value of λ changes the magnitude of the penalisation.

Penalised splines circumvent the problem of knot selection, but at a cost. By using a penalty in their optimisation function, they introduce bias in their estimate in order to obtain a more stable solution. Furthermore, in both P‐splines and smoothing splines the tuning parameter λ must be specified. Too high or too low values of λ may lead to over‐ or undersmoothing respectively. Several approaches have been proposed in order to determine the “optimal” λ, such as the AIC,^40^ “leave one out” generalised cross‐validation (GCV)^41^ or mixed‐effects modelling.^22^ These processes are automated in most of the statistical packages. Briefly, when using the AIC, a series of models fitted with different λ values are compared and the one with the lowest AIC is selected. “Leave one out” GCV is an iterative process, the algorithm goes as follows: (1) one observation is omitted, (2) a model is fitted, (3) using the model, a prediction of the omitted value is generated and (4) the distance between the observed and predicted value is calculated. This procedure is repeated for each observation and for a series of λ values. The λ that minimises the sum of the squared distances, that is, the GCV score, is selected. In Bayesian/mixed effects, modelling approaches the penalty term is estimated in a similar way as a random effects parameter.^22^ More details are given in the online Appendix section 2.

#### 4.2.1 P‐splines

A specific type of penalised splines, P‐splines, proposed by Eilers and Marx,^21^ is a penalised version of B‐splines, using a specific penalty term based on the sum of squared p‐order differences between the coefficients of two consecutive intervals $J_{\beta }=\sum {\left(\Delta^{p}\beta_{w}\right)}^{2}$. Due to this penalty, the number of basis functions and thereby the flexibility is allowed to be large, but the penalty forces adjacent coefficients to be similar when the data do not support such flexibility. The choice of the number of knots is not as important as with regression splines as long as it is sufficiently large. Note that the degree of the underlying B‐splines may be different from the order of the differences. A common combination is that of a third degree B‐spline with a second order difference. Using a penalty based on a zero‐order difference results in the ridge penalty.^42^

P‐splines are usually based on equidistant knots. While it is possible to use a knot sequence that is not evenly spaced, this would require the introduction of weights,^22^, ^24^ and they are rarely used in practice. In our single study example, we used P‐spline transformations of X for both its main effect and the interaction with treatment. We used 17 equidistant knots; 15 inner knots plus the boundaries, while the λ parameter was selected through a ‘leave one out’ GCV process as described above. In Figure 6c, we present the resulting mortality risks per treatment arm conditional on BMI, along with the 95% confidence intervals. Subsequently, the effect of the treatment conditional on BMI, calculated as the difference between the two curves in Figure 6c, is presented in Figure 7c.

#### 4.2.2 Smoothing splines

Smoothing splines are another member of the family of penalised spline methods. Similar to P‐splines, the idea is to increase the number of knots, but this time to be equal or approximately equal to the number of observations. O′ Sullivan^43^ suggested a penalty based on Reinsch's integral of the second derivative of $f\left(X\right)$, where $f\left(X\right)$ is a cubic spline.

In our single study example, we used smoothing spline transformations of X for both the main effect and its interaction with treatment, while the λ parameter was selected through a ‘leave one out’ GCV process as described above. In Figure 6d, we present the resulting mortality risks per treatment arm conditional on BMI, along with the 95% confidence intervals. The effect of treatment conditional on BMI is presented in Figure 7d.

#### 4.2.3 Common properties of penalised splines

Penalised splines reflect the belief that the predicted regression lines are more likely to be smooth than not. Therefore, their main advantage is that they are more likely to show smoother functional shapes as compared to unpenalised splines. Another advantage is that they circumvent the need to specify the positions and the number of knots, which in most cases are not known beforehand and may need to be estimated. Penalisation also affects the inference, due to the bias‐variance trade‐off. For instance, the coefficient estimates are subject to a smoothing bias, therefore their interpretation may be problematic. Note that this issue does not necessarily apply to the predicted outcomes. A related issue is that the degrees of freedom have to be modified to account for the penalisation. Wood suggests the use of effective degrees of freedom of a model. Effective degrees of freedom are calculated using the Welch‐Satterthwaite approximation formula and can be used to compare models fitted with different types of splines.^44^

## 5 INDIVIDUAL PARTICIPANT DATA META‐ANALYSIS USING SPLINES

In the previous sections, we focused on estimating non‐linear main effects and interactions with the treatment in a single study. As trials are typically not powered to investigate effect modifiers, exploring non‐linear effects in a single study may often be problematic or yield very wide confidence intervals. Depending on the underlying curvature, splines need a high amount of data and therefore their use is more feasible in the context of an IPD‐MA, where they enable the statistical modelling of complex relationships such as non‐linear associations.^45^ They can be applied in a two‐stage or one‐stage meta‐analysis approach. We apply the methods on three IPD‐MA scenarios of five studies each. In the first scenario, the underlying curves are heterogeneous whilst the BMI ranges are the same across studies. In the second scenario, the underlying curves are homogeneous but the BMI ranges are different across studies. In the third scenario, both the underlying curves and the BMI ranges are different across studies.

### 5.1 Two‐stage pointwise meta‐analysis

In pointwise meta‐analysis, a separate meta‐analysis is conducted per distinct value (point) of X, using the outcomes and standard errors as estimated per study. In the first stage of a two‐stage pointwise meta‐analysis, as proposed by Sauerbrei and Royston in method 3,^14^ we fit an appropriate model and estimate the predicted outcome per study. If needed, e.g. in case of observational data, the predicted outcomes should be controlled for individual‐level confounders by first building a confounder model.^14^ Note that instead of using fractional polynomials as in Sauerbrei and Royston, we may use any of the spline approaches described in section 4. After fitting the study‐specific models, we should decide, for example, by plotting the results, whether it is sensible to pool the predicted outcomes across studies. If so, the second stage consists of distinct meta‐analyses, for each value of X, of the study‐specific predicted values and standard errors, using either a fixed or a random effects approach.^14^ Given a continuous variable X the algorithm proceeds as follows:

#### 5.1.1 Stage 1

1. Select a spline approach and fit an appropriate model per study including interaction between X and the treatment. Since pointwise meta‐analysis pools predicted outcomes instead of model coefficients, the study‐specific models need not be the same. Different modelling techniques may be applied across studies, including linear models, fractional polynomials, and splines of different degrees and with different knot specifications. During this stage, we may use several criteria to find the best fitting model per study, for example, Aikaike information criteria, GCV or likelihood ratio tests (possibly with a nominal significance level larger than 0.05 as proposed by Sauerbrei and Royston.^14^
2. Using the models from step 1, estimate regression lines ${\hat{f}}_{T_{j}}\left(X\right)$ and $\;{\hat{f}}_{C_{j}}\left(X\right)$ for the treated and control group in study *j* respectively, along with their standard errors and 95% confidence intervals. In order to smooth the pooled regression lines from stage 2 (below), we can extrapolate the regression lines to cover the full domain of X. Automatically, the standard errors of the predicted outcomes in the extrapolated regions will be increasing along with the extent of extrapolation, and ensure small weights for the extrapolated outcomes in the meta‐analysis.
3. Depending on the outcome we wish to show and depending on the scale on which we wish to make inferences, we may choose to use a link function g and its inverse $g^{-1}$:
  1. If, in stage 2, we aim to show the predicted outcome per treatment arm and conditional on X, we calculate the predicted outcome per treatment arm, $\;g^{-1}\left({\hat{f}}_{T_{j}}\left(X\right)\right)$ and $g^{-1}\left({\hat{f}}_{C_{j}}\left(X\right)\right)$ for visualisation of the results per study. Pooling in stage 2 will be done on the scale of the link function, for example on the logit scale, per treatment arm, while in case of different ranges across the studies, the outcome is predicted for the full range (i.e., across studies) of X.
  2. If, in stage 2, we aim to show the effect of the treatment conditional on X, we first calculate per study the absolute treatment effect $g^{-1}\left({\hat{f}}_{T_{j}}\left(X\right)\right)-g^{-1}\left({\hat{f}}_{C_{j}}\left(X\right)\right)$ or the relative treatment effect $g^{-1}\;\left({\hat{f}}_{T_{j}}\left(X\right)–{\hat{f}}_{C_{j}}\left(X\right)\right),$ again over the full range of X, and calculate the corresponding confidence interval (see section 3). Note that if the goal of our meta‐analysis is to make inferences on the treatment effect, this approach is preferable to step 3.1, to avoid amalgamating the within and between study heterogeneity.^46^, ^47^

#### 5.1.2 Stage 2

**FIGURE 8:**
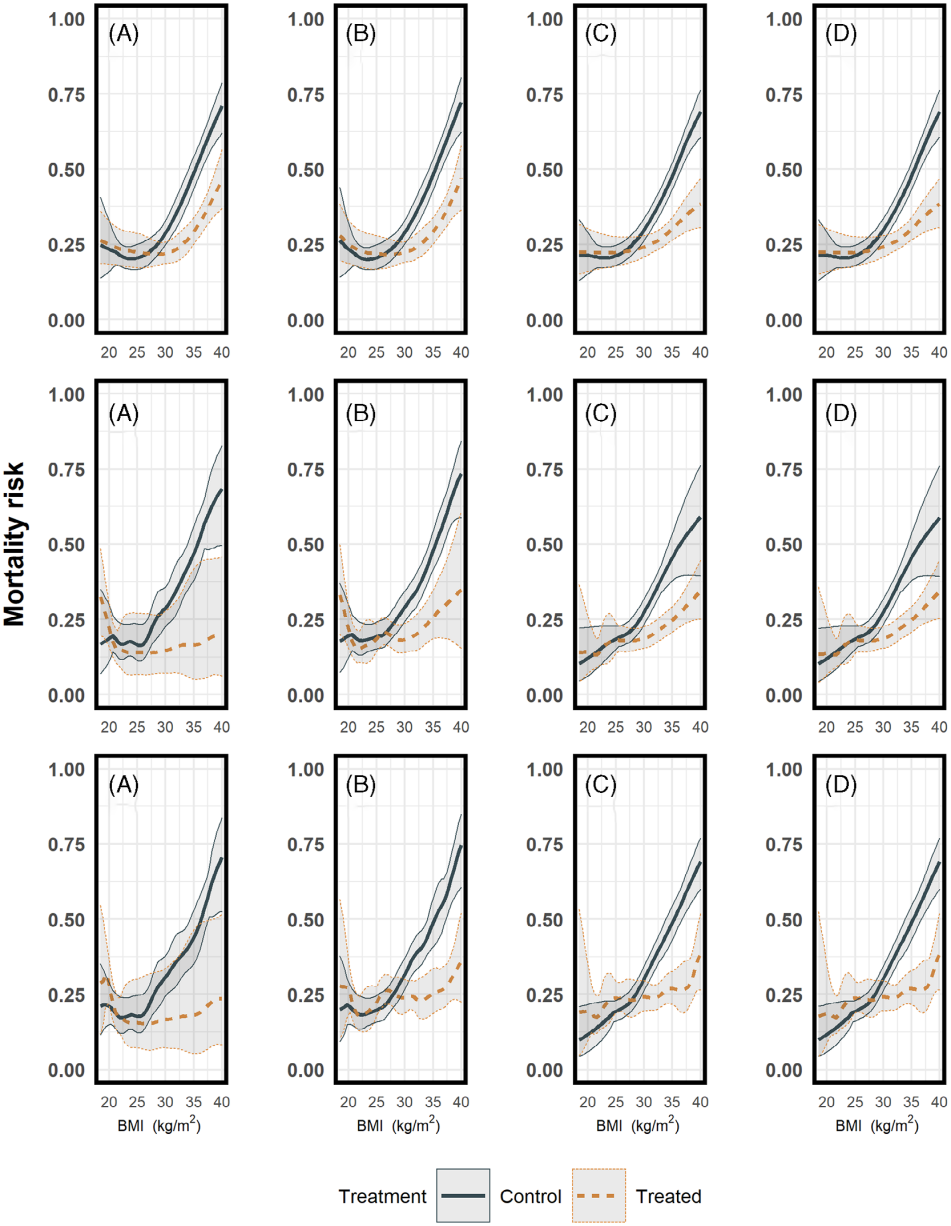
Estimated mortality risks, pooled per treatment arm using pointwise meta‐analysis. Upper row: heterogeneous dataset with equal BMI ranges; second row: non‐heterogeneous dataset with different BMI ranges; last row: combined dataset with different BMI ranges and between study differences in the mortality risks. Results from (a) restricted cubic splines, (b) natural B‐splines, (c) P‐splines, and (d) Smoothing splines. The regression lines were pooled using random effects meta‐analysis, with REML τ^2^ estimator, except for restricted cubic splines in the second and last row, where we used the DerSimonian‐Laird estimator for τ^2^ [Colour figure can be viewed at wileyonlinelibrary.com]

**FIGURE 9:**
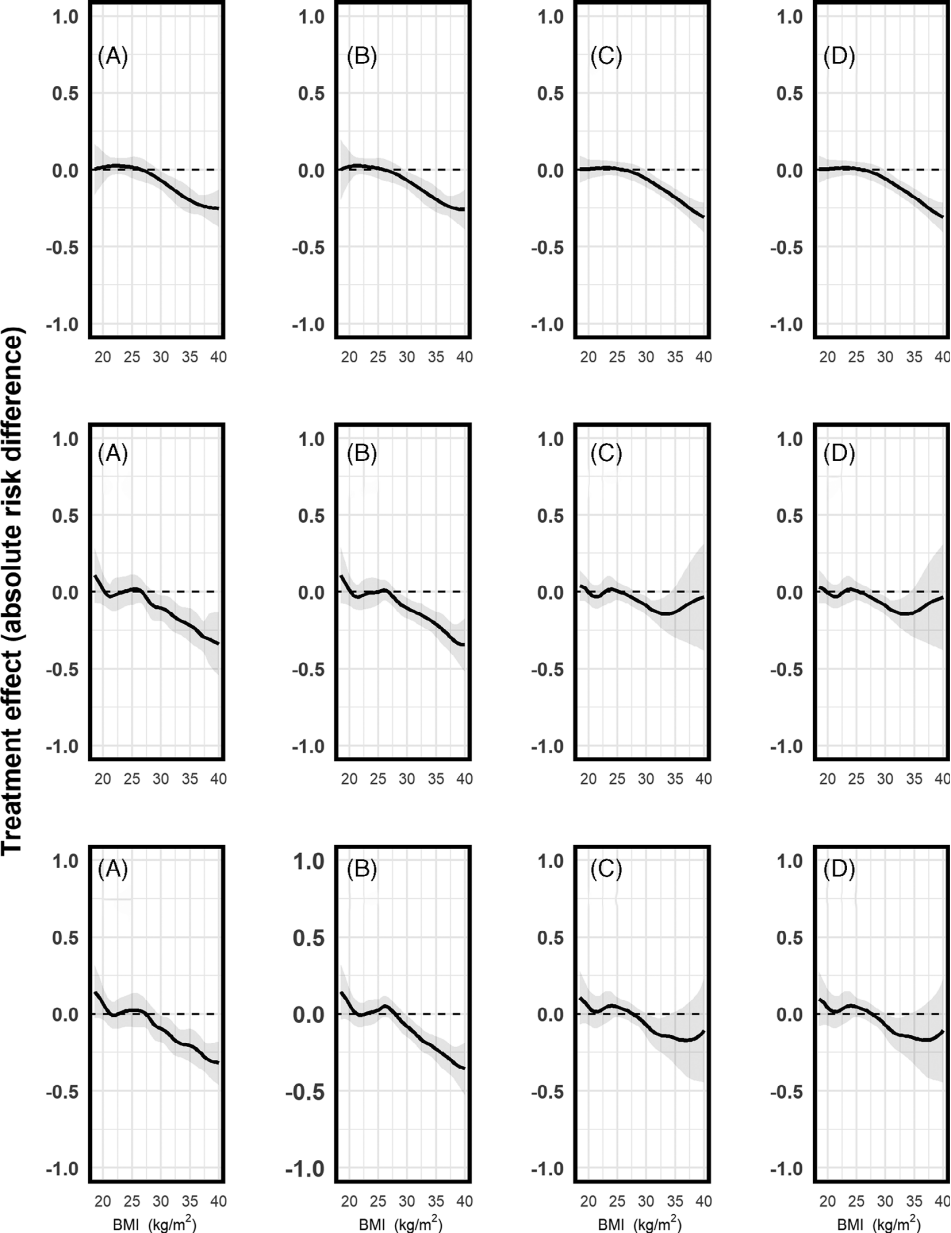
Estimated treatment effect plots, using pointwise meta‐analysis. Upper row: heterogeneous dataset with equal BMI ranges; second row: non‐heterogeneous dataset with different BMI ranges; last row: the combined dataset with different BMI ranges and between study differences in the mortality risks. Results from (a) restricted cubic splines, (b) natural B‐splines, (c) P‐splines, and (d) Smoothing splines. Treatment effects conditional to BMI were estimated per study by subtracting the regression lines for treated minus the control, as described in section 3. Subsequently, study specific treatment effects were pooled using pointwise random effects meta‐analysis, with REML τ^2^ estimator, except for P‐splines and Smoothing splines in the second and last row, where we used the DerSimonian‐Laird estimator for τ^2^

For each value within the boundaries of X we perform either a fixed or random effects meta‐analysis to get the pooled outcome of choice as a function of X along with its pointwise 95% confidence interval. Note that if the available data across the studies vary over different regions of X, pooling of the predicted outcomes may produce unsmooth results, see Figures 8 and 9, especially in the second and third scenario. From our experience, this may be caused by unsmooth estimates of the heterogeneity parameter τ across the range of X. As τ plays an important role in random‐effects meta‐analysis, it is recommended to visualise the heterogeneity estimates over the range of X.

We applied pointwise meta‐analysis using all aforementioned spline approaches in all three IPD‐MA scenarios. First, we estimated the mortality risk conditional on BMI per study and treatment arm (step 3.1), and we estimated per study the absolute risk difference and the confidence interval (step 3.2) over the full range of X. In the second stage, we pooled both the predicted curves per treatment arm (on logit scale) and their risk differences (on probability scale), using random effects meta‐analyses with REML estimators for τ^2^, except when there were problems with the convergence of the Fisher scoring algorithm in the estimation of τ^2^. In those cases, we replaced the REML estimator with the DerSimonian‐Laird estimator for τ^2^ which does not require an iterative procedure, and which in our example generally resulted in smoother estimates than REML. In a random effects meta‐analysis the size of the estimated τ^2^ affects the pooling weights of the individual studies. The pooled mortality risks per treatment arm are presented in Figure 8, and the pooled treatment effects conditional on BMI in Figure 9. Detailed figures for the third scenario, including visualisations containing study data, can be found in the online Appendix, section 5.1 for the first stage, and sections 5.2–5.5 for the second stage. Since the underlying curvature (i.e., the amount by which the curve deviates from being a straight line) per study is different across the first and the two remaining scenarios, we followed different modelling strategies for the regression splines to avoid overfitting during the first stage. For the first scenario for the restricted cubic splines, we placed four knots, following Harrell's suggestion to use the 5%, 35%, 65% and 95% quantiles of BMI and for natural B‐splines four equidistant knots (two inner knots plus the boundaries per study). For the second and third scenario, for the restricted cubic splines we placed three knots at 10%, 50% and 90% quantiles of BMI and for natural B‐splines three equidistant knots (one inner knot plus the boundaries per study. For all scenarios for P‐splines we used 17 equidistant knots (15 inner knots plus the boundaries per study). For the penalised splines (P‐splines and smoothing splines) the tuning parameter λ was selected through a ‘leave one out’ GCV process.

### 5.2 Two‐stage multivariate meta‐analysis

Instead of using pointwise meta‐analysis per distinct value of X, the functional shapes can also be pooled using multivariate meta‐analysis. This approach, as proposed by Gasparrini et al.,^23^ pools the set of regression coefficients estimated in the first stage, accounting for their within‐ and (if applicable) between‐study correlation, using a fixed or random effects multivariate meta‐analysis approach. Assumptions are a normal distribution, a constant between‐studies covariance matrix, and a normal distribution for the random effect, which implies that this is symmetrical and thus does not allow for heavy or light tails.^48^ The use of penalised splines in multivariate meta‐analysis still wants further research, e.g. with respect to possible over‐penalisation in small studies and low/no data areas within studies, and is therefore beyond our current scope. Note that in order to pool the results of the first stage, each study should provide the same set of coefficients, estimated in the same domain of X. Therefore, in order to apply multivariate meta‐analysis, the basis functions for the splines in the individual studies should be of the same degree, and also defined on the same intervals across studies, using the same knot positions. In case of different ranges of X across studies, the use of common positions for the knots may leave some coefficients inestimable in some studies and meta‐analysing them may cause complications.^23^ A solution is to conduct data augmentation as a preliminary step. Data augmentation as described by White et al.^49^ and Riley et al.^26^ refers to the generation of pseudo data beyond the per study boundaries of X, with minimal weight and arbitrary outcome. Note that in multivariate meta‐analysis a careful specification of the knots may be required. Use of a large number of knots may cause convergence issues during the second stage, in case of a rank‐deficient variance covariance matrix.

The multivariate meta‐analysis algorithm proceeds as follows:

#### 5.2.1 Stage 1

1. As a preliminary step, choose the knots corresponding to the optimal locations across the studies along with the degree of the unpenalised spline.
2. Per study j, fit a model including interaction between X and the treatment with the chosen specifications of step 1.
3. With Q the total number of coefficients and q ∈ [1, 2, …, Q], extract per study the estimated coefficients ${\hat{\beta }}_{\mathrm{qj}}$ along with their variance–covariance matrix.

#### 5.2.2 Stage 2

1. Use either fixed or random effects multivariate meta‐analysis to estimate the pooled ${\hat{\beta }}_{q}$
2. To calculate the predicted outcome given X and treatment T, multiply the pooled estimates with the design (or model) matrix containing the values of X along with their spline transformed values.
3. To estimate the treatment effect conditional on X, subtract the pooled‐per‐treatment arm outcomes and calculate the confidence interval as described in section 3.

**FIGURE 10:**
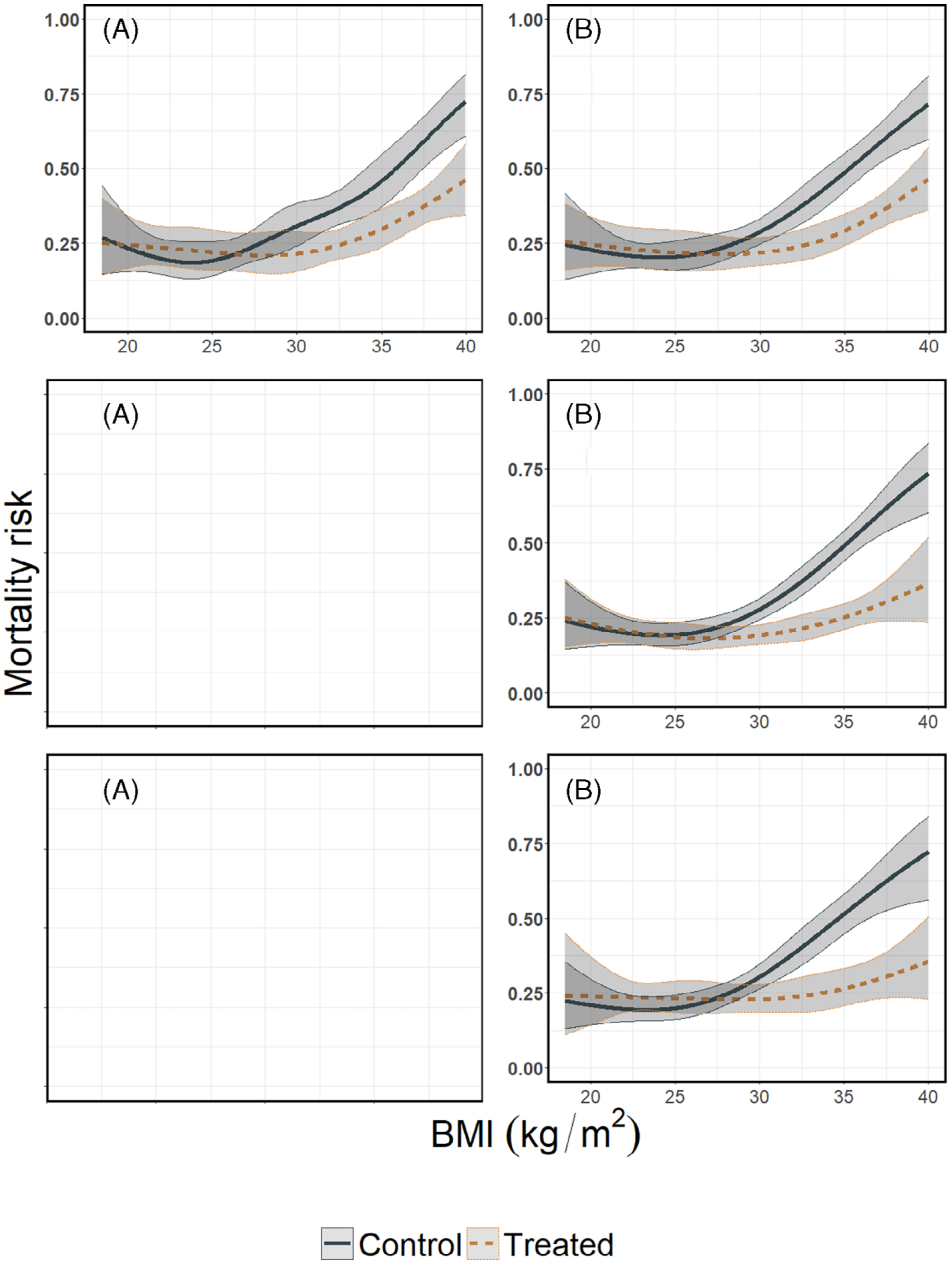
Estimated mortality risks per treatment arm conditional on BMI, pooled using multivariate metaanalysis. Upper row: heterogeneous dataset with equal BMI ranges; second row: non‐heterogeneous dataset with different BMI ranges; last row: the combined dataset with different BMI ranges and between study differences in the mortality risks. Results from (a) restricted cubic splines and (b) natural B‐splines. Multivariate meta‐analysis with restricted cubic splines failed to converge for the second and third scenario. The regression lines were pooled using random effects meta‐analysis, with REML variance covariance matrix estimator [Colour figure can be viewed at wileyonlinelibrary.com]

**FIGURE 11:**
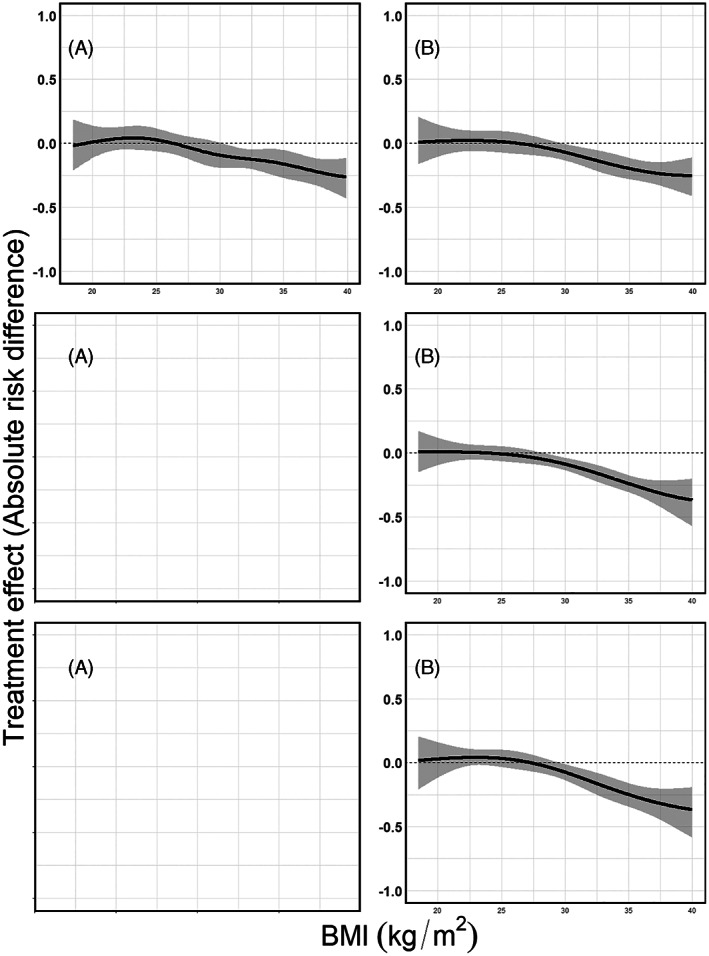
Treatment effect plots using multivariate meta‐analysis. Upper row: heterogeneous dataset with equal BMI ranges; second row: non‐heterogeneous dataset with different BMI ranges; last row: the combined dataset with different BMI ranges and between study differences in the mortality risks. Results from (a) restricted cubic splines, and (b) natural B‐splines, in the heterogeneous dataset with equal BMI ranges. Multivariate meta‐analysis with restricted cubic splines failed to converge for the second and third scenario. Absolute treatment effects along with their confidence intervals were calculated as described in section 3, using the pooled mortality risk for treated and control and their confidence intervals as shown in Figure 10. The dashed horizontal line shows the null effect (zero risk difference) line

We applied multivariate meta‐analysis in combination with regression splines in all three scenarios. To do so we performed data augmentation as a preliminary step^26^, ^49^ in the second and third scenario. This way all studies had curves estimated over the full range of BMI. In stage 1, we fitted restricted cubic spline and B‐spline transformations of BMI both as main effects and as interactions with the treatment per study. For the restricted cubic spline transformations, we used five knots, following Harrell's suggestion to use the 5%, 27.5%, 50%, 72.5% and 95% quantiles of BMI, for natural B‐splines four equidistant knots (two inner knots plus the boundaries per study). Note that we positioned the knots over the full domain of BMI. Subsequently, we pooled the estimated coefficients using a random‐effects meta‐analysis with the REML estimation method. We calculated regression lines per treatment arm by multiplying the design (or model) matrix with the pooled coefficients. Absolute risk differences were calculated by subtracting the pooled mortality risks, conditional on the covariables, of the treated minus the control, while for the confidence intervals we used the proposal of Newcombe.^36^ In the second and third scenario, multivariate meta‐analysis failed to converge for the restricted cubic splines approach. For restricted cubic splines based on truncated power series, some of the basis functions are defined over the whole range of data and others on truncated parts only. To apply multivariate meta‐analysis, the same type of spline needs to be fitted over the same data range in all individual studies. In studies with a limited range of data, data augmentation in combination with a restricted cubic spline basis might lead to high correlations between some basis splines, implying numerical instabilities in spline estimation. Therefore, for these scenarios we present only results for natural B‐splines. The pooled mortality risks per treatment arm are presented in Figure 10, and the pooled treatment effects conditional on BMI in Figure 11. A visualisation of the models per study using the natural B‐splines in the third scenario can be found in the online Appendix, section 5.6.

### 5.3 One‐stage generalised additive mixed effects model

Instead of using a two‐stage meta‐analysis, we may also conduct the analysis in one stage, using a mixed effect model with splines, that is, a generalised additive mixed effect model (GAMM). Hereby, we may include spline transformations of X as main effects and as interactions with the treatment as described in section 3. Note that spline transformations of X are the sum of several basis functions, as described in section 4. The effects of the basis function may be common to all studies, stratified across studies, or allowed to have study‐specific deviations from the overall effect that follow a (usually) normal distribution (i.e., random effects). Common effects may be modelled straightforward by including the basis functions as they are. Stratified effects can be modelled by including an interaction between the basis function and the (categorical) clustering variable (study in this context). Random effects can be modelled by penalising the interaction of the basis function with the clustering variable, as Wood^22^, ^50^, ^51^ and Kimeldorf and Wahba^52^ have shown.

Depending on the estimand of choice and the assumptions researchers wish to make they may use any combination of the above assumptions for their model. On the one side, full stratification provides least bias at the cost of high variance (i.e., high uncertainty with respect to the coefficients). On the other side, a model with only common effects has most bias and least uncertainty with respect to the parameters. A model utilising random effects will be somewhere in‐between since the variability across studies is forced to follow a certain distribution, which will shrink study‐specific estimates to the overall mean. Note that interaction terms included in one‐stage mixed effect models may be prone to ecological bias and amalgamate the within and across study effects.^46^, ^53^, ^54^, ^55^ This is easily seen from the random effect mechanism, which shrinks study specific estimates towards the overall mean. To model ecological bias in this setting, two methods have been proposed. One approach is to stratify by study all or some of the main effects including at least the treatment effect.^26^ Another approach is to centre the covariable X around its study‐specific mean $\bar{X_{j}}$ creating a new variable $Z=X-\bar{X}$_j_. Subsequently, include Z, $\;\bar{X}$_j_, and the interaction of Z with the treatment in the one‐stage model.^26^, ^56^ While these methods have been demonstrated to be sufficient in presence of linear effects, adequate modelling choices to prevent introduction of ecological bias in presence of non‐linear relations are still ongoing research.

**FIGURE 12:**
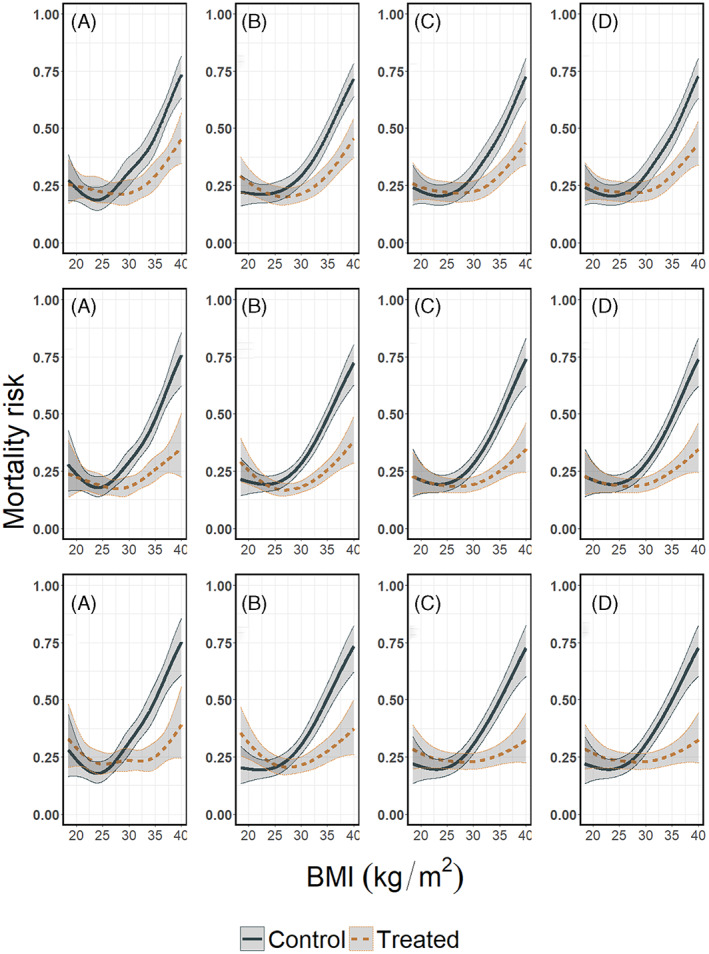
Estimated mortality risks per treatment arm conditional on BMI, pooled using a random effects GAMM. Upper row: heterogeneous dataset with equal BMI ranges; second row: non‐heterogeneous dataset with different BMI ranges; last row: the combined dataset with different BMI ranges and between study differences in the mortality risks. Results from (a) restricted cubic splines, (b) natural B‐splines, (c) P‐splines, and (d) Smoothing splines. For penalised splines the λ parameter is selected through a ‘leave one out’ GCV process [Colour figure can be viewed at wileyonlinelibrary.com]

**FIGURE 13:**
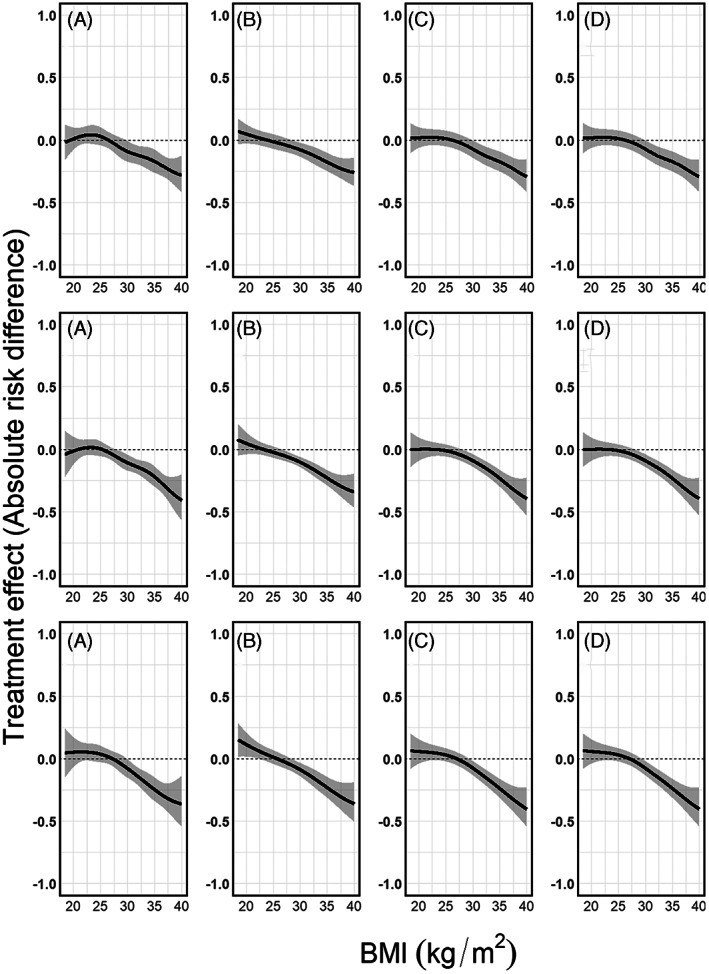
Treatment effect plots using GAMMs. Upper row: heterogeneous dataset with equal BMI ranges; second row: non‐heterogeneous dataset with different BMI ranges; last row: the combined dataset with different BMI ranges and between study differences in the mortality risks. Results from (a) restricted cubic splines, (b) natural B‐splines, (c) P‐splines, and (d) Smoothing splines. Absolute treatment effects along with their confidence intervals were calculated as described in section 3, using the pooled mortality risk for treated and control and their confidence intervals as shown in Figure 12. The dashed horizontal line shows the null effect (zero risk difference) line

In our three scenarios, we used the four aforementioned spline transformations both as main effect and in interaction with the treatment. We used a random intercept and random slope for BMI in combination with a fixed spline part. For the restricted cubic splines, we used five knots (the 5%, 27.5%, 50%, 72.5% and 95% quantiles of BMI), for natural B‐splines four equidistant knots (two inner knots plus the boundaries), and for P‐splines we used 17 equidistant knots (15 inner knots plus the boundaries). Note that we positioned the knots over the full domain of BMI and that no manually conducted data‐augmentation nor extrapolation was needed. The pooled mortality risks per treatment arm are presented in Figure 12, and the pooled treatment effects conditional on BMI in Figure 13. A visualisation of the estimated models per study using the restricted cubic splines in combination with GAMM in the third scenario can be found in the online Appendix, section 5.7.

### 5.4 Properties of the pooling methods

We illustrated the association between BMI and mortality risk in three scenarios using four spline methods and two two‐stage approaches (pointwise and multivariate meta‐analysis) and one one‐stage approach (GAMM). Table 1 summarises the properties of the aforementioned approaches. An advantage of two‐stage approaches is that they provide insight into between‐study variability during the first stage. This is easily overlooked when starting from a one‐stage model. Furthermore, in two‐stage methods we may use heterogeneity measures such as Cochran's Q statistic, τ^2^, and prediction intervals per value of X to assess whether it is sensible to pool the associations. Therefore, it is always informative to start with investigating the results per study, similar to the first stage in two‐stage methods. However, two‐stage methods may not always be feasible when some studies are too small with respect to model complexity, that is, when the number of parameters that needs to be estimated is large compared to the number of observed events (e.g., the number of deaths).

**TABLE 1:** Comparison of the pooling methods in IPD‐MA

Characteristic	Pointwise meta‐analysis	Multivariate meta‐analysis	GAMM
Input for pooling	Predicted outcome per value of X	Coefficients estimated per study	One‐stage approach using crude data. Uses mixed effects modelling to account for within study clustering
Allows for different model specifications (functional forms) across studies	Yes	No	No
Allows for different ranges of X across studies	Yes, by using the study‐specific models to predict outcomes over the full range of X. (Extrapolation) Be cautious; in case of random‐effects meta‐analysis the pooled functional curve may be overly influenced by the extrapolated estimates.	Yes, by adding observations with small weights at the extremes of X. (Data augmentation) Further research is needed on how to apply restricted splines based on truncated power series in case of highly heterogeneous domains of X.	Yes, a single model is specified across all values of X
All types of splines can be used	Yes	No Further research is needed on how to apply penalised splines.	Yes
Main advantages	Flexible as it allows study‐specific modelling, that is, different models and splines across studies. Insightful, as the translation from study‐based curves to the pooled curve is a straightforward random‐effects meta‐analysis per value of X.	More efficient (i.e., smaller confidence intervals) than pointwise meta‐analysis, if the same model across studies can be assumed and if this model is specified correctly. Pooled curves are represented by a formula.	Can handle different study domains and small sample sizes. More efficient than pointwise meta‐analysis, if the same model across studies can be assumed and if this model is specified correctly, taking into account between‐study heterogeneity and ecological bias. Pooled curves are represented by a formula.
Main disadvantages	Pooled curves can be less smooth than with multivariate meta‐analysis or GAMM. Pooled curves are not represented by a formula, thus there are no pooled regression coefficients to be reported and used in further analyses.	Highly sensitive to modelling choices. Inflexible as it needs the same model specifications across studies, including type of spline and number and positions of knots, and assumes a constant heterogeneity matrix. Model complexity is limited by the smallest study.	Requires careful assessment of between study variability since this is not automatically provided, thus careful modelling is required.

### 5.5 Pointwise meta‐analysis: Robust and flexible but non‐smoothness may occur

The main advantages of pointwise meta‐analysis are its flexibility, robustness (i.e., have less error) and ease of use. In particular, pointwise meta‐analysis is flexible since we are allowed to fit different models across the studies, as we are pooling the predicted outcomes rather than the coefficients. As an extreme example, in one study, we may apply a restricted cubic spline transformation of X, in another a second degree B‐spline and in another we may choose to not transform X. Such differences are more likely to occur when studies greatly differ in sample size and in their baseline characteristics, (e.g., more narrow covariate range). Also we are allowed to vary the number and position of knots per study. As we are fitting per study a best fitting model, pointwise meta‐analysis is also robust to model misspecification. When the ranges of X are different across studies, pointwise meta‐analysis uses the whole domain of X, even without data augmentation, by means of extrapolation. The main disadvantage of pointwise meta‐analysis is that the pooled curve may not be smooth. This might be related to non‐smooth estimates of heterogeneity across the range of X (see also Figures 5.2.A–5.5.A in the online Appendix sections 5.2–5.5, which show the estimated τ across the range of BMI). Especially in case of extrapolation, the variation between the predicted study outcomes might be increased. Even though the corresponding SEs are also larger, we noted that the between‐study heterogeneity can have a relevant effect on the study weights, which in a random‐effects meta‐analysis are based on a combination of SE^2^ and the between study heterogeneity τ^2^. Consequently, a random effects meta‐analysis may give disproportionate weight to the extrapolated outcomes. Hence, attention should be payed to the role of the between‐study heterogeneity in the estimation of the pooled functional form. Also since we are performing a meta‐analysis for each value of X, pointwise meta‐analysis may be more computationally intensive and show wider confidence intervals than multivariate meta‐analysis and GAMMs. Finally, the pooled curves are not represented by a formula, thus there are no pooled regression coefficients to be reported and used in further analysis. This is especially inconvenient when combinations of X are of interest.

### 5.6 Multivariate meta‐analysis: efficient if specified “correctly”, but lacks robustness and flexibility

White et al. show that the main advantage of multivariate meta‐analysis is that multivariate meta‐analysis appears to be more efficient, with narrower confidence intervals, than pointwise meta‐analysis if the model is correctly specified.^25^ However, this argument may not be relevant in practice, since models cannot be expected to be correctly specified in general. For instance, in our illustrative examples we generated quadratic and quartic associations for the control and treated group, respectively. However, during the analysis we used cubic splines to model these associations and the resulting curves were not a perfect representation of the pre‐specified curves but rather an approximation, thus our model may be considered mis‐specified. A major limitation of multivariate meta‐analysis is that it is highly sensitive to model specification. In the first scenario, the multivariate meta‐analysis had similar efficiency as pointwise meta‐analysis and GAMM. But in the second and third scenario with the different ranges across the studies, multivariate meta‐analysis showed results only for natural B‐splines, see Figures 10 and 11. Furthermore, it is less flexible compared to pointwise meta‐analysis, since the models fitted per study should have the same parametrisation e.g. same type of spline, same number and positions of knots and the same range of X.^23^ This restriction may be problematic in cases where a subset of studies included in the meta‐analysis has a limited number of participants. In that case, modelling the association between the outcome and the spline transformations of X may fail to converge for a subset of studies, and only multivariate meta‐analysis based on simple linear models may be possible. Model complexity is thus limited by the smallest study. Furthermore, it is assumed that the heterogeneity matrix is constant across X Finally, since multivariate meta‐analysis pools the coefficients estimated during the first stage, it may not be compatible with approaches where penalisation to those coefficients is applied.

### 5.7 GAMM can handle different study domains and sample sizes, whilst producing smooth pooled regression curves, but careful modelling is required

The main advantage of GAMMs is that they can more easily handle differences in the distributions (ranges) of X across studies, similar to pointwise meta‐analysis, and include all studies regardless of the number of observations. In contrast to pointwise meta‐analysis, GAMMs result in smooth pooled curves and confidence intervals, also in case of differences in measured domains of X across studies. The main disadvantage of GAMMs is that they do not automatically provide insight into the full amount of heterogeneity between studies as provided in two‐stage methods. Therefore, if the data allow, it is advisable to fit study‐level models anyway to inform the one‐stage model. Furthermore, since GAMMs are one‐stage mixed effects models similar to generalised linear mixed models, they require careful modelling while taking into account between‐study heterogeneity. Caution is especially important when aggregation (ecological) bias might be present, as discussed by Riley et al., Hua et al., and Belias et al.^26^ ^47^, ^56^

## 6 SOFTWARE

All analyses were performed with the statistical software R version 3.6.0. For data manipulation we used the tidyverse^57^ package, for the restricted cubic splines we used rms^58^ package, for natural B‐splines we used the splines package included in base R and for P‐splines, smoothing splines and GAMMs we used the mgcv^50^ package. For pointwise and multivariate meta‐analysis we used the meta^59^ and mvmeta^23^ packages respectively. Currently, package meta does not provide a function to conduct pointwise meta‐analyses over a grid of X values, but the provided R‐code shows how to implement this. It is also possible to estimate splines in other software such as Stata or SAS. However, since R is freely available for every researcher, we provide the scripts to apply splines in multiple studies scenarios only in R, using the third illustrative scenario as an example.

## 7 EMPIRICAL EXAMPLE

To illustrate the use of splines combined with the aforementioned pooling methods in a real example we consider a previously published IPD‐MA investigating the effect of antibiotics in children with AOM.^28^ Rovers et al. collected IPD from six randomised clinical trials with a total of 1643 children, aged from 0 to 12 years old. The primary outcome was fever and/or ear‐pain 3–7 days (yes/no) after antibiotics or placebo treatment. They concluded that treatment was efficacious in young children (till 2 years old) with bilateral AOM. Below, we investigate the effect of antibiotics across the values of age, in children with unilateral or bilateral AOM.

### 7.1 Methods

Data from one study were omitted as information on unilateral or bilateral AOM was not reported. We used data of children till 9 years old, as AOM above 9 years is rare and we had a limited number of children over that age (only 15). From the remaining five studies, one study (Appelman et al.) had a limited number of events (children with fever/ear pain) and for some age‐bilateral AOM combinations no events at all. Therefore, we followed different strategies across the pooling methods for this study.

#### 7.1.1 Pointwise meta‐analysis

For pointwise meta‐analysis, for the Appelman study we fitted a logistic regression model including the main effects of bilateral AOM, treatment and age and their two‐by‐two interactions, without any spline transformation for age. For the remaining studies we fitted per study a logistic regression model including the main effects of bilateral AOM, treatment, and age transformed by each of the aforementioned spline approaches, and we included the interactions of the spline transformed age with bilateral AOM (yes/no) and treatment (both two‐way). For restricted cubic splines we followed Harrell's suggestion and used per study three knots at the 10%, 50% and 90% quantiles of age; for natural B‐splines we used third degree basis functions and per study three equidistant knots (1 inner knot plus the boundaries per study), while for P‐splines we used third degree basis functions and per study 17 equidistant knots (15 inner knots plus the boundaries per study). For the penalised splines (P‐splines and smoothing splines) the tuning parameter λ was selected through a ‘leave one out’ GCV process. Subsequently, we extracted the predicted outcomes for fever/ear pain in logit scale and pooled them using a random‐effects meta‐analysis approach with REML τ^2^ estimator. To show the pooled risk conditional on children's age, bilateral AOM and treatment group, we back‐transformed the pooled curves into risk curves. To show the treatment effect conditional on children's age and bilateral AOM, we first back‐transformed per study the predicted fever/ear pain risk. To estimate per study the absolute risk difference between the treated and control along with their confidence intervals, we followed the proposal of Newcombe,^36^ see Section 3. Finally, we pooled the risk differences using a random‐effects meta‐analysis approach with the REML τ^2^ estimator.

#### 7.1.2 Multivariate meta‐analysis

For multivariate meta‐analysis, we omitted the Appelman study mentioned above. Therefore, multivariate meta‐analysis was based on the four remaining studies with a sufficient number of observations to fit splines. Also, since in multivariate meta‐analysis the ranges of age across the studies need to be the same, we performed data‐augmentation as a preliminary step. In the first stage of the multivariate meta‐analysis, we fitted a logistic regression model including the main effects of treatment, bilateral AOM and spline transformed age, and the two‐way interactions of spline transformed age with treatment and bilateral AOM. Since in multivariate meta‐analysis the positions of knots need to be the same across the studies, for restricted cubic splines we used three knots at 10%, 50%, 90% quantiles of age calculated on the four studies combined; for natural B‐splines we used third degree basis functions and three equidistant knots (one inner knot at age 2.5 plus two at the boundaries of age). Subsequently, we extracted the regression coefficients and their variance–covariance matrix and pooled them using a random‐effects meta‐analysis approach with REML estimator for τ^2^. Finally, to show the risk of developing fever/ear pain conditional on age, treatment and bilateral AOM, we multiplied the pooled coefficients with the corresponding design matrix and back‐transformed the pooled outcomes using the inverse logit function. To calculate the absolute risk differences and their confidence intervals, we followed the proposal of Newcombe,^36^ see section 3.

#### 7.1.3 Generalised additive mixed effects models

For GAMMs, we included all five studies. We fitted a logistic regression model including the main effects of treatment, bilateral AOM and spline transformed age and the two‐way interactions of spline transformed age with treatment and bilateral AOM. We used similar definitions for knot positioning and degrees of splines as in pointwise and multivariate meta‐analysis, but using the whole data‐set without data‐augmentation nor extrapolation. We followed Wood's proposal and included random‐effects for the intercept and for the slope of age additively to account for the within study clustering of participants.^39^

### 7.2 Results

**FIGURE 14:**
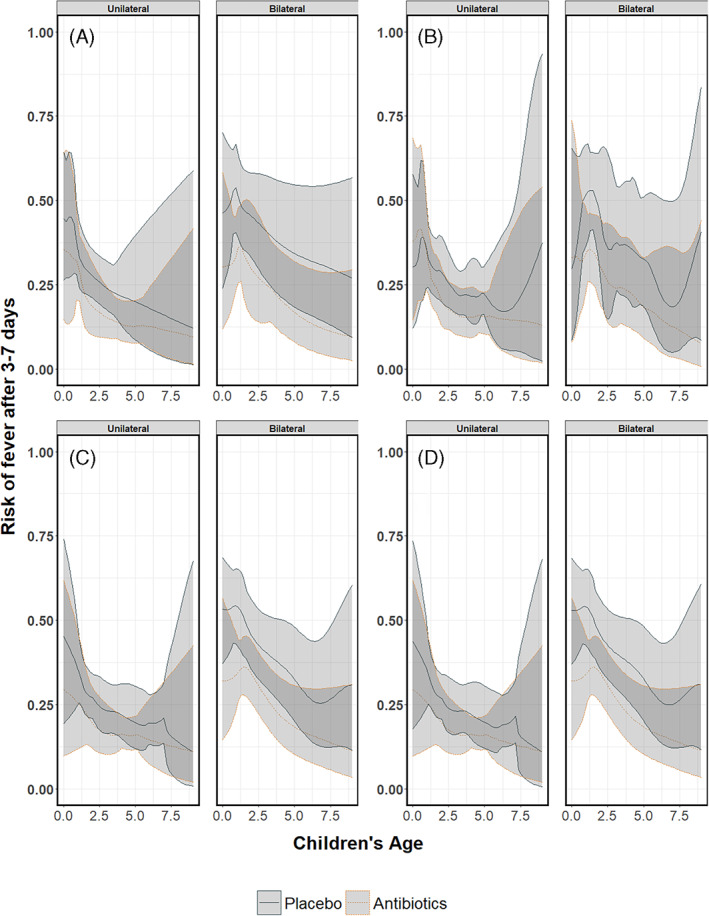
The risk of having fever/ear‐pain after 3–7 days in children with either unilateral or bilateral acute otitis media receiving either antibiotics or placebo. The presented results are estimated using pointwise meta‐analysis with (a) restricted cubic splines, (b) natural B‐splines, (c) P‐splines and (d) Smoothing splines [Colour figure can be viewed at wileyonlinelibrary.com]

**FIGURE 15:**
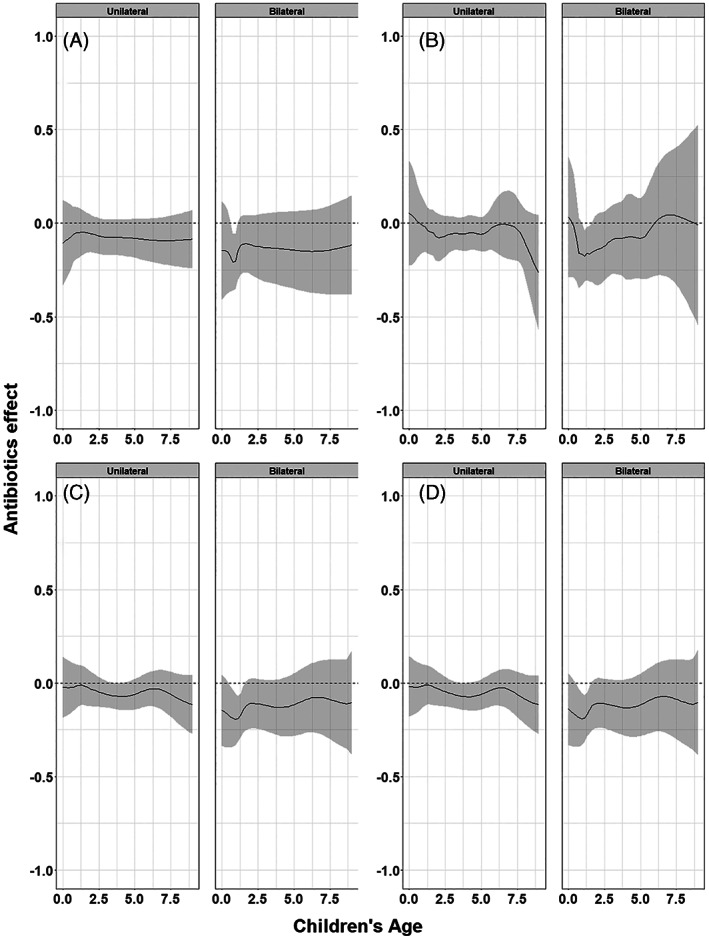
The antibiotics effect (risk difference of having fever/ear‐pain after 3–7 days) in children with either unilateral or bilateral acute otitis media, conditional on age. The presented results are estimated using pointwise meta‐analysis with (a) restricted cubic splines, (b) natural B‐splines, (c) P‐splines and (d) Smoothing splines

**FIGURE 16:**
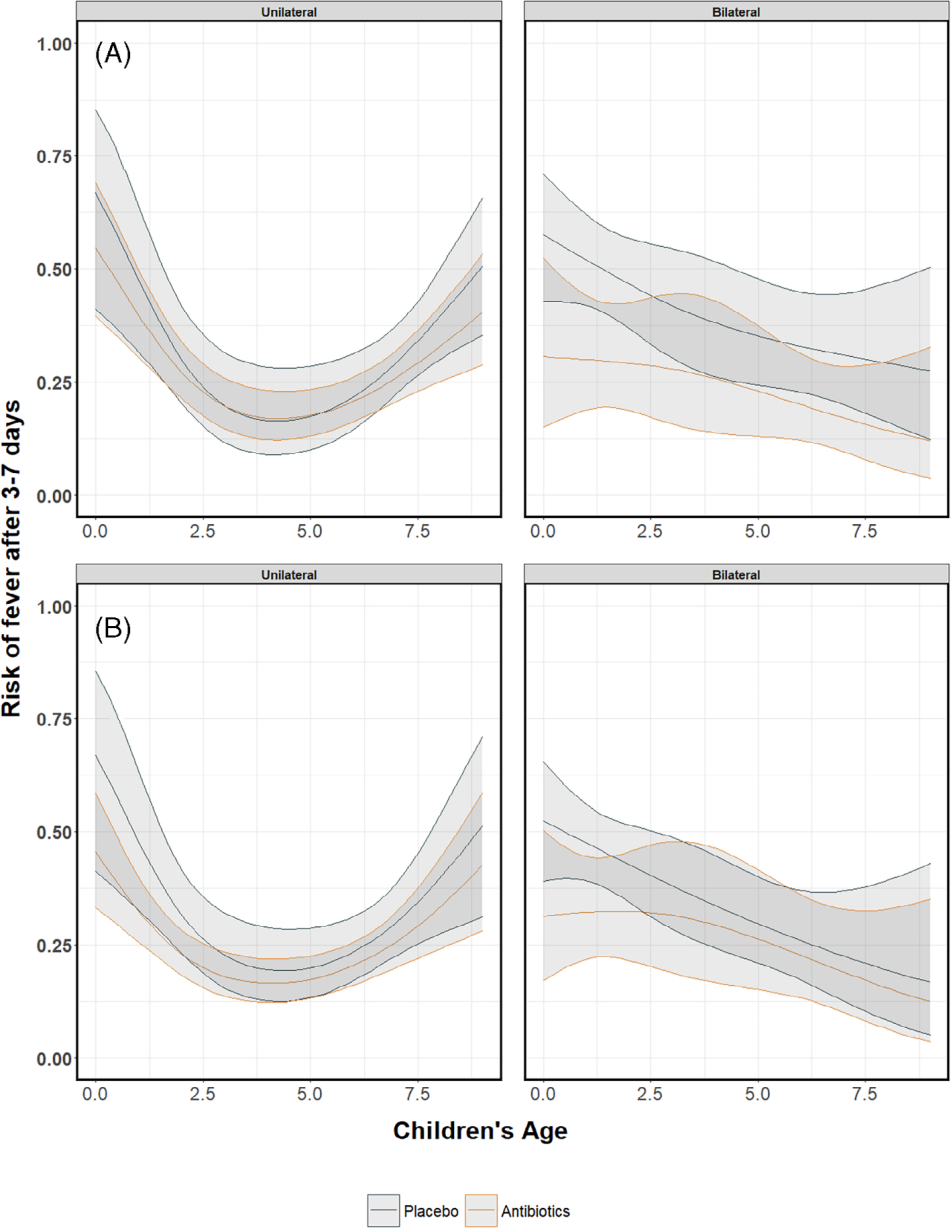
The risk of having fever/ear‐pain after 3–7 days in children with either unilateral or bilateral acute otitis media receiving either antibiotics or placebo. The presented results are estimated using multivariate meta‐analysis with (a) restricted cubic splines and (b) natural B‐splines [Colour figure can be viewed at wileyonlinelibrary.com]

**FIGURE 17:**
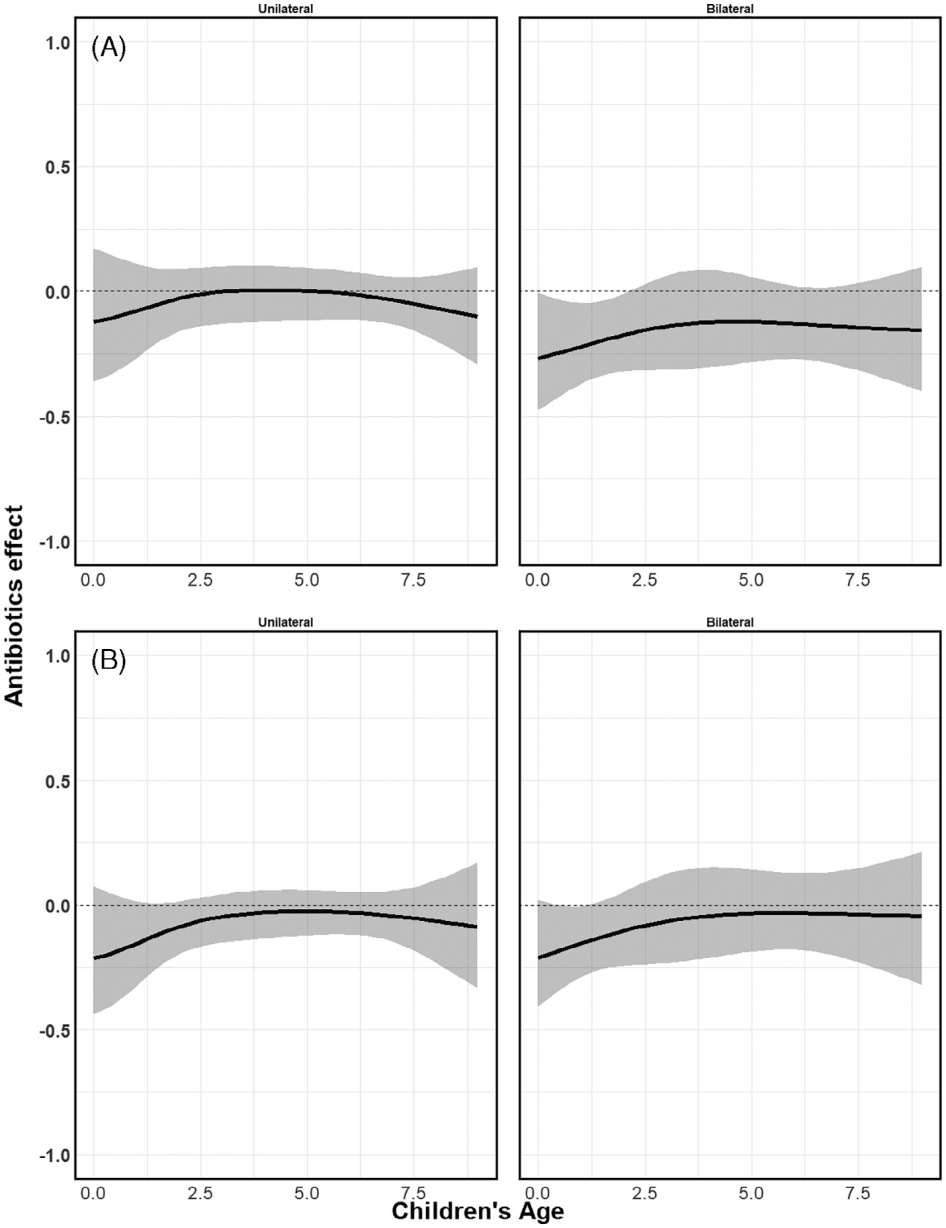
The antibiotics effect (risk difference of having fever/ear‐pain after 3–7 days) in children with either unilateral or bilateral acute otitis media, conditional on age. The presented results are estimated using multivariate meta‐analysis with (a) restricted cubic splines and (b) natural B‐splines

**FIGURE 18:**
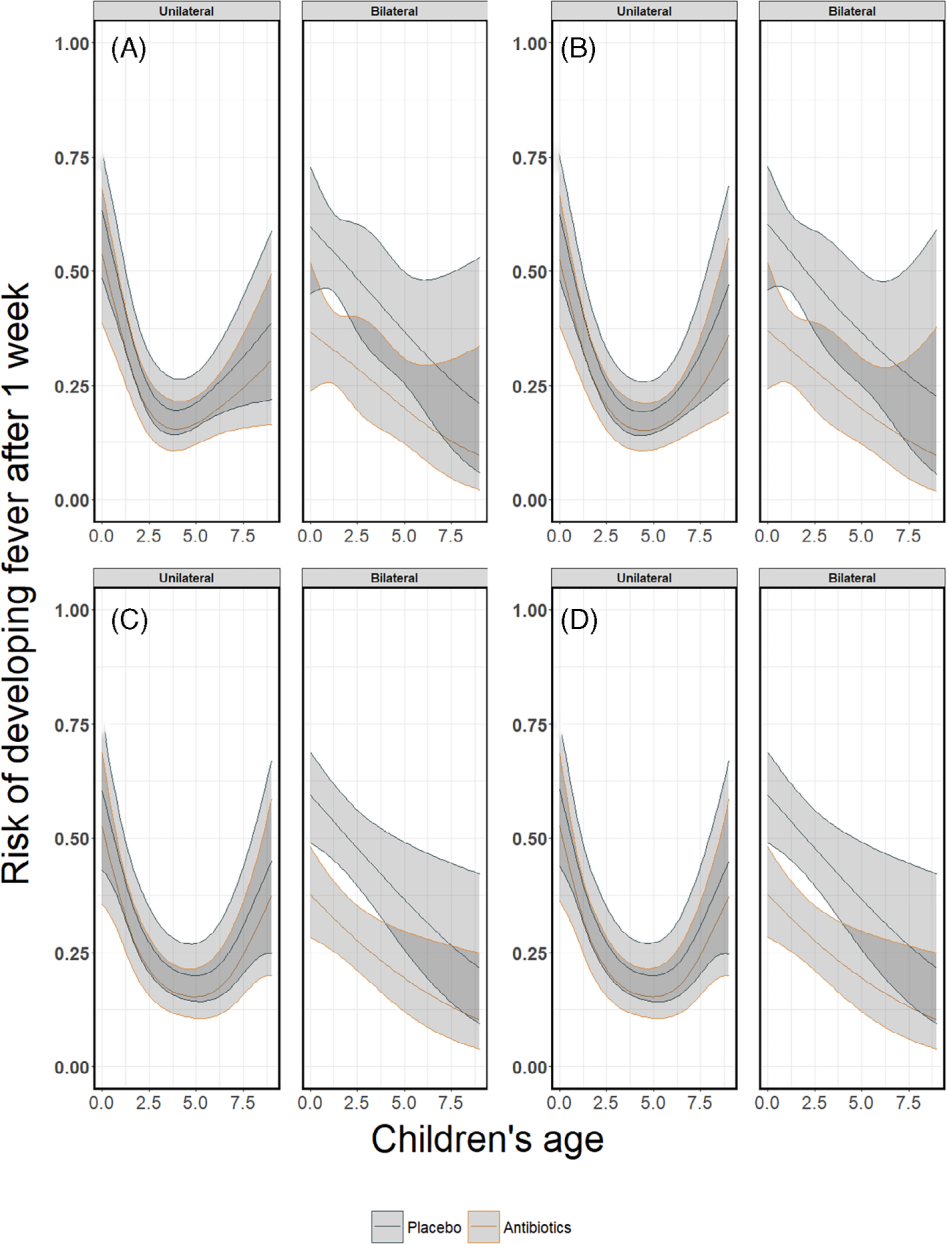
The risk of having fever/ear‐pain after 3–7 days in children with either unilateral or bilateral acute otitis media receiving either antibiotics or placebo. The presented results are estimated using GAMM with (a) restricted cubic splines, (b) natural B‐splines, (c) P‐splines and (d) Smoothing splines [Colour figure can be viewed at wileyonlinelibrary.com]

**FIGURE 19:**
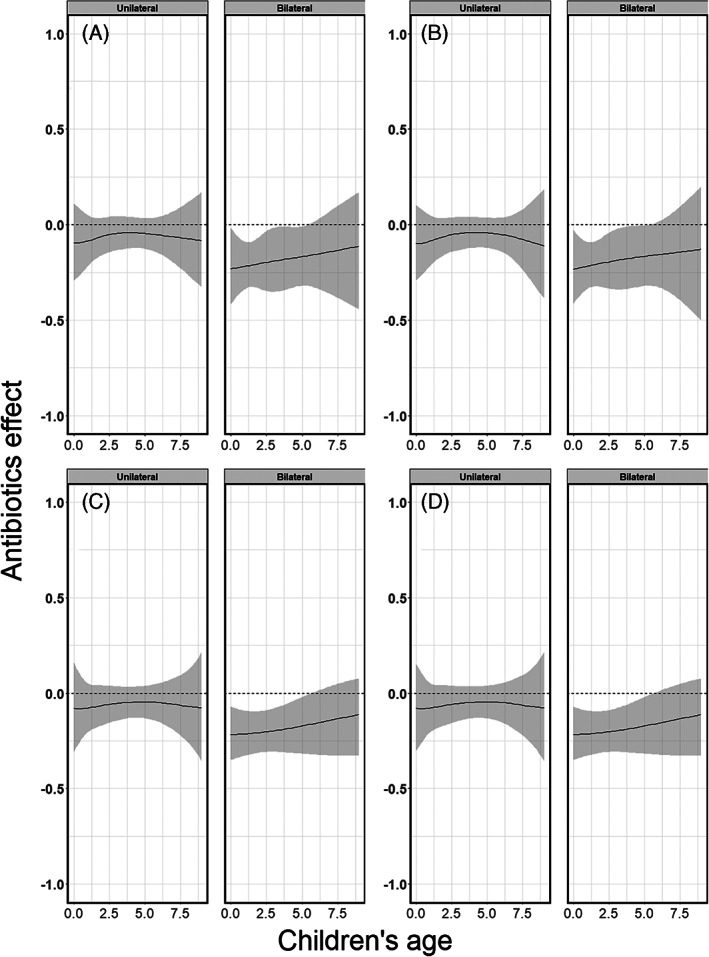
The antibiotics effect (risk difference of having fever/ear‐pain after 3–7 days) in children with either unilateral or bilateral acute otitis media, conditional on age. The presented results are estimated using GAMM with (a) restricted cubic splines, (b) natural B‐splines, (c) P‐splines and (d) Smoothing splines

Figures 14, 16 and 18 show the pooled regression curves of pointwise meta‐analysis, multivariate meta‐analysis and GAMMs, conditional on age and bilaterality of AOM. Figures 15, 17, and 19 show the absolute risk difference between the treated and control group (the treatment effect) conditional on age and bilaterality of AOM. Efficacy of the antibiotics versus the control treatment can be deduced from the confidence intervals for the absolute risk difference by examining whether they exclude the value zero. Similar to the findings in the original report, antibiotics seem efficacious in young children with bilateral AOM.

Since this is an empirical example, the underlying true associations are not known and we cannot draw firm conclusions with respect to the appropriateness of the different approaches. However, we show the pooled curves and compare them with regard to their smoothness and width of confidence intervals, and report convergence issues if any. As we were investigating interactions between treatment, bilaterality of AOM and age, in some studies the combinations of these variables created groups of patients with a limited number of events at certain age ranges. Consequently, pointwise meta‐analysis resulted in wider confidence intervals compared to multivariate meta‐analysis and GAMM. Furthermore, as in the artificial data‐sets, the predicted pooled regression lines were not always smooth due to differences in the age ranges across the studies. Multivariate meta‐analysis resulted in smooth pooled regression curves for both the restricted cubic splines and the natural B splines approach, see Figures 16 and 17. The confidence intervals were also smooth and wider than those of GAMMs. GAMMs resulted in smooth pooled regression lines and narrower confidence intervals than pointwise and multivariate meta‐analysis, see Figures 18 and 19.

## 8 DISCUSSION

Our results, in which we illustrated four spline‐based approaches (restricted cubic splines, natural B‐splines, P‐splines and smoothing splines), and three pooling methods (pointwise meta‐analysis, multivariate meta‐analysis and GAMMs) on three scenarios with artificial data, showed that in case of a heterogeneous data‐set with similar ranges of the effect modifier, all approaches performed equally well in modelling the underlying true association. In the two scenarios with different ranges, pointwise meta‐analysis showed less smooth pooled regression lines and confidence intervals, which were wide in subdomains of BMI. Multivariate meta‐analysis failed to converge for restricted cubic splines and showed results only when combined with natural B‐splines, showing smooth regression lines and confidence intervals. GAMM showed results in both scenarios. GAMM's pooled regression lines and confidence intervals were smooth in all cases. In the empirical example, we investigated the association between age and the effect of antibiotics in children from 0 to 9 years with unilateral or bilateral otitis media. Pointwise meta‐analysis resulted in non‐smooth pooled regression lines and confidence intervals, which were also wider than multivariate meta‐analysis and GAMM. Multivariate meta‐analysis showed smooth pooled regression lines and confidence intervals which were wider than GAMM's. In this specific example, GAMM, especially when combined with penalised splines, resulted in smooth pooled regression lines with narrower confidence intervals than the other pooling methods.

The major strength of our manuscript is that as far as we are aware, we are the first to provide an introduction on how to apply a variety of spline methods in both single and multiple studies, in order to investigate treatment effect differences when non‐linearities are present. In our illustrative examples based on simulated data we introduced three features into our generating mechanisms. First, the association of mortality risk with BMI was simple and realistic as it was based on previously published papers.^29^, ^30^ Second, we generated IPD‐MA scenarios suitable to pool as the between‐study heterogeneity of the regression curves was limited to I^2^ less than 40%. Finally, in the second and third scenarios we generated different boundaries for BMI per study, in order to illustrate the differences between the pooling methods in scenarios where study‐specific domains of X have limited overlap.

Some potential limitations deserve attention. First, we did not illustrate the performance of the aforementioned approaches in a scenario with homogeneous associations and similar ranges of the effect modifier across studies. We considered that this scenario is rarely present in practice and that all approaches would produce similar results. We defined three scenarios, with either none or rather extreme differences in the ranges of the effect modifier across studies, combined with either none or limited between‐study heterogeneity. We chose these settings as they generated data that were appropriate for pooling, and suitable for the purpose of an introductory paper in IPD‐MA, as illustration for the stronger and weaker points of the approaches. We did not investigate the performance of the methods in settings with weaker interaction effects and/or larger between‐study heterogeneity. However, it is reassuring that the findings from our empirical example were similar. Second, we did not illustrate the performance of the pooling methods in scenarios with ecological bias. Modelling choices that avoid ecological bias in presence of non‐linear associations still require further research and were thus outside the scope of this article. Third, we showed the performance of the spline‐based pooling methods in three scenarios and one empirical example. Although results suggest that splines in IPD‐MA provide a helpful tool to evaluate and capture non‐linear treatment effect differences, more research is needed to evaluate their specific strengths and weaknesses. Last, corresponding to our main aim to provide an introduction to splines, we limited our study to spline‐based approaches whereas other techniques might also be able to deal with non‐linear associations, e.g. tree‐based approaches,^7^, ^8^, ^9^, ^10^, ^11^ meta‐STEPP,^12^, ^13^ locally (weighted) estimated scatter‐plot smoothing (lo[w]ess), or fractional polynomials.^14^, ^15^, ^17^

Other researchers have also drawn the attention to the importance of modelling non‐linear associations in IPD‐MA.^25^, ^26^ These studies focused on estimating relative treatment effect functions whereas we focused on estimating the absolute risk differences. Our examples and results show that accounting for non‐linearities is also of great importance if the aim is to investigate treatment effect differences on the absolute scale. Therefore, we believe that this introduction on how to apply splines in IPD‐MA will aid researchers to consider non‐linear relations with a potential effect modifier. Doing so may provide more insight in the underlying associations, contributing to improved evidence synthesis, and ultimately better clinical decision making.

In conclusion, taking non‐linear associations into account whilst combining multiple studies requires careful modelling. Across three IPD‐MA scenarios and one empirical example we showed that pointwise meta‐analysis was flexible and robust to model differences, but non‐smoothness could occur in case of different data domains across studies. Multivariate meta‐analysis was efficient if specified “correctly”, but lacked robustness and flexibility when combined with restricted cubic splines from studies with different domains. GAMM could handle different study domains and sample sizes, whilst producing smooth pooled regression curves, but needs careful modelling. Splines provide a helpful tool to capture non‐linear treatment effect differences in IPD‐MA.

## Supporting information


**Appendix S1**. Supporting Information

## Data Availability

The artificial study data can be reproduced using the formulae in the online Appendix. The data from the empirical example are not publicly available due to privacy and ethical restrictions.
