# Supplementary material for: Predicting personalised absolute treatment effects in individual participant data meta‐analysis: An introduction to splines
Source: Res Synth Methods. 2022 Jan 18;13(2):255–83. doi: 10.1002/jrsm.1546 (PMC9303665; doi:10.1002/jrsm.1546)
Supplement: Supplementary file 1 — Appendix S1. Supporting Information [file JRSM-13-255-s001.zip › JRSM_1546_Online Appendix to splines for IPD-MA.pdf]

Online Appendix for Splines in IPD-MA

Online appendix to: “Predicting personalised absolute treatment effects in individual participant data meta-analysis: an introduction to splines”, by Michail Belias, Maroeska M. Rovers, Jeroen Hoogland, Johannes B. Reitsma, Thomas P.A. Debray, and Joanna IntHout

Reference numbers refer to the reference list in the main manuscript.

Notation

For readability we adopt the following notation throughout the manuscript:

- The trials as j = 1,2, …, n
- Trial participants as i = 1,2, …, n_j,
- Continuous effect modifier: X
- Binary treatment indicator: T with value 0 for the control group and 1 for the experimental group.
- The true association of X with the outcome: f(X)
- Smoothed estimated function: $\widehat{f_s}(X)$
- $[\alpha,\beta]$ the boundaries of X and $[a_j,\beta_j]$ the boundaries of X per trial j
- The number of (inner) knots: κ
- w ϵ [1,…, ϰ +1] the intervals defined by the knots
- g: a link function and g^{-1} its inverse function
- B(X;d) a basis function of dth degree

1. Regression splines

1.1 Truncated power series

In general, to model the association between an independent variable X and an outcome Y, generalised linear models (GLMs) are used. In case of non-linear associations, transformed versions of X can be used instead of X. For instance, the statistical model for a GLM with link function g and with a d-degree polynomial of X is:

$$g(\mu) = \beta_0 + \beta_1 X + \beta_2 X^2 + \cdots + \beta_d X^d \qquad (1)$$

However, a global function over the full range of X may have poor fit near the boundaries due to instability of the estimated polynomial in this area. To avoid these issues, piecewise polynomials may be preferred to global functions. The model for a d-degree polynomial for interval *w*, between knot t_w and t_{w+1}, would be:

$$g(\mu_w) = \beta_{0w} + \beta_{1w} X + \beta_{2w} X^2 + \cdots + \beta_{dw} X^d \qquad (2)$$

These piece-wise polynomials, when fitted in two consecutive intervals, will show different predicted values at the boundaries of the intervals (*i.e.* at the knots), thus their functional shape will be discontinuous. For this reason, we may use restrictions to "connect" interval-specific polynomials. One convenient solution is to fit a global polynomial, and model the deviations from this globally defined shape within truncated parts of X. Thereto, each basis function is a polynomial with one term. Given a non-decreasing sequence of knots $(t_1, t_2, \ldots, t_\kappa)$ a truncated power series basis is defined by the following basis functions:

$$B_0(X) = 1, B_1(X) = X, B_2(X) = X^2, \ldots, B_d(X) = X^d,$$

$$B_{d+1}(X) = (X - t_1)_+^d,\ B_{d+2}(X) = (X - t_2)_+^d, \ldots, B_{d+\kappa}(X) = (X - t_\kappa)_+^d \qquad (3)$$

and the statistical model for the association between X and Y is:

$$g(\mu) = \underbrace{[\beta_0 + \beta_1 X + \beta_2 X^2 + \cdots + \beta_d X^d]}_{basic\ polynomial} + \underbrace{[\beta_{d+1}(X - t_1)_+^d + \beta_{d+2}(X - t_2)_+^d + \cdots + \beta_{d+\kappa} d(X - t_\kappa)_+^d]}_{secondary\ polynomial} \qquad (4)$$

The + subscript denotes that for a given z

$$(z)_+ = \begin{cases} z, & \text{if } z > 0 \\ 0, & \text{otherwise} \end{cases}$$

The first term in equation (4) generates the global polynomial, often called the "basic" polynomial, whereas the second term, often called the "secondary" polynomial, is modelling the deviations from it. The resulting splines, using truncated power series basis functions, are often called polynomial 'regression' splines [60]. The term truncated reflects the fact that the intervals for the power series in the secondary polynomial are shortened to produce estimates only in sub-domains of X. A disadvantage of truncated power series is that they can still show erratic behaviour near the boundaries of X.

1.2 Natural or restricted truncated power series splines

A solution to this erratic behaviour near the boundaries is to restrict the truncated power series to be linear near the boundaries of X [17]. These splines are often called natural or restricted (polynomial) splines. Given a non-decreasing sequence of κ knots $(t_1, t_2, \ldots, t_\kappa)$ the statistical model is given as:

$$g(\mu) = \beta_0 B_0(X) + \beta_1 B_1(X) + \beta_2 B_2(X) + \cdots + \beta_{k-1} B_{k-1}(X) \quad (5)$$

where

$$B_0(X) = 1$$

$$B_1(X) = X$$

and for $w \in [2, \kappa\text{-}1]$

.

$$B_w(X) = (X - t_{w-1})_+^d - \frac{t_k - t_{w-1}}{t_k - t_{k-1}}(X - t_{k-1})_+^d + \frac{t_{k-1} - t_{w-1}}{t_k - t_{k-1}}(X - t_k)_+^d$$

Harrell shows that restricted cubic splines can also be written as truncated power series with a linear "basic" polynomial, by dividing the basis functions by $(t_\kappa - t_1)^{2}$ [37]. Therefore, an equivalent statistical model to (5) may be written as follows:

$$g(\mu) = \underbrace{[\beta_0 + \beta_1 X]}_{basic\ polynomial} + \underbrace{[\beta_2 (X - t_1)_+^3 + \beta_3 (X - t_2)_+^3 + \cdots + \beta_{k+1}(X - t_\kappa)_+^3]}_{secondary\ polynomial} \quad (6)$$

The number and location of the knots may be based on clinical knowledge or on descriptive statistics. For instance, Harrell suggests the use of quantiles and advocates that four knots (at the 5%, 35%, 65%, and 95% quantiles) in most cases are adequate [17, 61]. Depending on the available sample size and required complexity of the functional shape we may use a different number of knots. In Figure 1 (a, b) we show the basis functions of a cubic truncated power series and for the restricted cubic splines approach respectively, with five knots placed at the 5%, 27.5%, 50%, 72.5%, 95% quantiles. In our single study example, we used restricted cubic spline transformations of X both as main effects and as interactions with the treatment. Following Harrell's suggestion, we placed five knots at values corresponding to 5%, 27.5%, 50%, 72.5% and 95% quantiles of X.

1.3 B-splines

B-splines are another commonly applied spline approach. They are based on a parametrisation of polynomial cubic splines. Given a non-decreasing κ knot sequence $t_1, t_2, \ldots, t_\kappa$ and X $\in [\alpha, \beta]$, the dth degree B-splines basis functions are calculated by the following algorithm proposed by De Boor [19].

First, d additional knots are generated before α and d additional knots after β. These are often called outer knots and their choice is arbitrary. We can set them to be equidistant or even equal to the boundary values α and β of X. A new knot sequence $t'_1, t'_2, \ldots t'_d, t'_{d+1}, t'_{d+2}, \ldots, t'_{d+\kappa+1}, t'_{d+\kappa+2}, \ldots . t'_{2d+\kappa}$ is generated, where:

$t'_1, t'_2, \ldots t'_d$ are the left outer knots or endpoints,

$t'_{d+1} = \alpha$,

$t'_{d+2} = t_1, t'_{d+3} = t_2, \ldots, t'_{d+\kappa+1} = t_\kappa$ the inner knots

$t'_{d+\kappa+2} = \beta$,

$t'_{d+\kappa+3}, \ldots . t'_{2d+\kappa+2}$ the right outer knots

Within each interval *w* a zero-degree B-spline is calculated. Zero-degree B-splines are step functions equal to 1 within an interval and 0 otherwise.

$$B_w^0(X) = \begin{cases} 1, & \text{if } t'_w < X < t'_{w+1} \\ 0, & \text{if } t'_w = t'_{w+1} \\ 0, & \text{otherwise} \end{cases}$$

All succeeding basis functions, with degree >1, are calculated using the following formula:

$$B_w^\delta(X) = \left(\frac{X - t_w}{t_{w+\delta} - t_w}\right) B_w^{\delta-1}(X) - \left(\frac{t_{w+\delta+1} - X}{t_{w+\delta+1} - t_{w+1}}\right) B_{w+1}^{\delta-1}(X), \qquad (7)$$

where $\delta \in [1, 2, \ldots, \text{d}]$. For example, the first degree $B_w^1(X) = (\frac{X-t_w}{t_{w+1}-t_w}) B_w^0(X) - (\frac{t_{w+2}-X}{t_{w+2}-t_{w+1}}) B_{w+1}^0(X)$ is calculated using the zero degree B-splines, and the second degree $B_w^2(X)$ from $B_w^1(X)$, and so on, using formula 7.

Three variations of B-splines based on the inner knot positionings have been proposed. B-splines with a uniform knot vector use equidistant knots and are the most typically applied B-splines [24]. B-splines with an open uniform knot vector also use equidistant knots but they allow analysis of closed curves. Non-uniform B-splines use non-equidistant knots, placed at positions of the researcher's choice. To our topic, uniform and non-uniform B-splines are the most relevant. Non-uniform B-splines may reflect the a-priori knowledge of a researcher over the underlying complexity of the functional form and/or distribution of the continuous variable.

1.4 Natural or restricted B-splines

B-splines can also be restricted with the natural property, thus having the second derivative equal to zero at the boundaries. As in truncated power series the natural property reduces the erratic behaviour near the boundaries.

In Figure 1 (c, d) we show the basis functions of a 3rd degree B-spline and a natural 3rd degree B-spline respectively with four equidistant knots; two inner knots plus the boundaries [α, β], placed at values α=0, 0.33, 0.66 and 1=β.

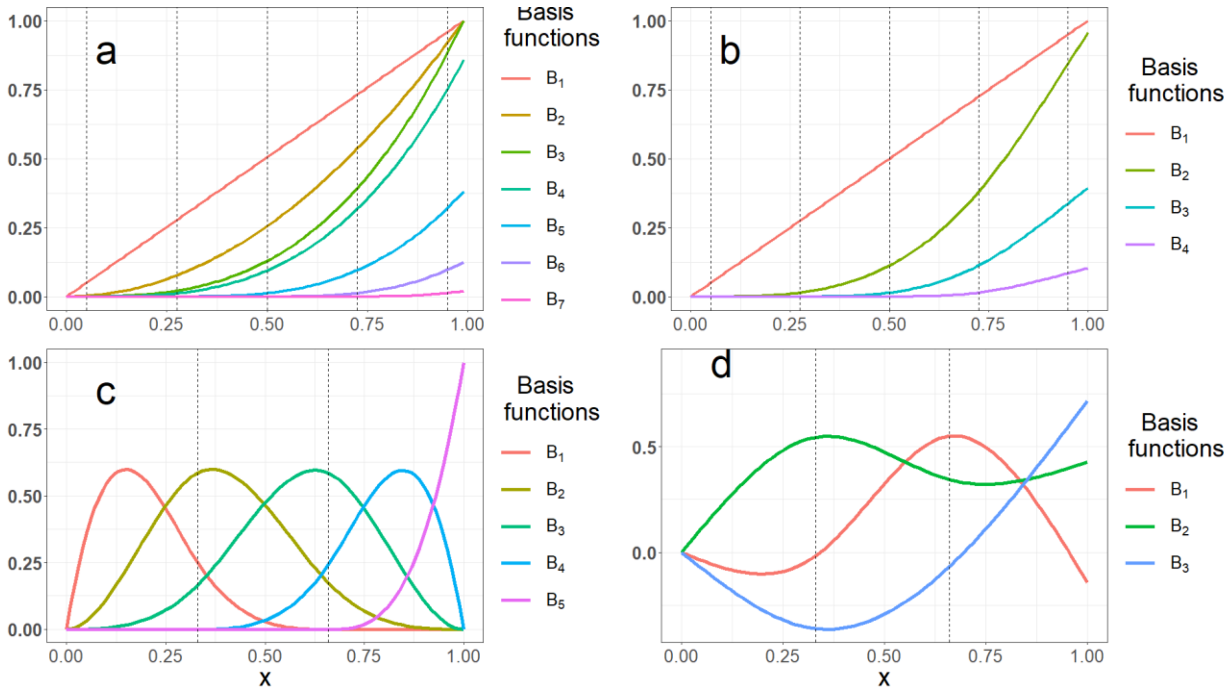

Figure 1. Basis functions of a) cubic truncated power series, 5 knots, b) restricted cubic splines, 5 knots, c) 3rd degree B-spline, 4 knots, d) natural 3rd degree B-spline, 4 knots

2. Penalised splines

The two commonly applied penalised splines that we discuss, P-splines and smoothing splines, increase the number of knots to a large set (usually, 10-40) or even to be equal to the number of observations. This way they circumvent the problem of choosing the number and positions of the knots. Since estimating one parameter for each observation would clearly lead to a perfect fit and thus generate functional shapes with extreme variability, penalised splines introduce in their optimisation functions a penalty term (J_β) multiplied by a non-negative λ, often called a tuning parameter. As the term "tuning" implies, changing the value of λ changes the magnitude of the penalisation.

In GLMs the estimation of the regression coefficients β_i is accomplished through optimisation of functions of β_i. For Gaussian outcomes the least squares optimisation is estimating the $\hat{\beta}_i$ that minimise the squared distance of the predicted and the observed values of the outcome, while for outcomes belonging to the exponential family (Gaussian, Binary, Poisson etc) we estimate the $\hat{\beta}_i$ maximising the likelihood function of β_i. Adding a penalty term (J_β) results in the following optimisation equations:

- Least squares approach

$$\widehat{\beta_\iota} = argmin_\beta \left[\sum_{j=1}^{n} (\hat{Y}_j - \mathrm{Y})^2 + \lambda J_\beta \right]; \; \lambda \geq 0 \quad (9)$$

- Maximum Likelihood approach

$$\widehat{\beta_\iota} = argmax_\beta \left[L(\beta_i; X) - \lambda J_\beta \right]; \;\; \lambda \geq 0 \qquad (10)$$

Penalised splines circumvent the problem of knot selection, but at a cost. By using a penalty in their optimisation function, they introduce bias in their estimate in order to obtain a more stable solution. Further, in both P-splines and smoothing splines the tuning parameter λ must be specified. Too high or too low values of λ may lead to over- or undersmoothing respectively. Several approaches have been proposed in order to determine the "optimal" λ, such as Akaike's information criterion AIC [41], "leave one out" generalised cross-validation (GCV) [42] or mixed-effects modelling [22]. These processes are automated in most of the statistical packages. Briefly, when using the AIC, a series of models fitted with different λ values is compared and the one with the lowest AIC is selected. "Leave one out" GCV is an iterative process, the algorithm goes as follows: 1) one observation is omitted 2) a model is fitted 3) using the model a prediction of the omitted value is generated and 4) the distance between the observed and predicted value is calculated. This procedure is repeated for each observation and for a series of λ_i values. The λ that minimizes the GCV minimizes the sum of the squared distances, i.e. the GCV score, is selected. In Bayesian/mixed effects modelling approach the penalty term is estimated in a similar way as random effects parameters.

2.1 P-splines

A specific type of penalised splines, P-splines, proposed by Eilers and Marx [21], is a penalised version of B-splines, using a specific penalty term based on the sum of p-order differences between the coefficients of two consecutive intervals $J_\beta = \sum(\Delta^p \beta_w)^2$. The first order differences are defined as follows:

$\Delta(\beta_w) = (\beta_w - \beta_{w-1})$, but Eilers and Marx propose the use of second order differences, which are the first order differences of the first order differences

$$\Delta^2(\beta_w) = \Delta(\Delta(\beta_w)) = \Delta(\beta_w) - \Delta(\beta_{w-1}) = [\beta_w - \beta_{w-1}] - [\beta_{w-1} - \beta_{w-2}].$$

Note that the degree of the underlying B-splines may be different from the order of the differences. A common combination is that of a third-degree B-spline with a second order difference. Using a penalty based on a zero-degree order difference $J_\beta = \sum_{w=3}^{K+4}(\beta_w)^2$ results in the ridge penalty [43]. Note that in some occasions penalised splines and penalised B-splines are misinterpreted as P-splines, but not all penalised B-splines or penalised splines are P-splines. For instance, ordinary B-splines may be fitted using a smoothing splines approach, but this does not make them P-splines, unless they are penalised using the approach suggested by Eilers and Marx.

P-splines are based on equidistant knots. It is possible to use a knot sequence that is not evenly spaced; but in this case, weights need to be introduced [22, 24]. As P-splines with non-equidistant knots are rarely used in practice we don't consider them in this article.

2.2 Smoothing splines

Smoothing splines are another member of the family of penalised spline methods. Similar to P-splines the idea is to increase the number of knots, but this time to be equal or approximately equal to the number of observations. O' Sullivan [41] suggested that a penalty based on Reinsch's integral of the second derivative of $f(X)$, where $f(X)$ is a cubic spline, multiplied by a tuning parameter, has good smoothing properties. This results in the following penalty term for smoothing splines: $J_\beta = \int_a^b (f''(X))^2\, dx)$.

3. Formulas used to generate the artificial data for the single study

The risk of mortality per participant i in the single study data-set was generated using the following formulas:

- For the control group

$$f_C(BMI_i) \;=\; 0.2 + (\frac{BMI_i - 25}{20})^2 \qquad (1)$$

- For the treated group

$$f_T(BMI_i) \;= 0.2 + (\frac{BMI_i - 25}{20})^4 \qquad (2)$$

Equivalently equations (1) and (2) can also be combined into a single equation:

$$\mathrm{f}(BMI_i) = 0.2 + \underbrace{(\frac{BMI_i - 25}{20})^2}_{f_C(X)} + 0 \times T_i + \underbrace{(\frac{BMI_i - 25}{20})^4 \times T_i - (\frac{BMI_i - 25}{20})^2 \times T_i}_{f_{int}(X)}$$

where $f_C(X)$ would be the association of BMI with mortality risk for the control and $f_{int}(X)$ the additive effect of the treatment.

4. Formulas used to generate the artificial data for the studies in scenarios 1 to 3

The risk of mortality per participant i and per study j in the three scenarios with 5 studies each was generated using the following formulas:

	**TRUE UNDERLYING FUNCTION FORMS**		**BMI RANGES**				
**SCENARIOS**	Control	Treated	1st Study	2nd Study	3rd Study	4th Study	5th Study
**HETEROGE-NEOUS DATA-SET WITH EQUAL BMI RANGES**	$0.2 + (\frac{BMI_{ij} - 25}{20})^2 + d_{1j}$ $d_{1j} \sim Unif(-0.05,0.05)$	$0.2 + (\frac{BMI_{ij} - 25}{20})^4 + d_{2j}$ $d_{2j} \sim Unif(-0.05,0.05)$	[18.5,40]	[18.5,40]	[18.5,40]	[18.5,40]	[18.5,40]
**NON-HETERO-GENEOUS DATA-SET WITH DIFFERENT BMI RANGES**	$0.2 + (\frac{BMI_{ij} - 25}{20})^2$	$0.2 + (\frac{BMI_{ij} - 25}{20})^4$	[18.5,27]	[21.2,30.2]	[24.5,33.5]	[27.8,36.7]	[31.40]
**COMBINED DATA-SET WITH DIFFERENT BMI RANGES AND BETWEEN STUDY DIFFERENCES IN THE MORTALITY RISKS**	$0.2 + (\frac{BMI_{ij} - 25}{20})^2 + d_{1j}$ $d_{1j} \sim Unif(-0.05,0.05)$	$0.2 + (\frac{BMI_{ij} - 25}{20})^4 + d_{2j}$ $d_{2j} \sim Unif(-0.05,0.05)$	[18.5,27]	[21.2,30.2]	[24.5,33.5]	[27.8,36.7]	[31.40]

5. Additional information on splines and pooling methods on the data from scenario 3 (data with different BMI ranges and between-study differences in the mortality risks)

5.1 Spline predictions per study for the four spline approaches

On the following pages you can find for each spline approach two figures.

First, per study and spline approach the predicted spline including 95% confidence interval on logit scale is presented. The underlying observed (0/1) mortality data are presented on logit scale by means of a logistic regression model per study.

The results on logit scale are used for the pooling, either by pooling the predicted curves in pointwise meta-analysis, or by pooling the coefficients in the multivariate meta-analysis.

Second, the corresponding fit of the spline approaches on the mortality risk scale is presented. In these figures, the pattern in the observed (0/1) mortality data is presented by means of loess plots.

Restricted cubic splines

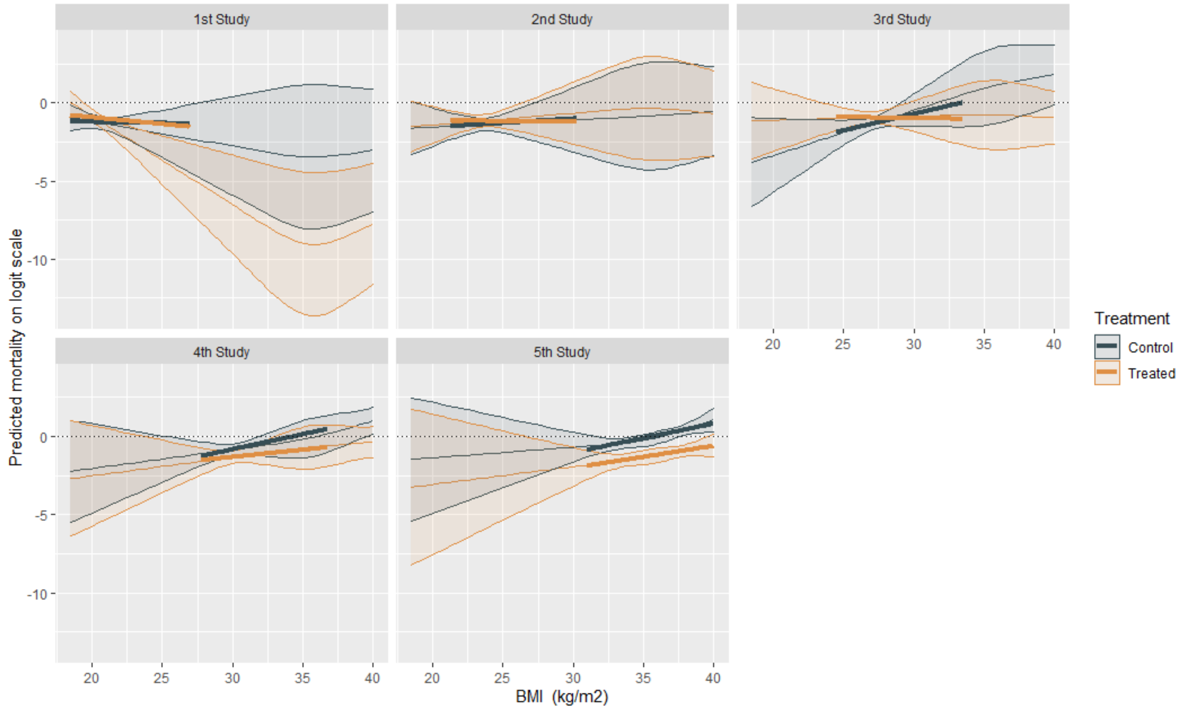

Figure 5.1.A Logit mortality as predicted by restricted cubic splines including 95% CI, combined with fitted (logit) lines from logistic regression on observed (0/1) mortality data per study

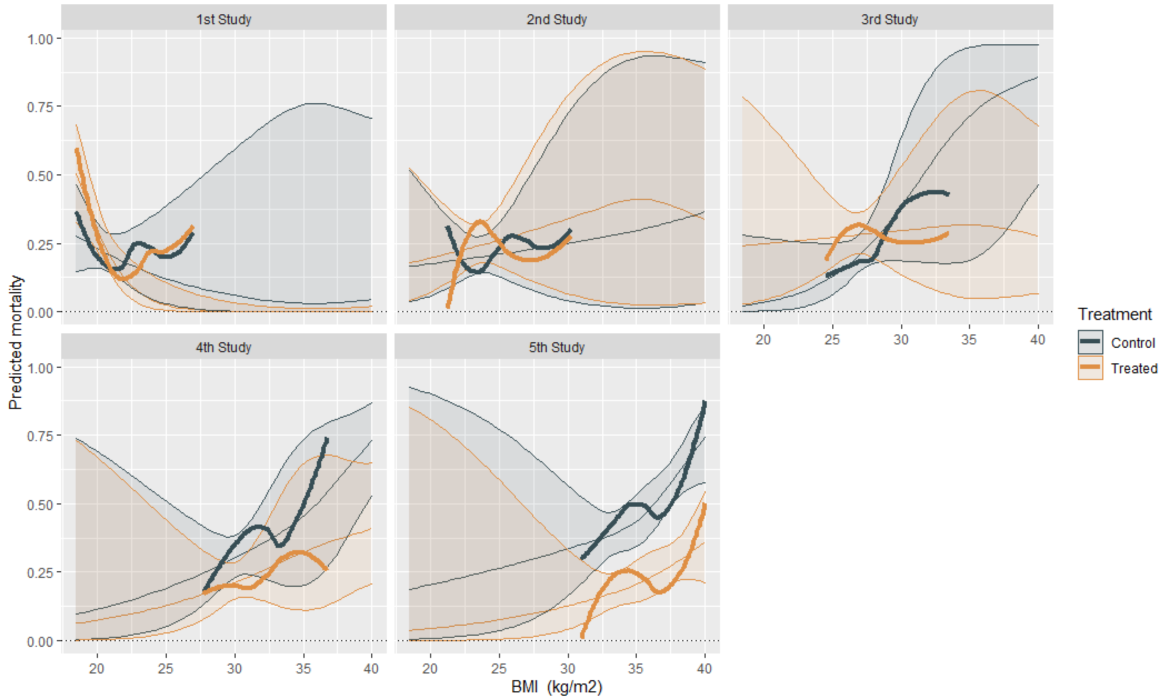

Figure 5.1.B Mortality as predicted by restricted cubic splines including 95% CI, combined with loess plots of the observed (0/1) mortality data per study

Natural B-splines

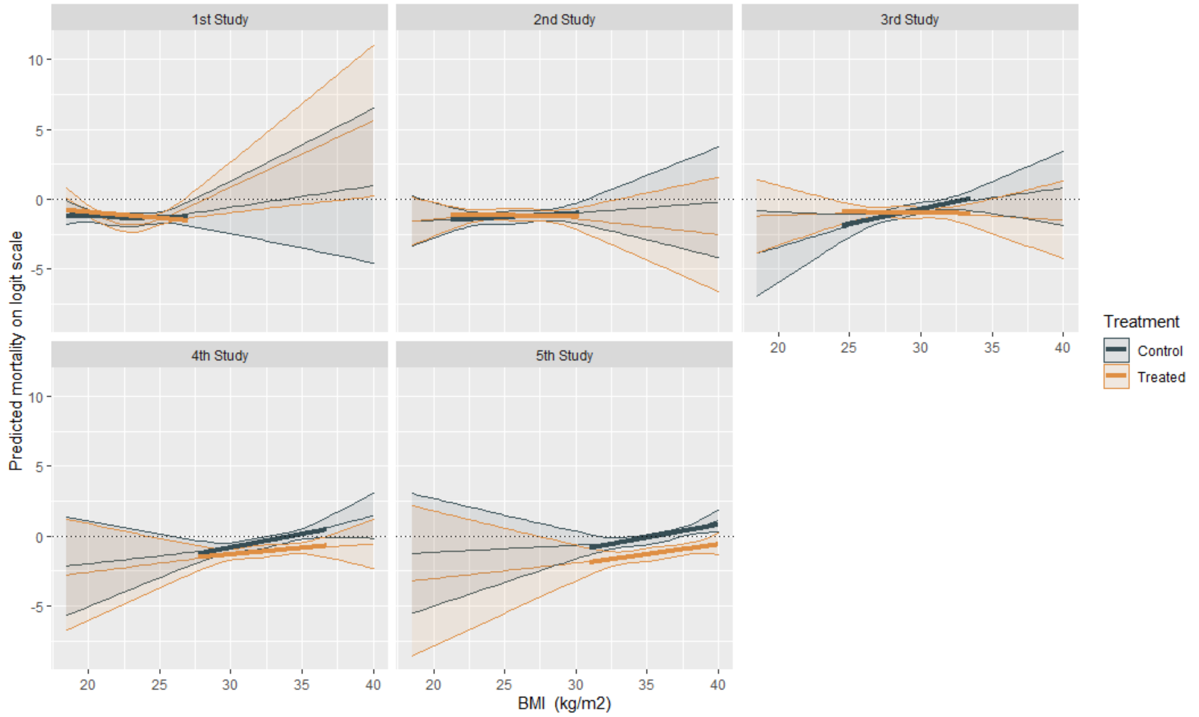

Figure 5.1.C Logit mortality as predicted by natural B-splines including 95% CI, combined with fitted (logit) lines from logistic regression on observed (0/1) mortality data per study

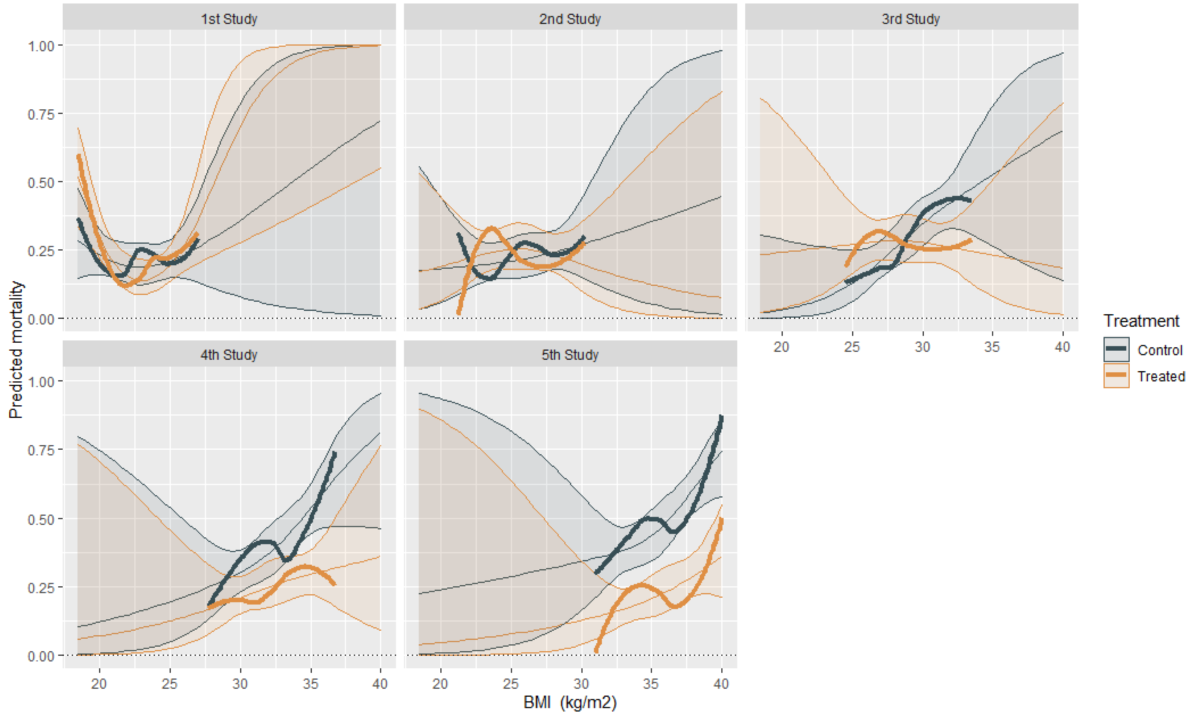

Figure 5.1.D Mortality as predicted by natural B-splines including 95% CI, and by loess plots of the observed (0/1) mortality data per study

P-splines

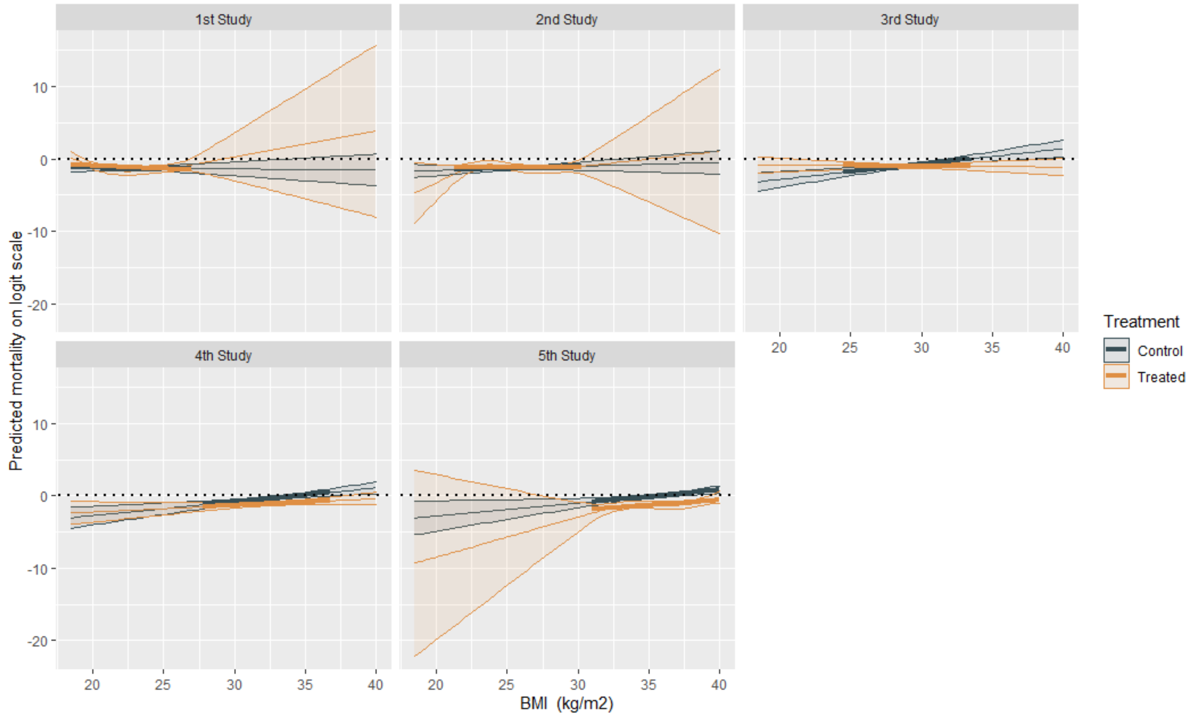

Figure 5.1.E Logit mortality as predicted by P-splines including 95% CI, combined with fitted (logit) lines from logistic regression on observed (0/1) mortality data per study

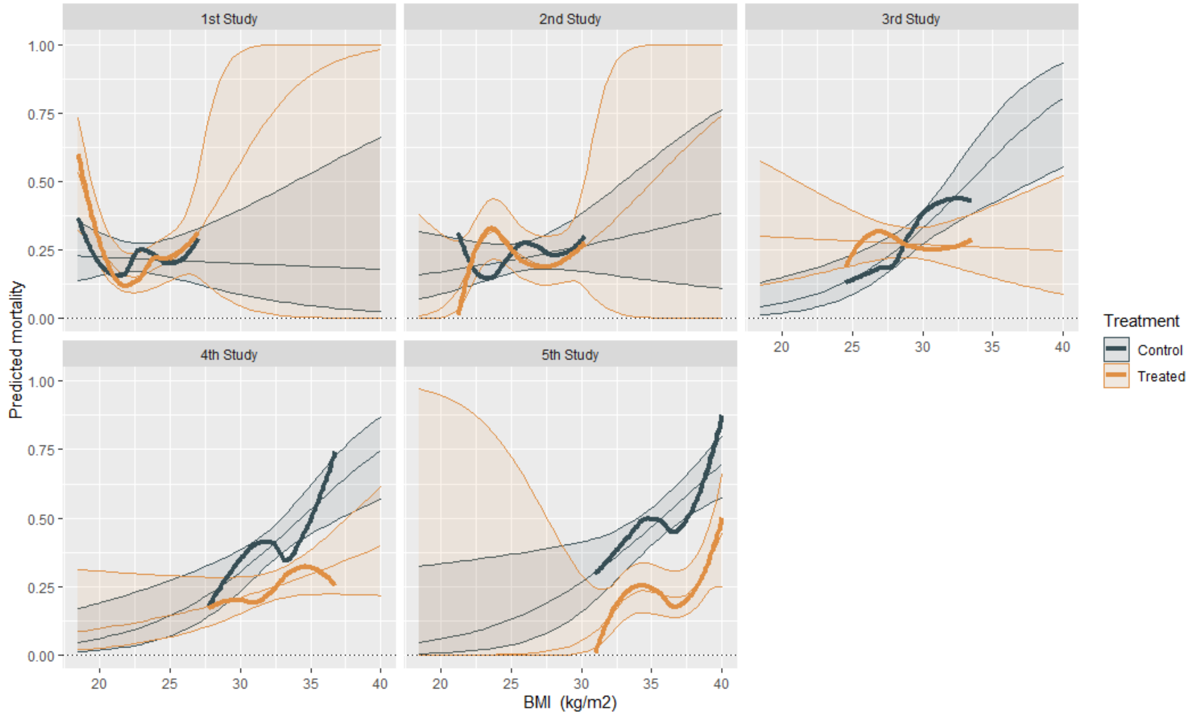

Figure 5.1.F Mortality as predicted by P-splines including 95% CI, and by loess plots of the observed (0/1) mortality data per study

Smoothing splines

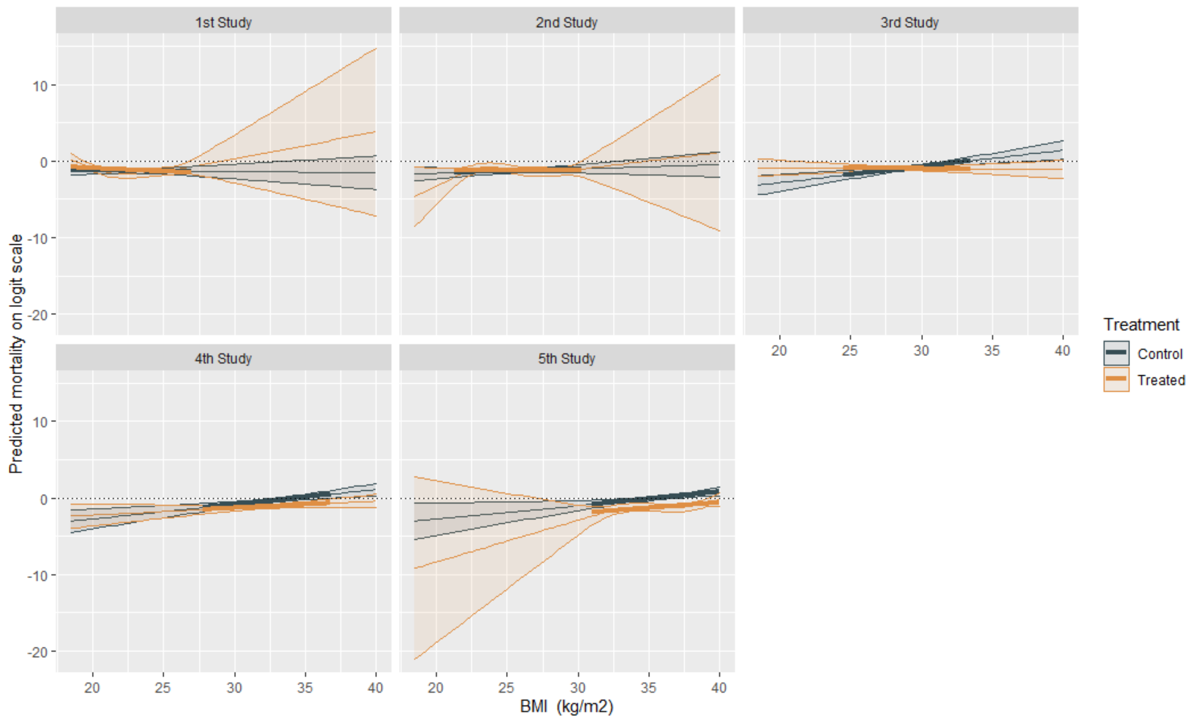

Figure 5.1.G Logit mortality as predicted by Smoothing splines including 95% CI, combined with fitted (logit) lines from logistic regression on observed (0/1) mortality data per study

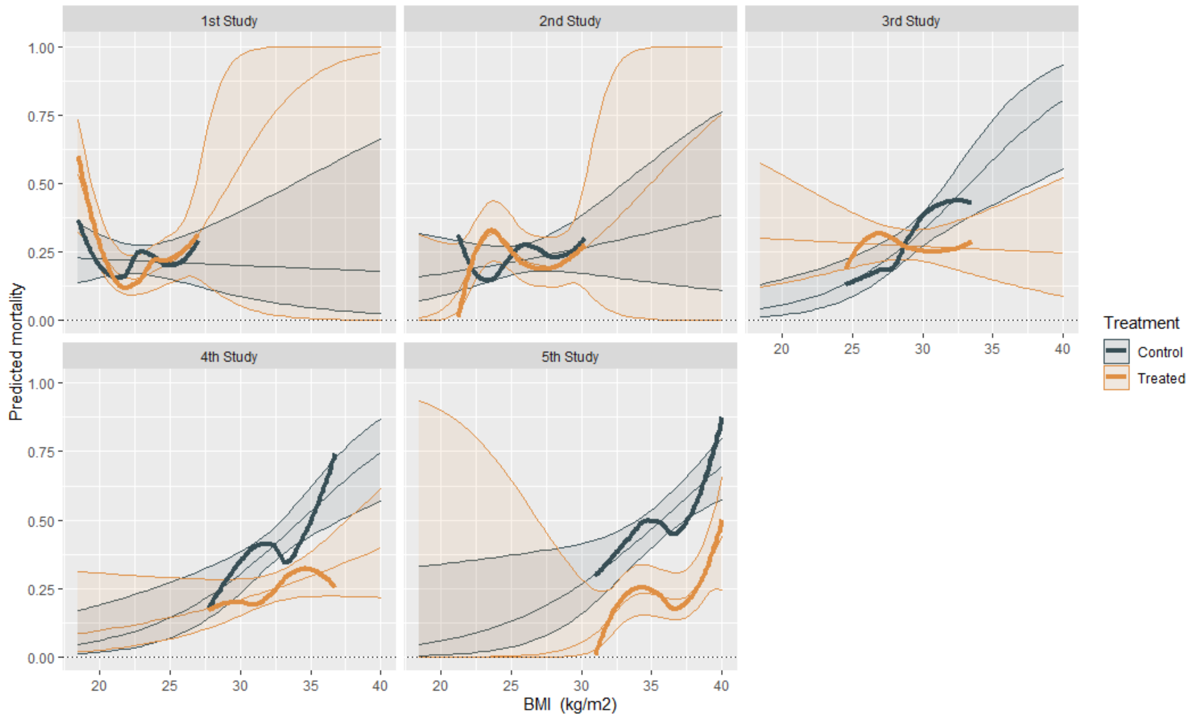

Figure 5.1.H Mortality as predicted by Smoothing splines including 95% CI, and by loess plots of the observed (0/1) mortality data per study

5.2 Pointwise meta-analysis in combination with restricted cubic splines

First we show the estimated heterogeneity (tau) across the BMI range (Figure **A**). In combination with the standard errors of the fitted spline curves per study (see section 5.1 in this online appendix), this determines the weights of the studies in the random-effects pointwise meta-analysis.

Next, we present the pooled curves, both on logit and on mortality risk scale (figures **B** and **C**, respectively).

Finally, we show how the individual study predicted spline curves correspond to the pooled predicted curve, both on logit scale (per treatment, figures **D** and **E**), and on the mortality risk scale using loess plots (Figure **F**).

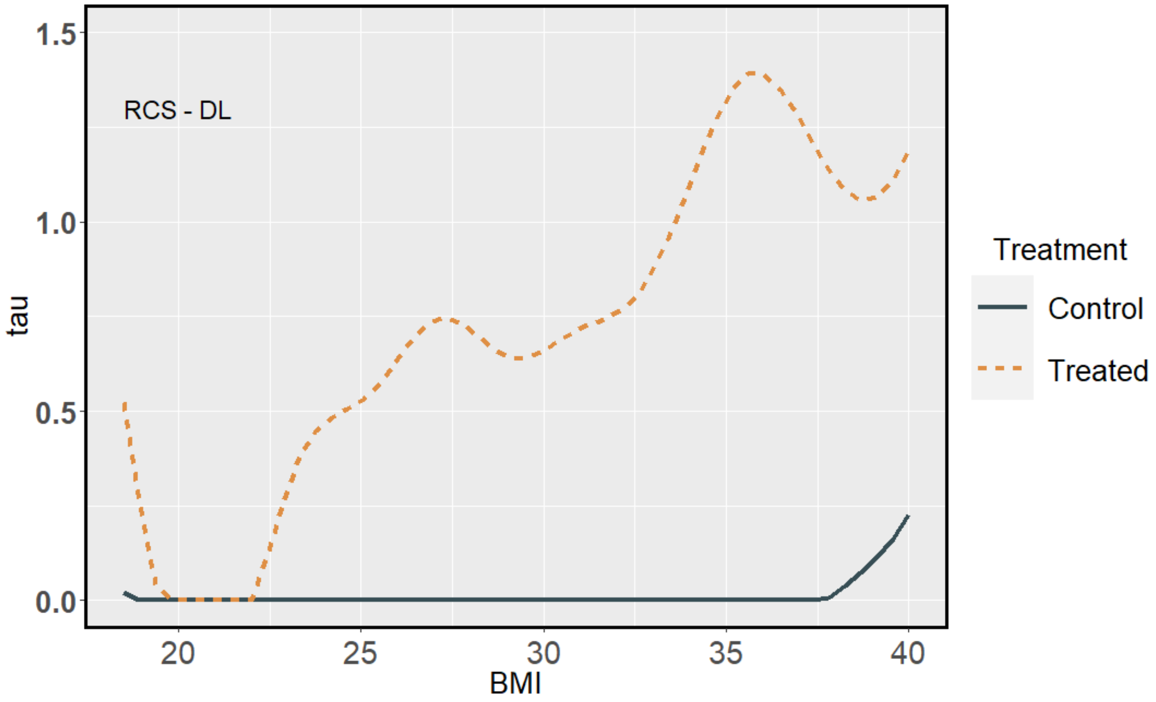

Figure 5.2.A Heterogeneity as estimated by tau (method of DerSimonian Laird, as REML did not converge for some of the BMI values), resulting from pointwise meta-analysis with restricted cubic splines

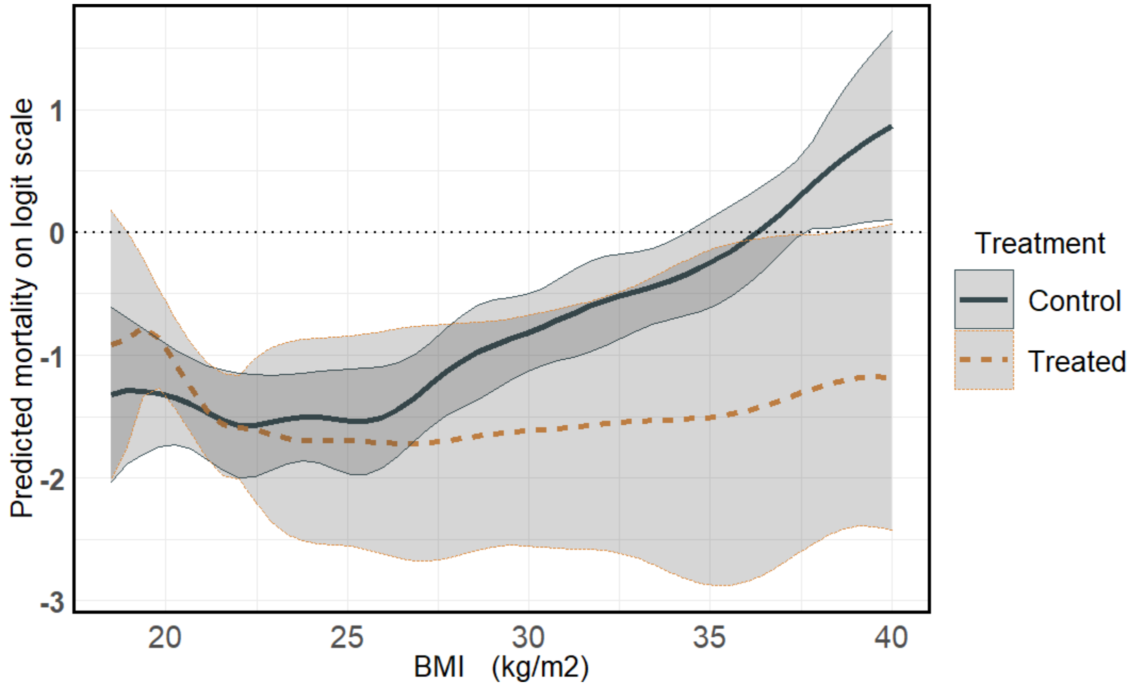

Figure 5.2.B Pooled predicted mortality on logit scale with 95% CI, resulting from pointwise meta-analysis with restricted cubic splines

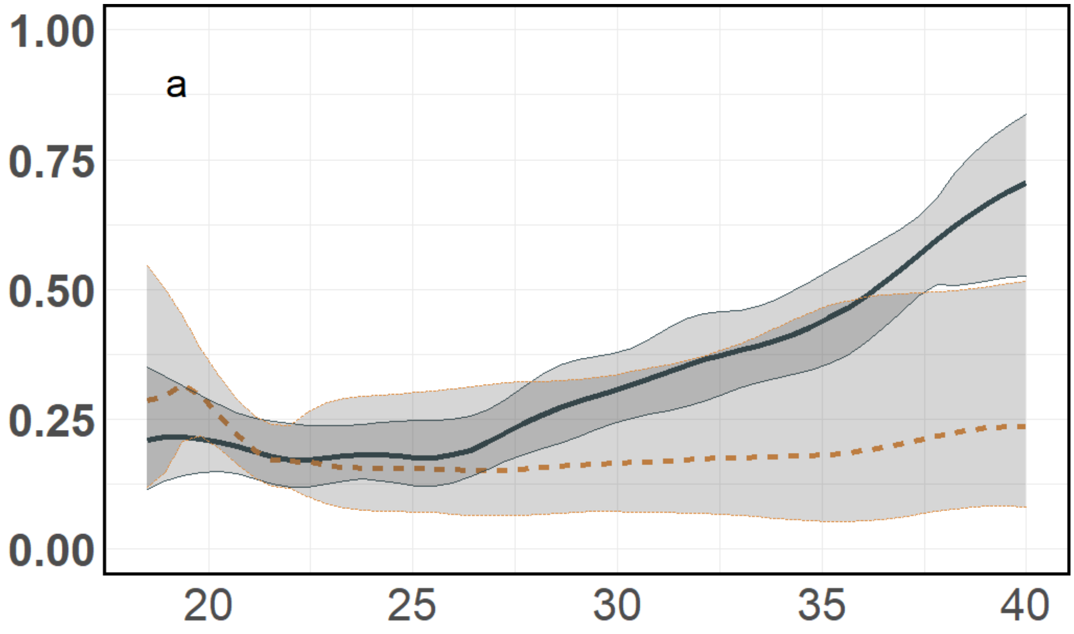

Figure 5.2.C Pooled predicted mortality risk with 95% CI, resulting from pointwise meta-analysis with restricted cubic splines
(Figure from main manuscript)

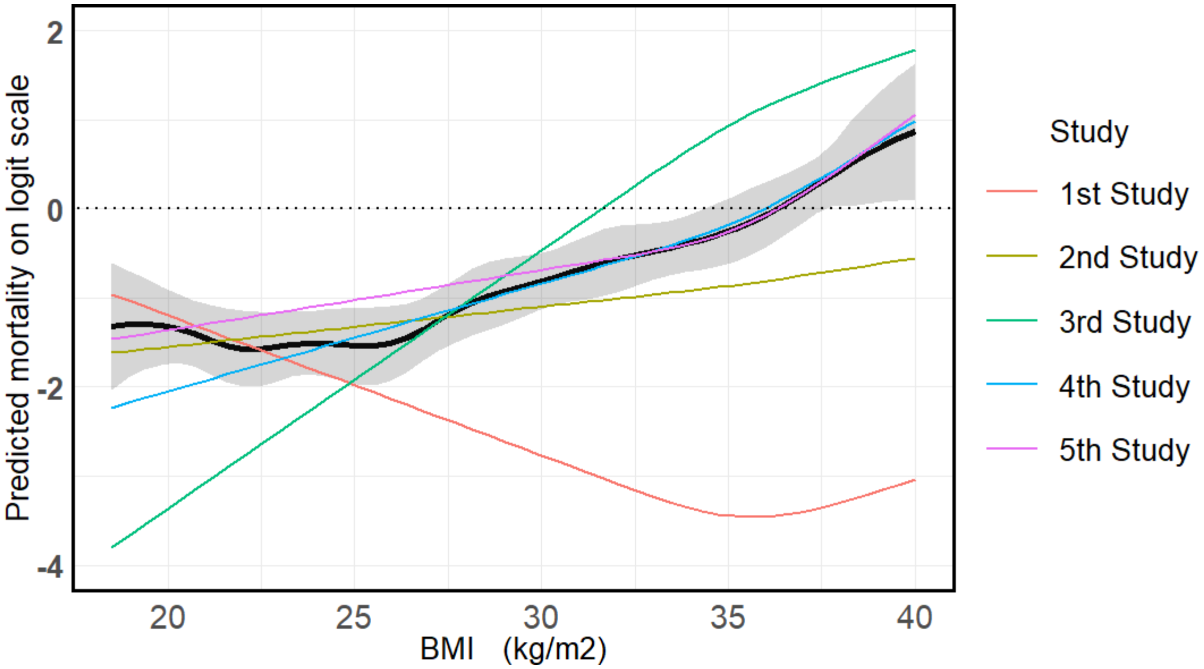

Figure 5.2.D Control group: pooled predicted mortality on logit scale with 95% CI, resulting from pointwise meta-analysis with restricted cubic splines, and restricted cubic splines per study

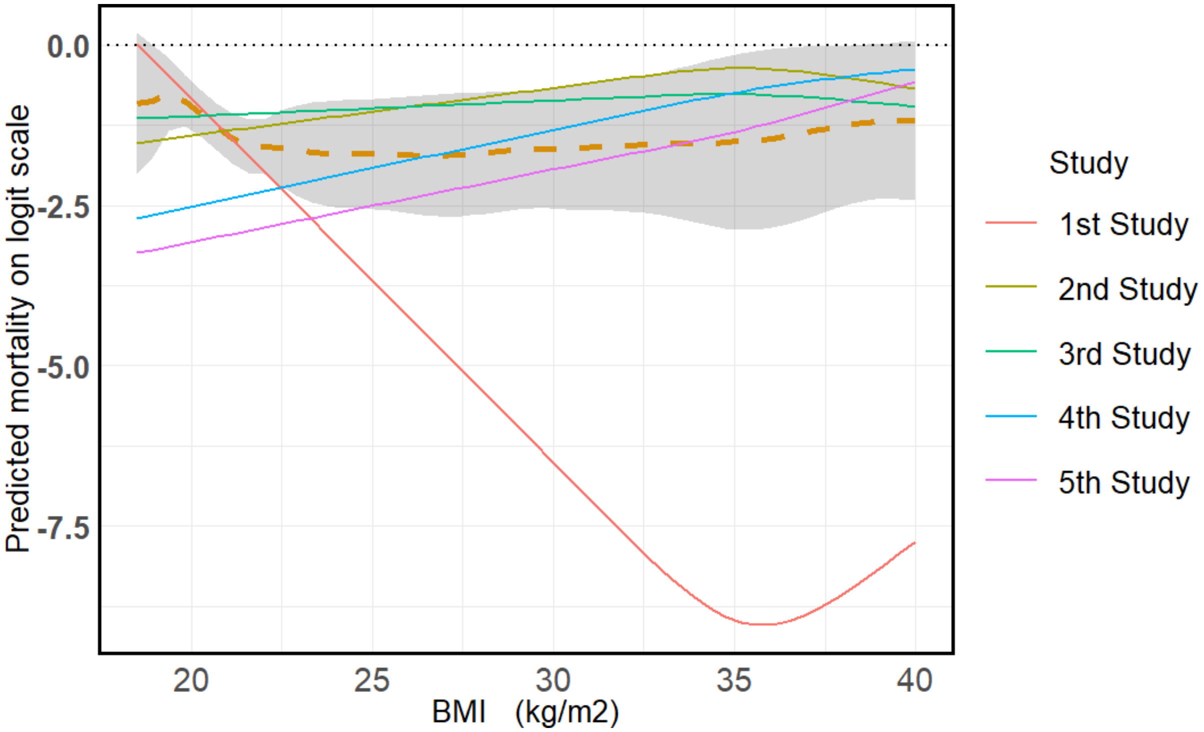

Figure 5.2.E Treated group: pooled predicted mortality on logit scale with 95% CI, resulting from pointwise meta-analysis with restricted cubic splines, and restricted cubic splines per study

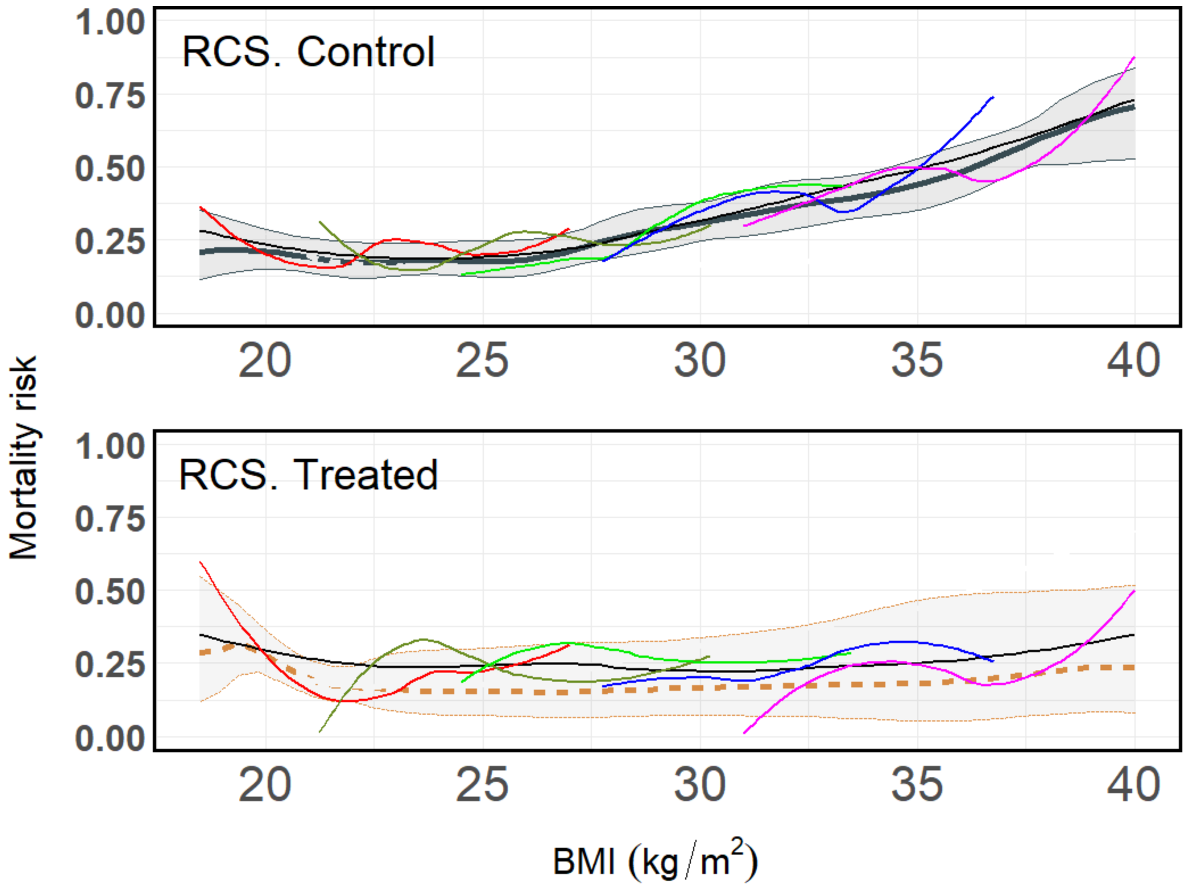

Figure 5.1.F Pooled predicted mortality risk with 95% CI, resulting from pointwise meta-analysis with restricted cubic splines, including loess plots of the observed mortality data per study

5.3 Pointwise meta-analysis in combination with natural B-splines

First we show the estimated heterogeneity (tau) across the BMI range (Figure **A**). In combination with the standard errors of the fitted spline curves per study (see section 5.1 in this online appendix), this determines the weights of the studies in the random-effects pointwise meta-analysis.

Next, we present the pooled curves, both on logit and on mortality risk scale (figures **B** and **C**, respectively).

Finally, we show how the individual study predicted spline curves correspond to the pooled predicted curve, both on logit scale (per treatment, figures **D** and **E**), and on the mortality risk scale using loess plots (Figure **F**).

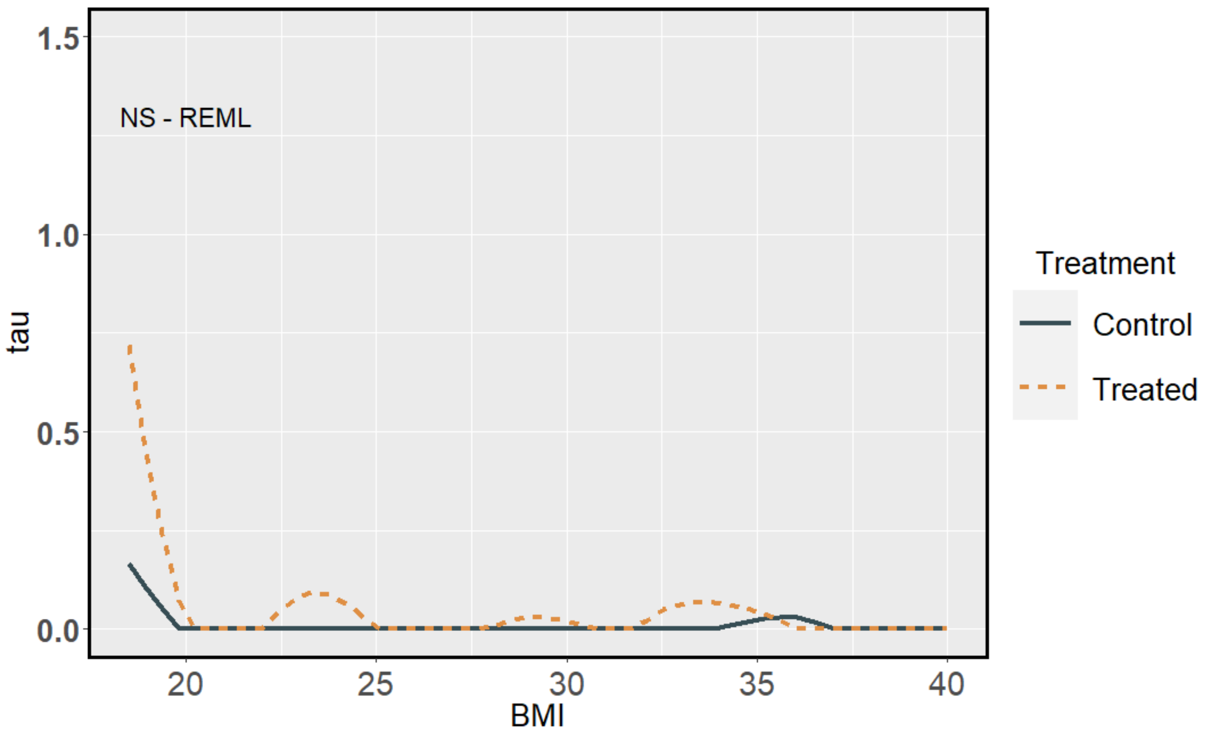

Figure 5.3.A Heterogeneity as estimated by tau, resulting from pointwise meta-analysis with natural B-splines

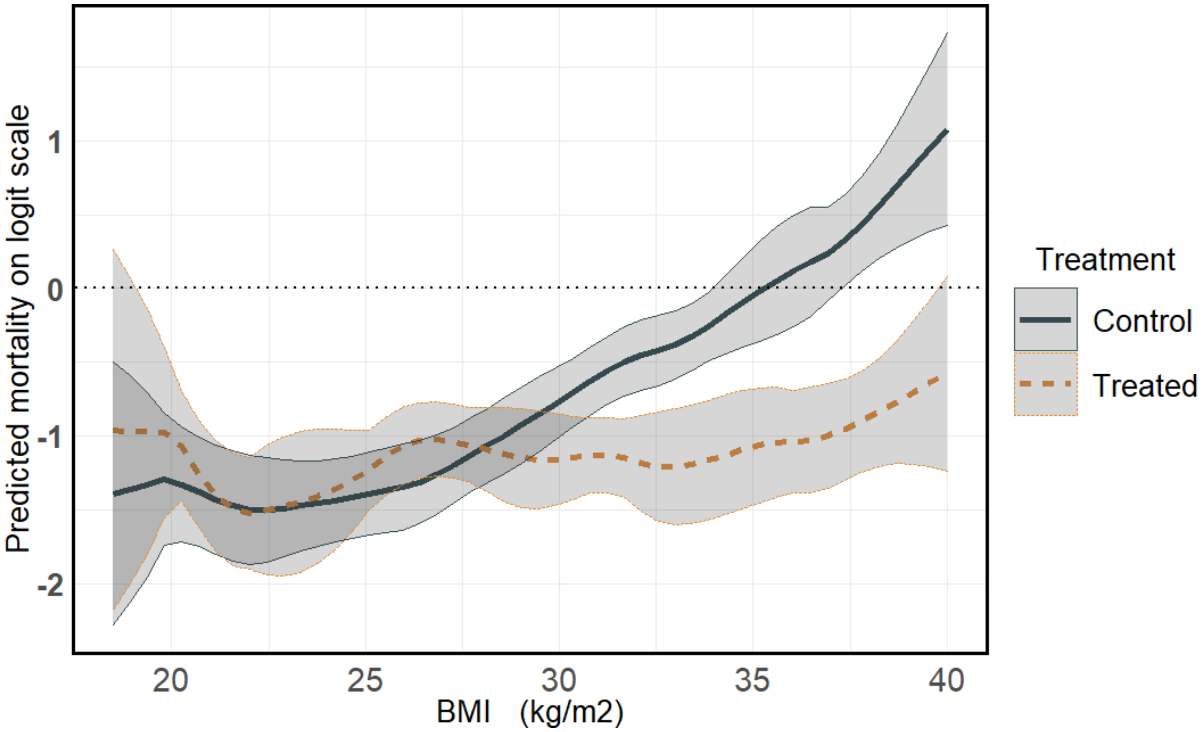

Figure 5.3.B Pooled predicted mortality on logit scale with 95% CI, resulting from pointwise meta-analysis with natural B-splines

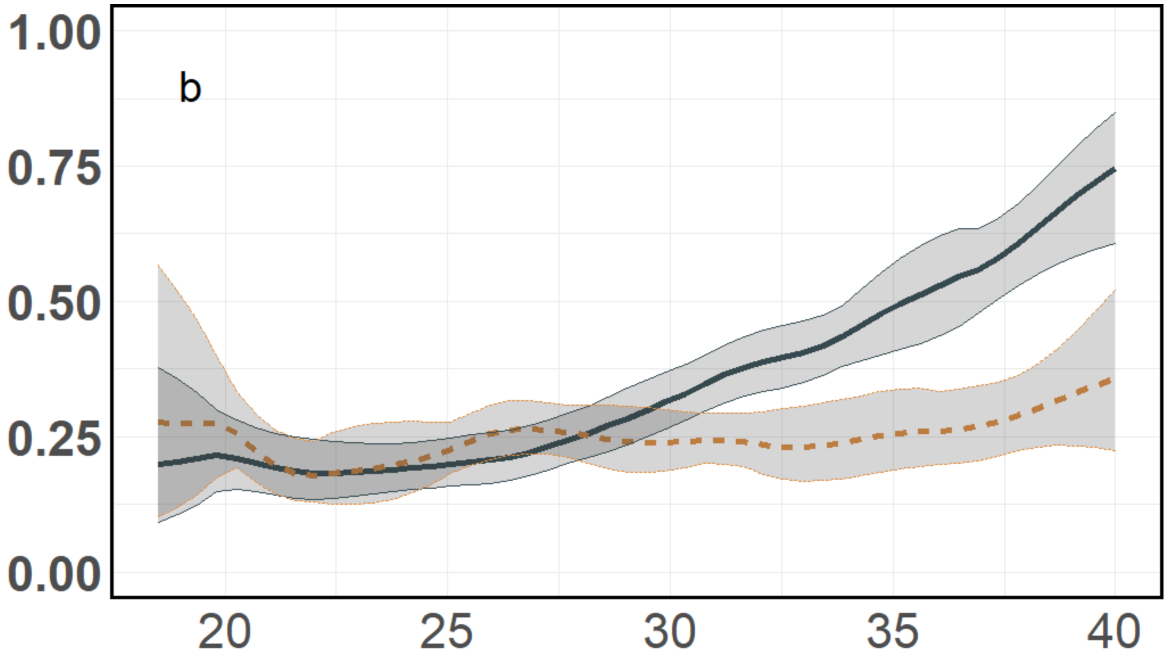

Figure 5.3.C Pooled predicted mortality risk with 95% CI, resulting from pointwise meta-analysis with natural B-splines
(Figure from main manuscript)

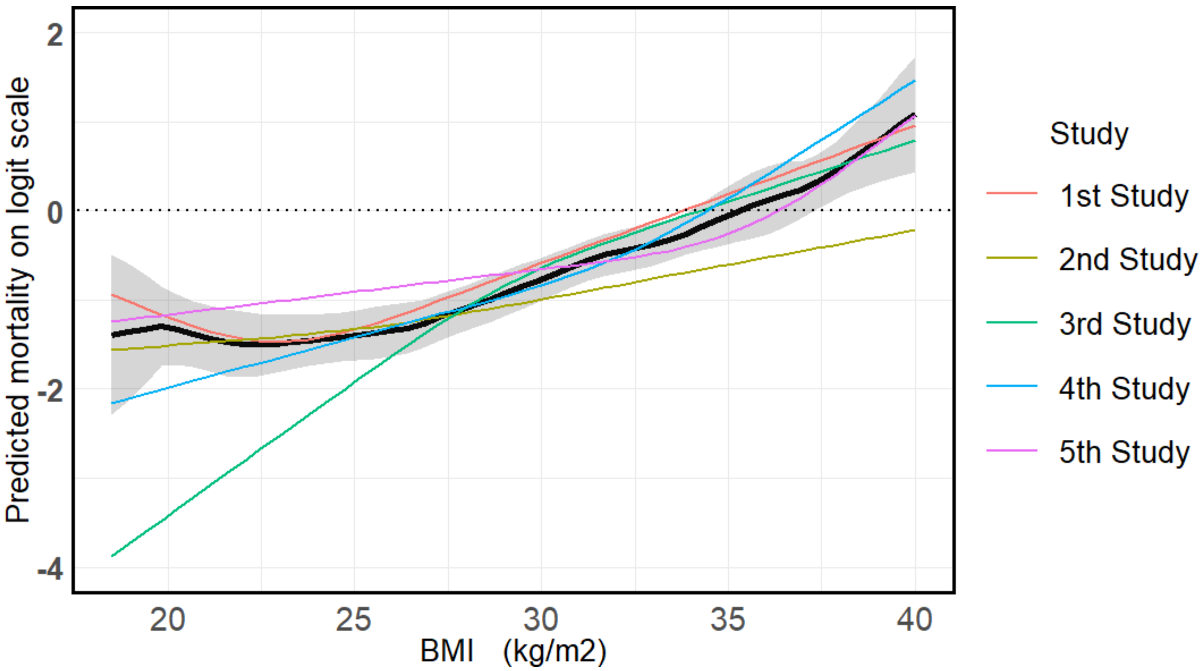

Figure 5.3.D Control group: pooled predicted mortality on logit scale with 95% CI, resulting from pointwise meta-analysis with natural B-splines, and natural B-splines per study

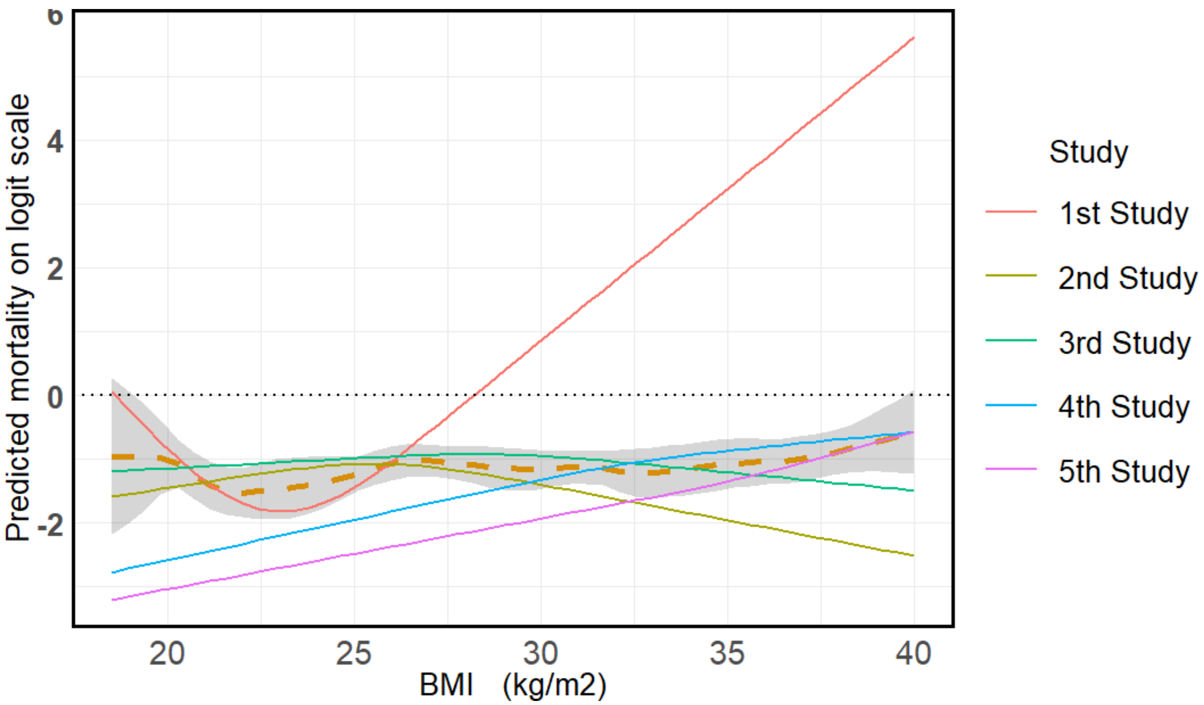

Figure 5.3.E Treated group: pooled predicted mortality on logit scale with 95% CI, resulting from pointwise meta-analysis with natural B-splines, and natural B-splines per study

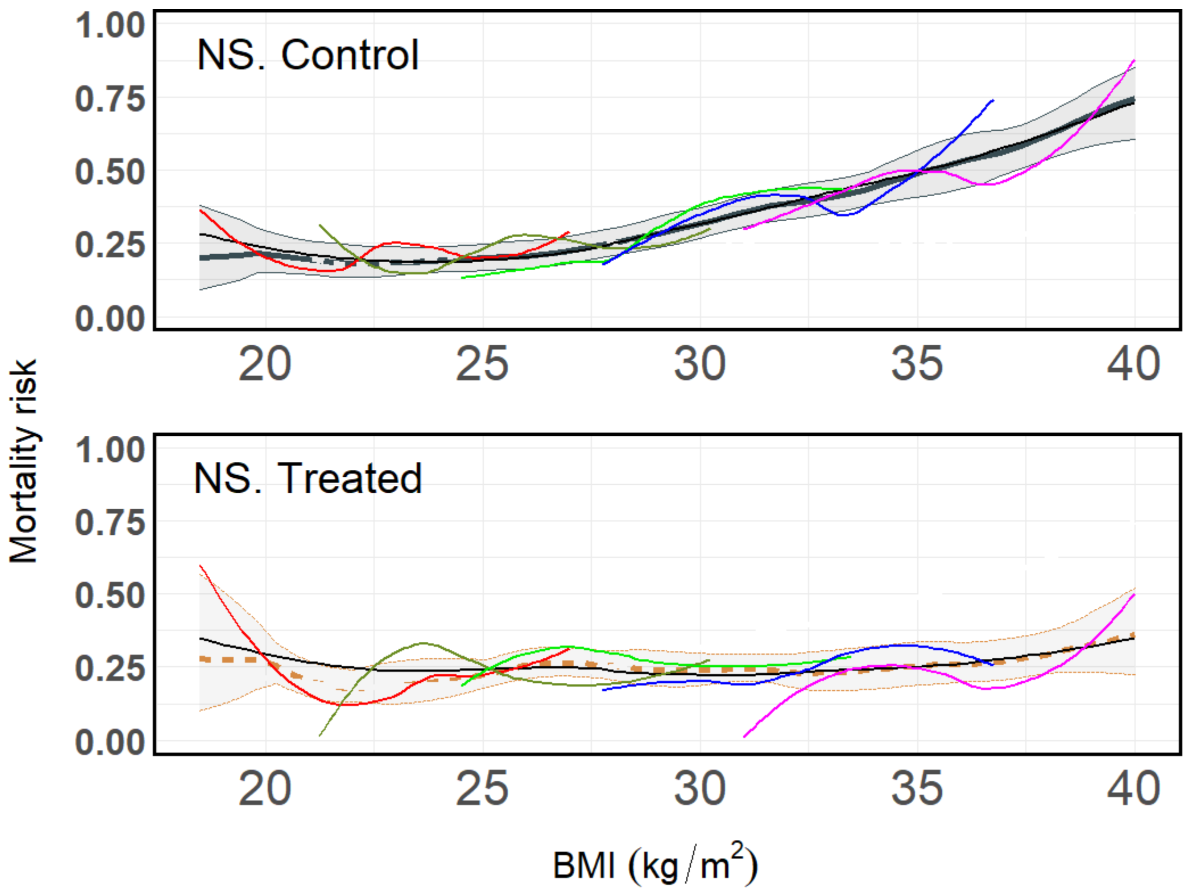

Figure 5.3.F Pooled predicted mortality risk with 95% CI, resulting from pointwise meta-analysis with natural B-splines, including loess plots of the observed mortality data per study

5.4 Pointwise meta-analysis in combination with P-splines

First we show the estimated heterogeneity (tau) across the BMI range (Figure **A**). In combination with the standard errors of the fitted spline curves per study (see section 5.1 in this online appendix), this determines the weights of the studies in the random-effects pointwise meta-analysis.

Next, we present the pooled curves, both on logit and on mortality risk scale (figures **B** and **C**, respectively).

Finally, we show how the individual study predicted spline curves correspond to the pooled predicted curve, both on logit scale (per treatment, figures **D** and **E**), and on the mortality risk scale using loess plots (Figure **F**).

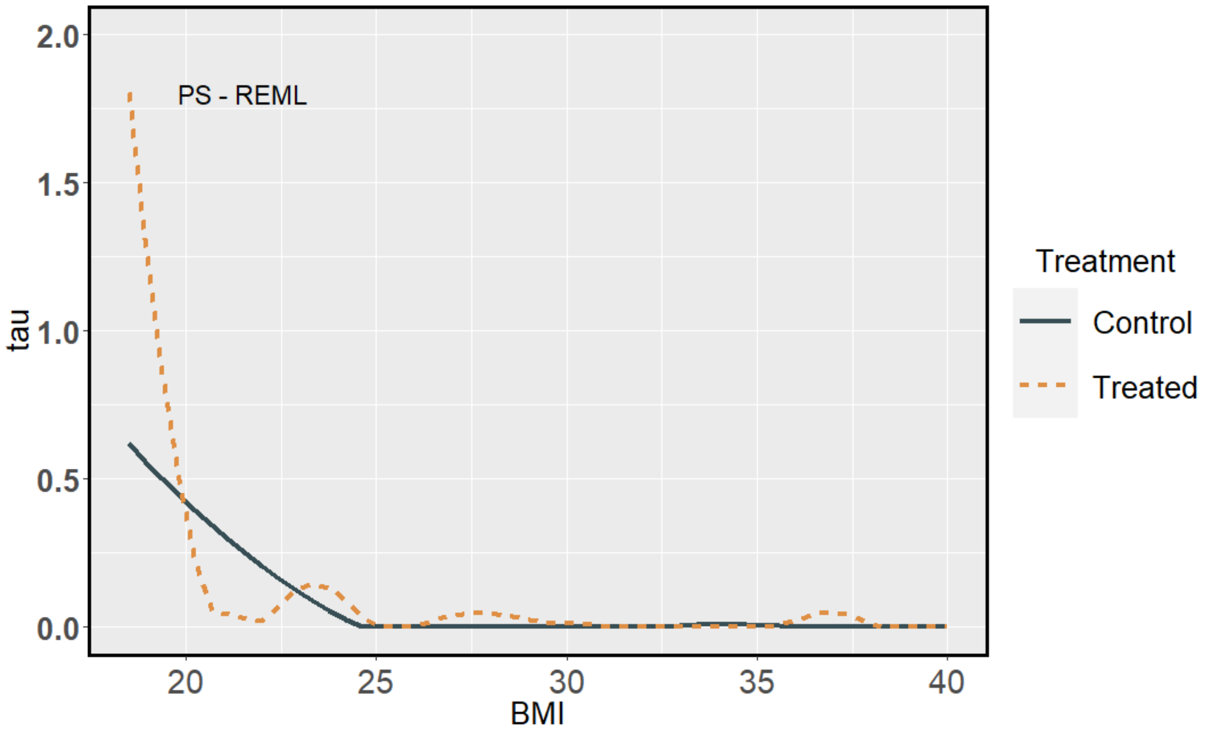

Figure 5.4.A Heterogeneity as estimated by tau (REML), resulting from pointwise meta-analysis with P-splines

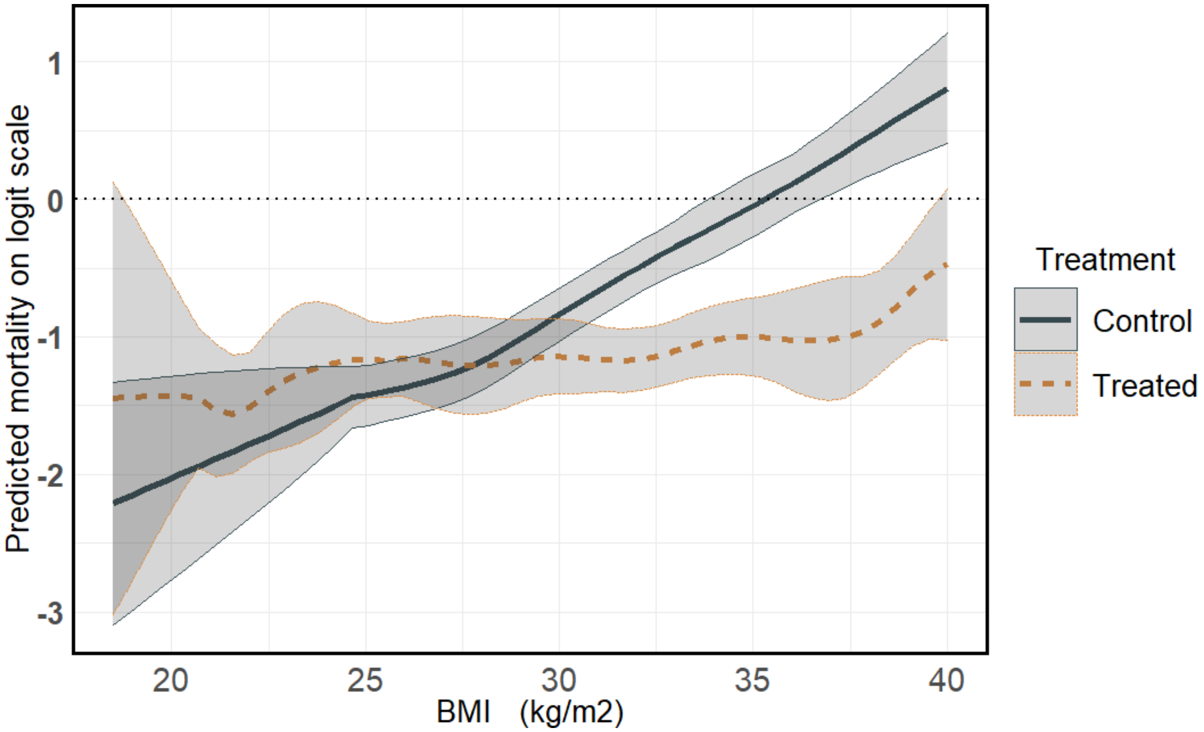

Figure 5.4.B Pooled predicted mortality on logit scale with 95% CI, resulting from pointwise meta-analysis with P-splines

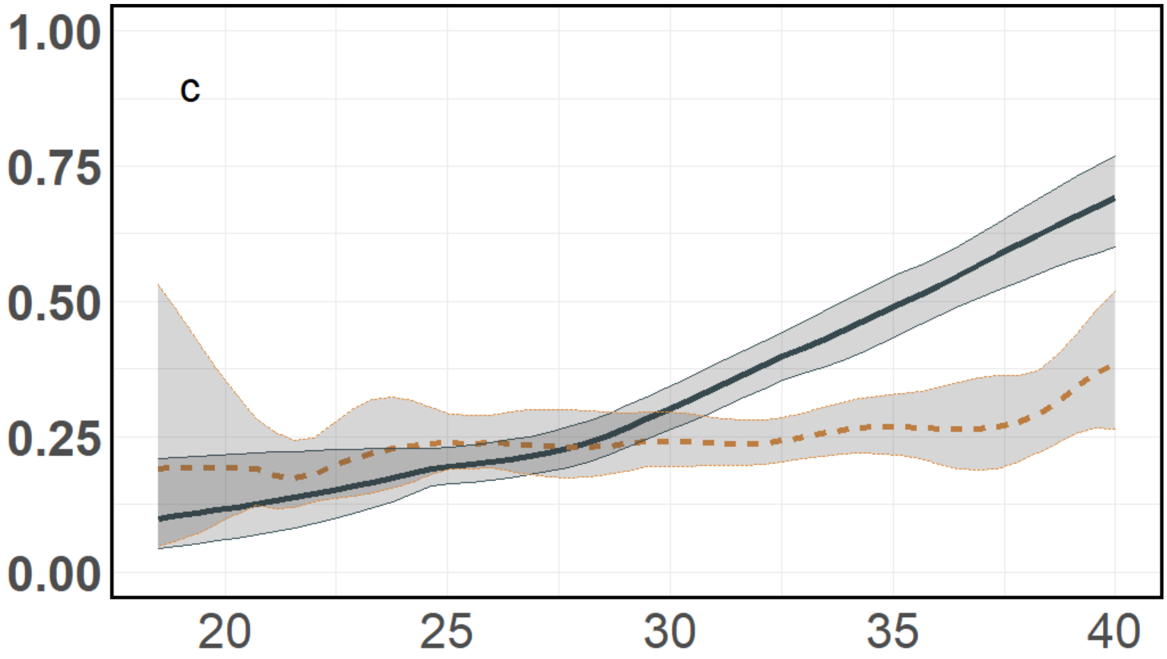

Figure 5.4.C Pooled predicted mortality risk with 95% CI, resulting from pointwise meta-analysis with P-splines
(Figure from main manuscript)

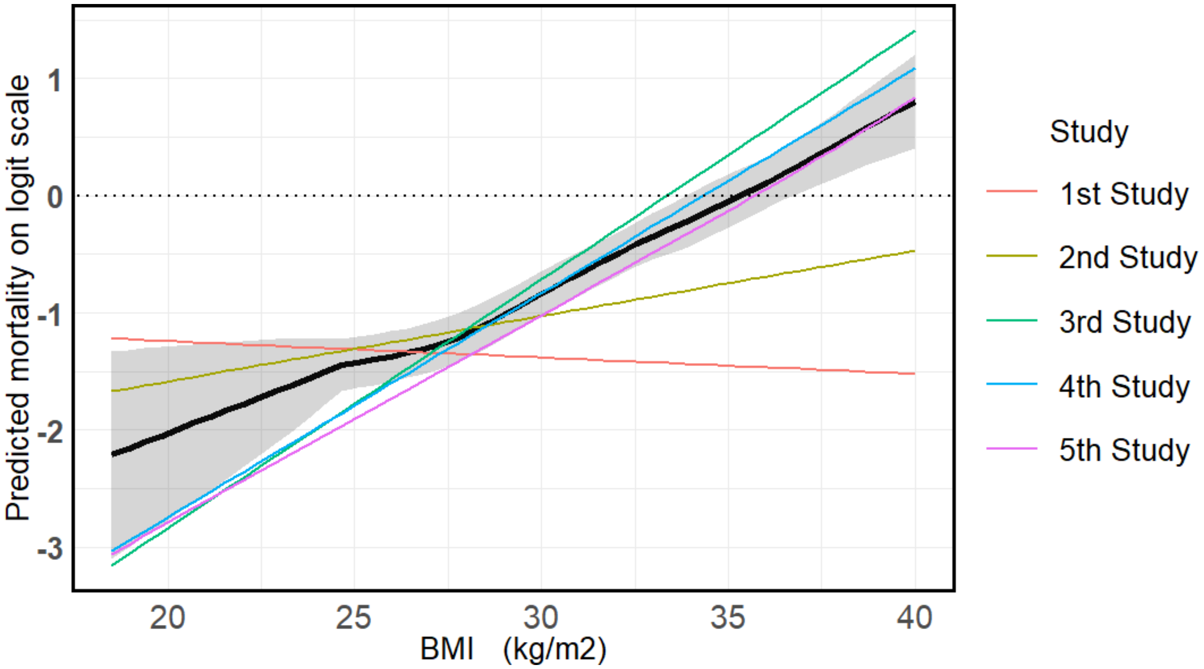

Figure 5.4.D Control group: pooled predicted mortality on logit scale with 95% CI, resulting from pointwise meta-analysis with P-splines, and P-splines per study

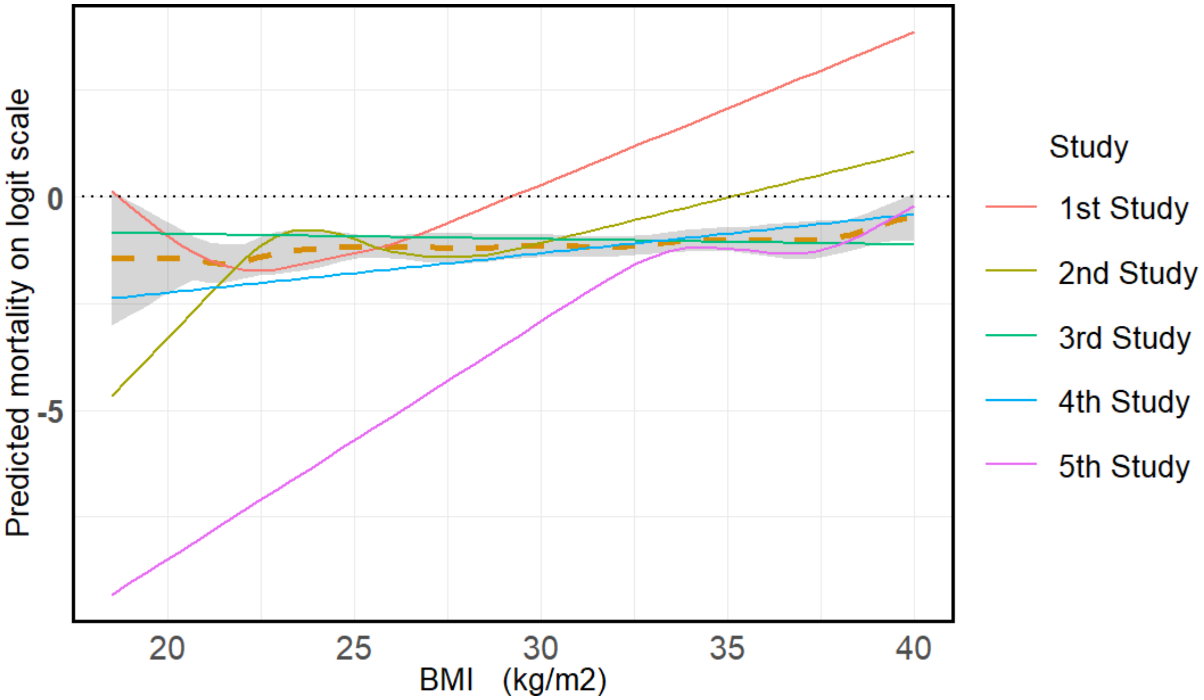

Figure 5.4.E Treated group: pooled predicted mortality on logit scale with 95% CI, resulting from pointwise meta-analysis with P-splines, and P-splines per study

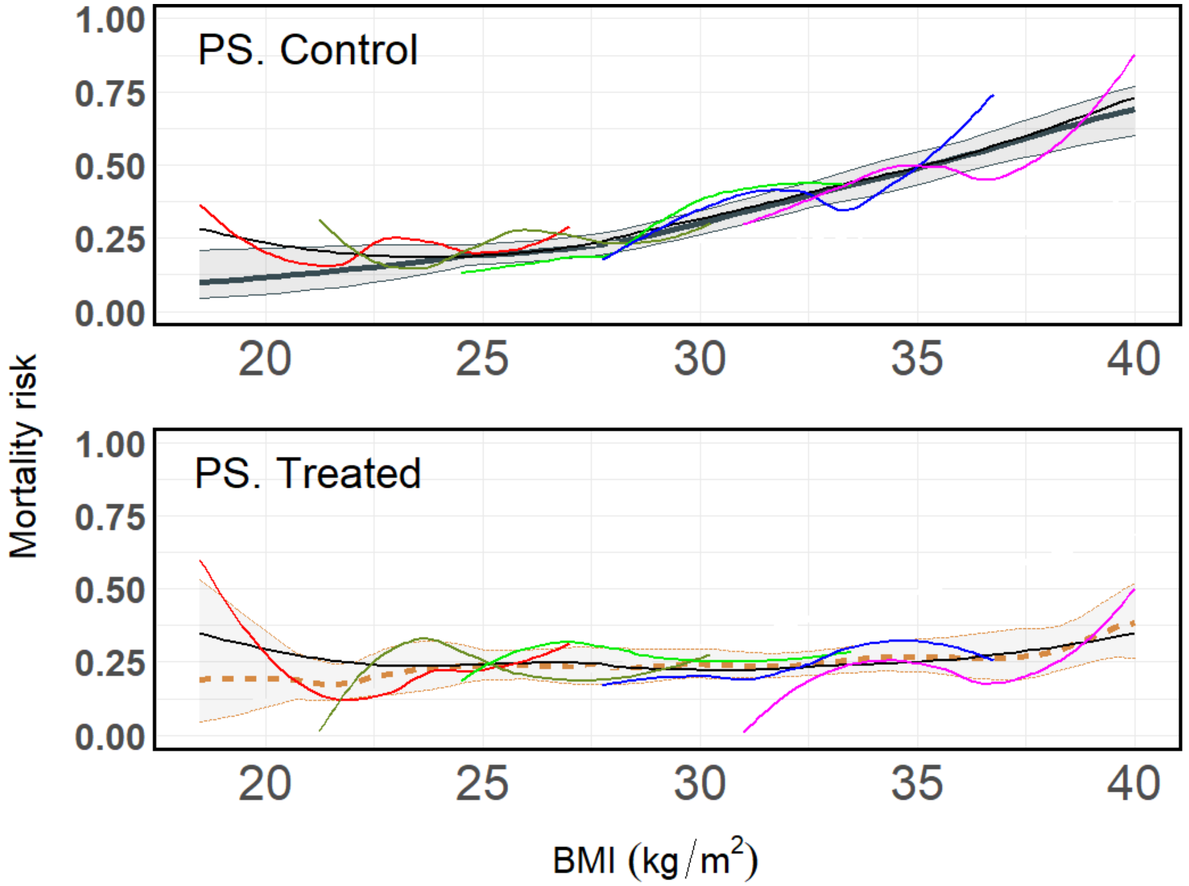

Figure 5.4.F Pooled predicted mortality risk with 95% CI, resulting from pointwise meta-analysis with P-splines, including loess plots of the observed mortality data per study

5.5 Pointwise meta-analysis in combination with smoothing splines

First we show the estimated heterogeneity (tau) across the BMI range (Figure **A**). In combination with the standard errors of the fitted spline curves per study (see section 5.1 in this online appendix), this determines the weights of the studies in the random-effects pointwise meta-analysis.

Next, we present the pooled curves, both on logit and on mortality risk scale (figures **B** and **C**, respectively).

Finally, we show how the individual study predicted spline curves correspond to the pooled predicted curve, both on logit scale (per treatment, figures **D** and **E**), and on the mortality risk scale using loess plots (Figure **F**).

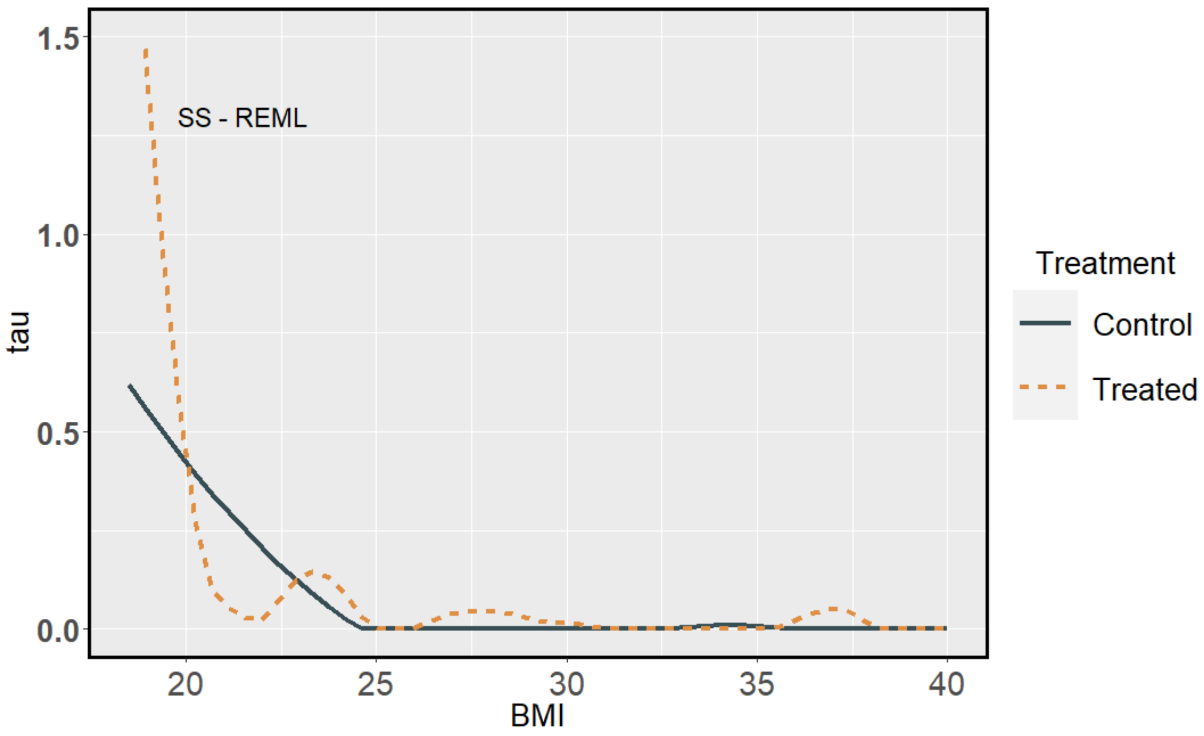

Figure 5.5.A Heterogeneity as estimated by tau (method of DerSimonian Laird, as REML did not converge for some of the BMI values), resulting from pointwise meta-analysis with Smoothing splines

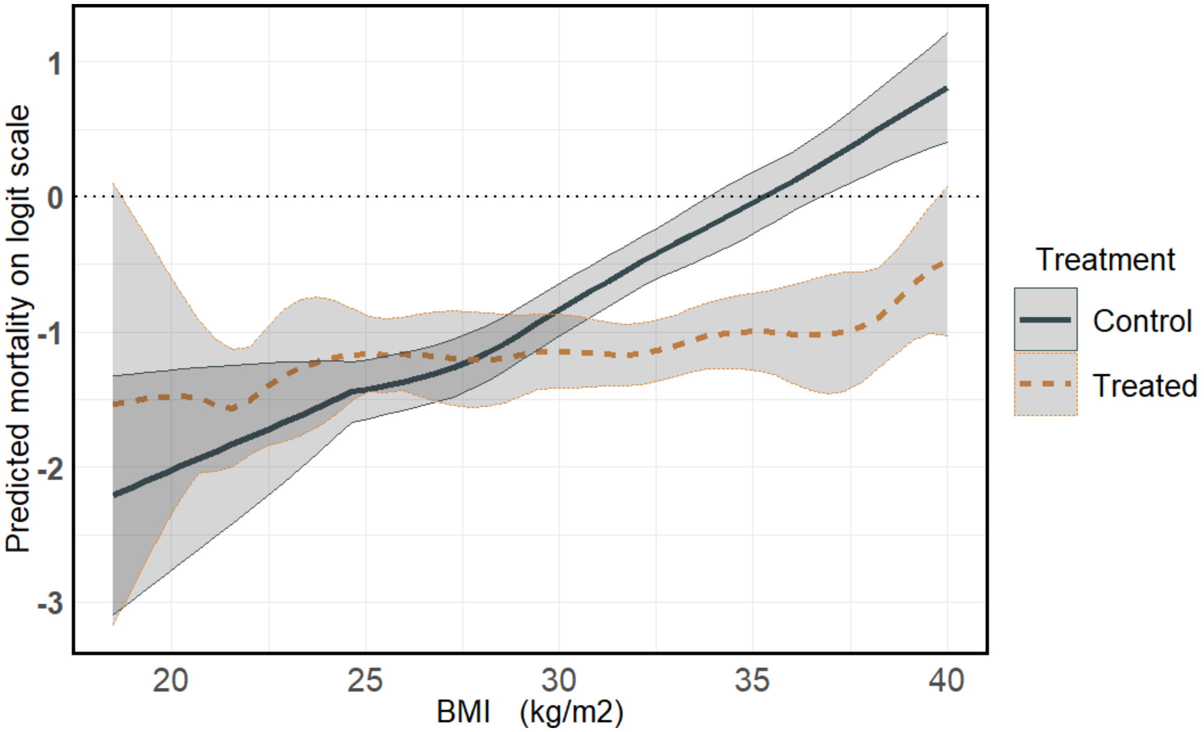

Figure 5.5.B Pooled predicted mortality on logit scale with 95% CI, resulting from pointwise meta-analysis with Smoothing splines

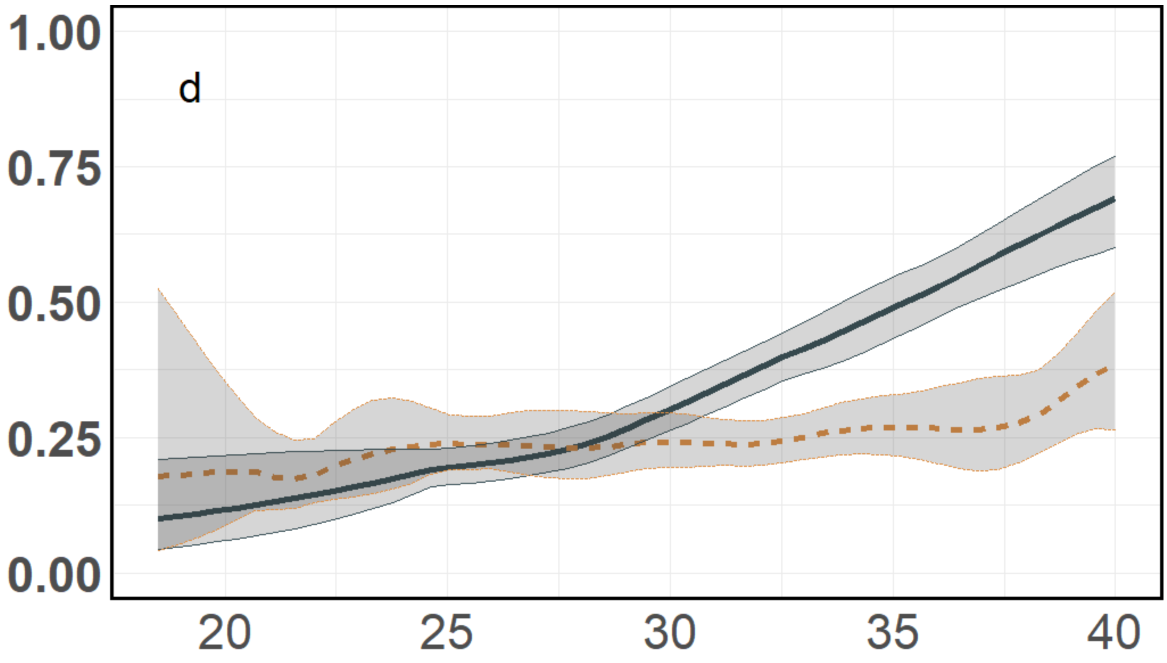

Figure 5.5.C Pooled predicted mortality risk with 95% CI, resulting from pointwise meta-analysis with Smoothing splines
(Figure from main manuscript)

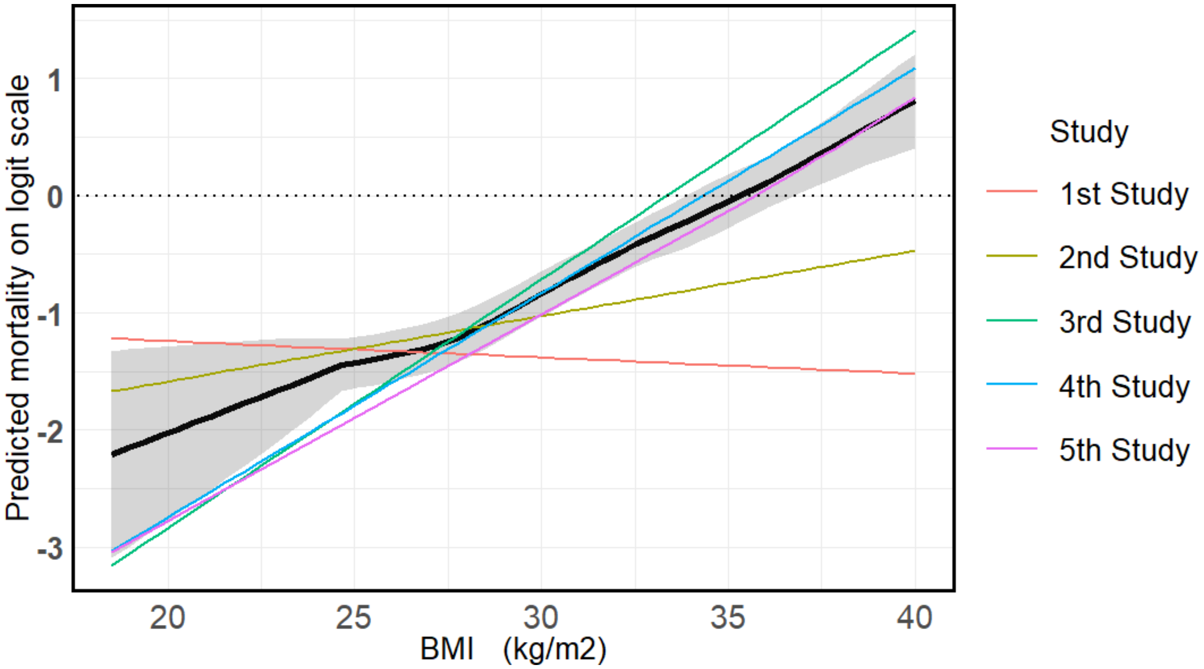

Figure 5.5.D Control group: pooled predicted mortality on logit scale with 95% CI, resulting from pointwise meta-analysis with Smoothing splines, and Smoothing splines per study

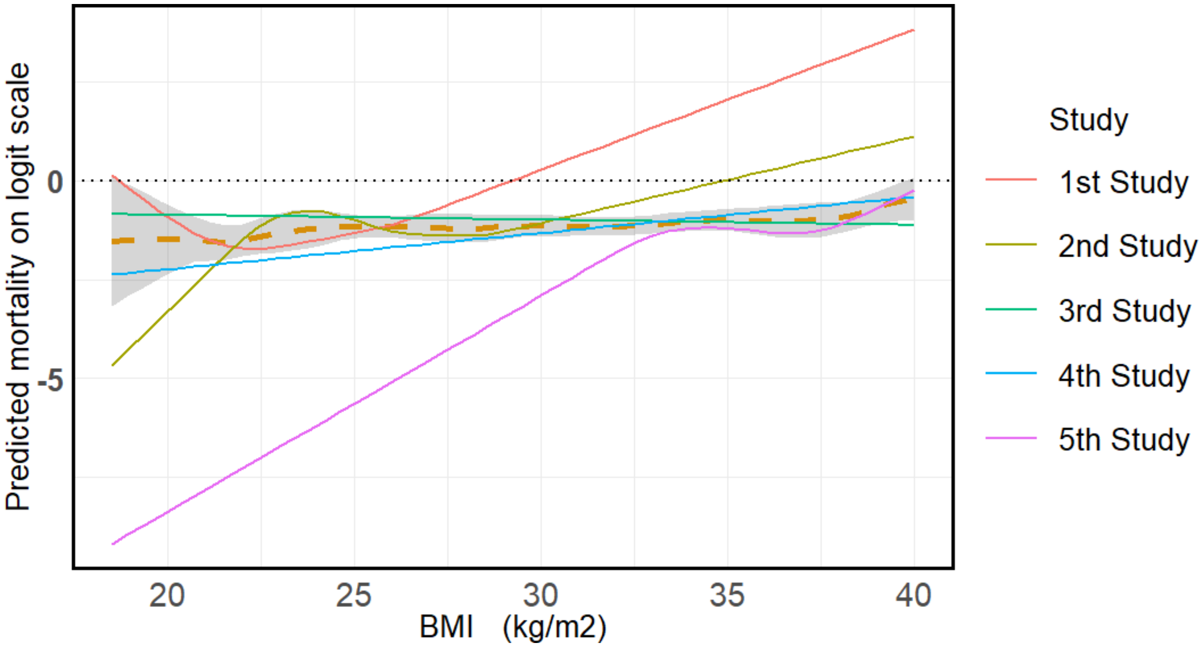

Figure 5.5.E Treated group: pooled predicted mortality on logit scale with 95% CI, resulting from pointwise meta-analysis with Smoothing splines, and Smoothing splines per study

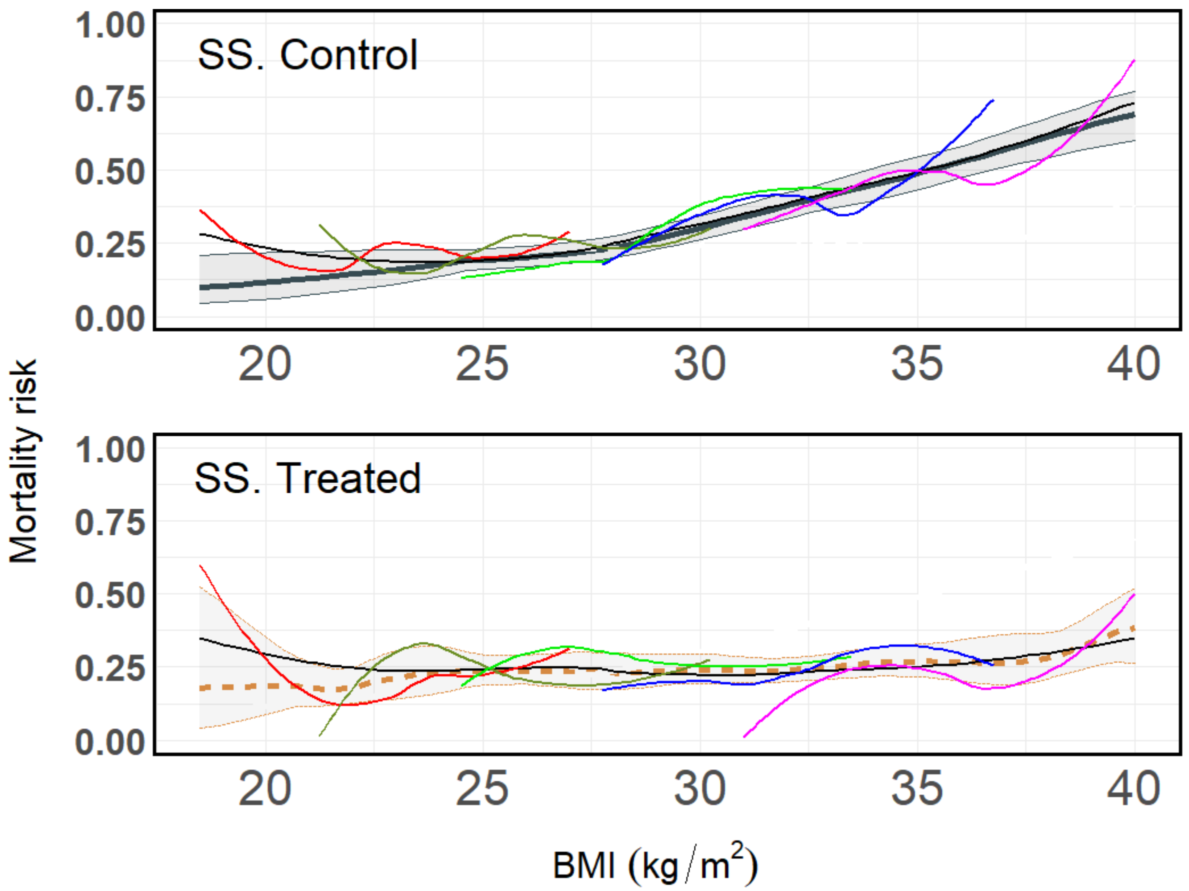

Figure 5.5.F Pooled predicted mortality risk with 95% CI, resulting from pointwise meta-analysis with Smoothing splines, including loess plots of the observed mortality data per study

5.6 Multivariate meta-analysis in combination with natural B-splines

We show the results for the natural B-splines as an example. Because of the different ranges across the studies we first augmented the data with extra observations containing values at the minimum and maximum BMI, with very low weight (see Example R syntax).

Below, we first show the natural B-spline bases for BMI when the inner knots are defines as in the main manuscript (Figure **A**).

Figure **B** shows the pooled predicted mortality per treatment group with 95% CI, resulting from the multivariate meta-analysis, with in addition the predicted natural B-splines per study.

Coefficients and variance-covariance matrices can be found in Tables **A** and **B**. Table 5.6.A shows the coefficients as estimated per study and the pooled coefficients from the multivariate random-effects meta-analysis. The corresponding variance-covariance matrices of Study 1 (as an example on the left side of the BMI range), Study 3 (in the middle), and of the pooled coefficients are presented in Table 5.6.B.

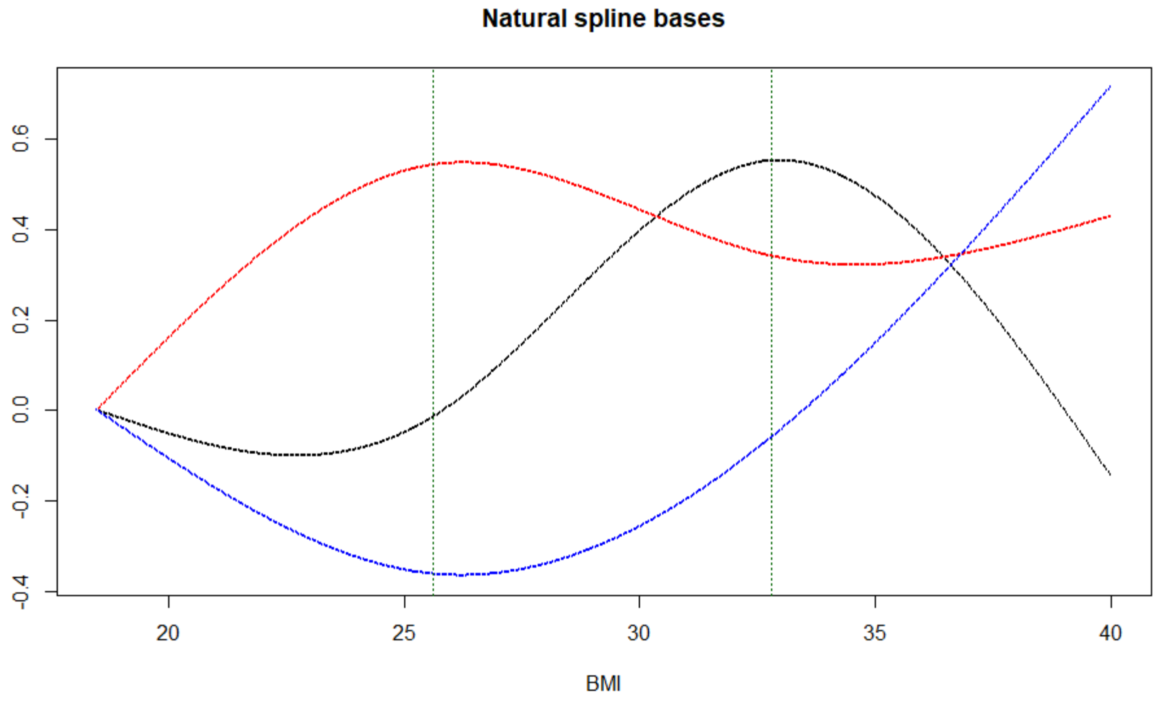

Figure 5.6.A Natural B-splines basis for BMI, with inner knots equal to 25.6 and 32.8.

Black: first basis, Red: second basis; Blue: third basis. Vertical lines: knot positions.
Coefficients in Table 5.6.A are related to these three bases.

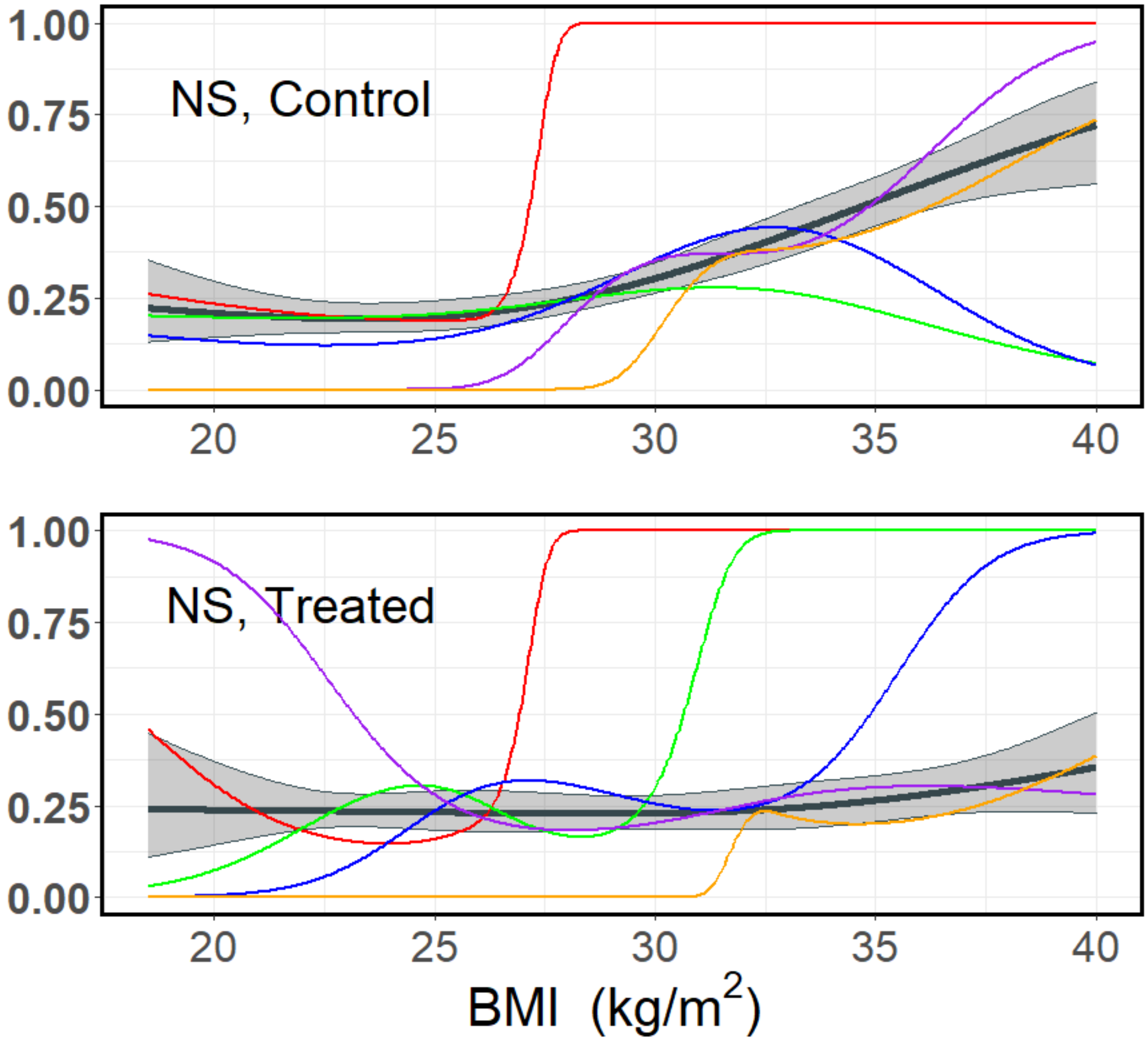

Figure 5.6.B Pooled predicted mortality per treatment group with 95% CI, resulting from multivariate meta-analysis, with the predicted natural B-splines per study.

Colors: Black: pooled (control or treated group),

Red: 1st study, green: 2nd study, blue: 3rd study, purple: 4th study, orange: 5th study.

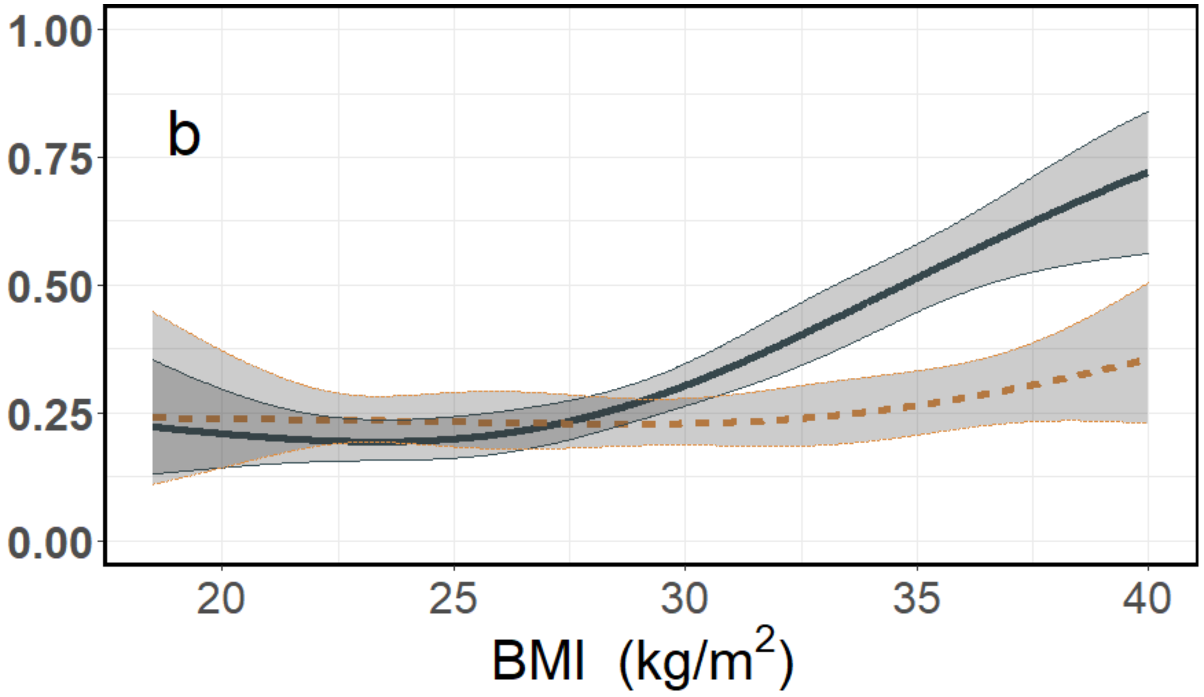

Figure 5.6.C Pooled Mortality as predicted by multivariate meta-analysis in combination with natural B-splines, including 95% CI, based on the pooled coefficients from Tables 5.6.A and 5.6.B.
(Figure from main manuscript)

Table 5.6.A Coefficients estimated with natural B-splines per study, with inner knots equal to 25.6 and 32.8, and in addition the pooled coefficients, derived with multivariate meta-analysis

	**(Intercept)**	**TreatmentTreated**	**ns(BMI)1**	**ns(BMI)2**	**ns(BMI)3**
Study 1	-1.045655	0.8791154	0.4878145	553.8763500	831.907061
Study 2	-1.383312	-2.1066287	0.9313803	-0.5964508	-1.115422
Study 3	-1.759878	-5.2243224	2.8387865	-0.2124582	-0.526464
Study 4	-33.511826	37.1657350	21.8207502	64.3971494	16.690717
Study 5	-137.769938	-1547.9127266	97.7154249	254.9185750	60.905584
**Pooled**	**-1.248579**	**0.1004825**	**1.0216710**	**1.4407810**	**2.419865**

	**TreatmentTreated:ns(BMI)1**	**TreatmentTreated:ns(BMI)2**	**TreatmentTreated:ns(BMI)3**
Study 1	5.046726	41.005113	63.858883
Study 2	-5.499837	47.485328	64.583200
Study 3	-1.122928	16.274807	7.886329
Study 4	-24.374410	-74.097406	-17.818315
Study 5	1110.343409	2855.678263	673.622349
**Pooled**	**-1.128161**	**-1.151091**	**-1.842927**

Table 5.6.B Variance-covariance matrices of the 1st and 3rd study, and of the pooled coefficients:

Variance-covariance matrix of the estimated coefficients of the 1st study:

	coef1	coef2	coef3	coef4	coef5	coef6	coef7	coef8
coef1	0.17	-0.17	0.97	-86.81	-129.82	-0.97	86.81	129.82
coef2	-0.17	0.32	-0.97	86.81	129.82	1.84	-162.92	-243.63
coef3	0.97	-0.97	20.54	-2491.71	-3738.57	-20.54	2491.71	3738.57
coef4	-86.81	86.81	-2491.71	571457.87	857553.40	2491.71	-571457.87	-857553.40
coef5	-129.82	129.82	-3738.57	857553.40	1286882.07	3738.57	-857553.40	-1286882.07
coef6	-0.97	1.84	-20.54	2491.71	3738.57	40.76	-4925.08	-7389.70
coef7	86.81	-162.92	2491.71	-571457.87	-857553.40	-4925.08	1120751.04	1681876.23
coef8	129.82	-243.63	3738.57	-857553.40	-1286882.07	-7389.70	1681876.23	2523942.85

Variance-covariance matrix of the estimated coefficients of the 3rd study:

	coef1	coef2	coef3	coef4	coef5	coef6	coef7	coef8
coef1	39.27	-39.27	-14.34	-99.76	-42.15	14.34	99.76	42.15
coef2	-39.27	71.81	14.34	99.76	42.15	-25.14	-184.78	-80.20
coef3	-14.34	14.34	6.62	33.20	11.21	-6.62	-33.20	-11.21
coef4	-99.76	99.76	33.20	262.24	118.97	-33.20	-262.24	-118.97
coef5	-42.15	42.15	11.21	118.97	61.71	-11.21	-118.97	-61.71
coef6	14.34	-25.14	-6.62	-33.20	-11.21	11.52	58.28	19.62
coef7	99.76	-184.78	-33.20	-262.24	-118.97	58.28	493.45	230.96
coef8	42.15	-80.20	-11.21	-118.97	-61.71	19.62	230.96	123.96

Variance-covariance matrix of the estimated pooled coefficients:

	coef1	coef2	coef3	coef4	coef5	coef6	coef7	coef8
coef1	0.11	-0.06	-0.04	-0.24	-0.04	0.03	0.14	0.03
coef2	-0.06	0.25	0.00	0.17	0.03	-0.05	-0.61	-0.08
coef3	-0.04	0.00	0.11	0.02	-0.01	-0.08	0.07	0.03
coef4	-0.24	0.17	0.02	0.62	0.14	0.00	-0.47	-0.14
coef5	-0.04	0.03	-0.01	0.14	0.13	0.04	-0.14	-0.13
coef6	0.03	-0.05	-0.08	0.00	0.04	0.18	-0.01	-0.05
coef7	0.14	-0.61	0.07	-0.47	-0.14	-0.01	1.65	0.29
coef8	0.03	-0.08	0.03	-0.14	-0.13	-0.05	0.29	0.23

5.7 GAMM in combination with restricted cubic splines

We show the results for the restricted cubic splines as an example.

First we show the restricted cubic spline bases for BMI when the inner knots are defines as in the main manuscript (Figure **A**).

Figure **B** shows the pooled predicted mortality per treatment group with 95% CI, resulting from GAMM, with in addition the by GAMM predicted natural B-splines per study.

Coefficients, standard errors and random effect estimates from this example can be found in Table 5.7.**A** .

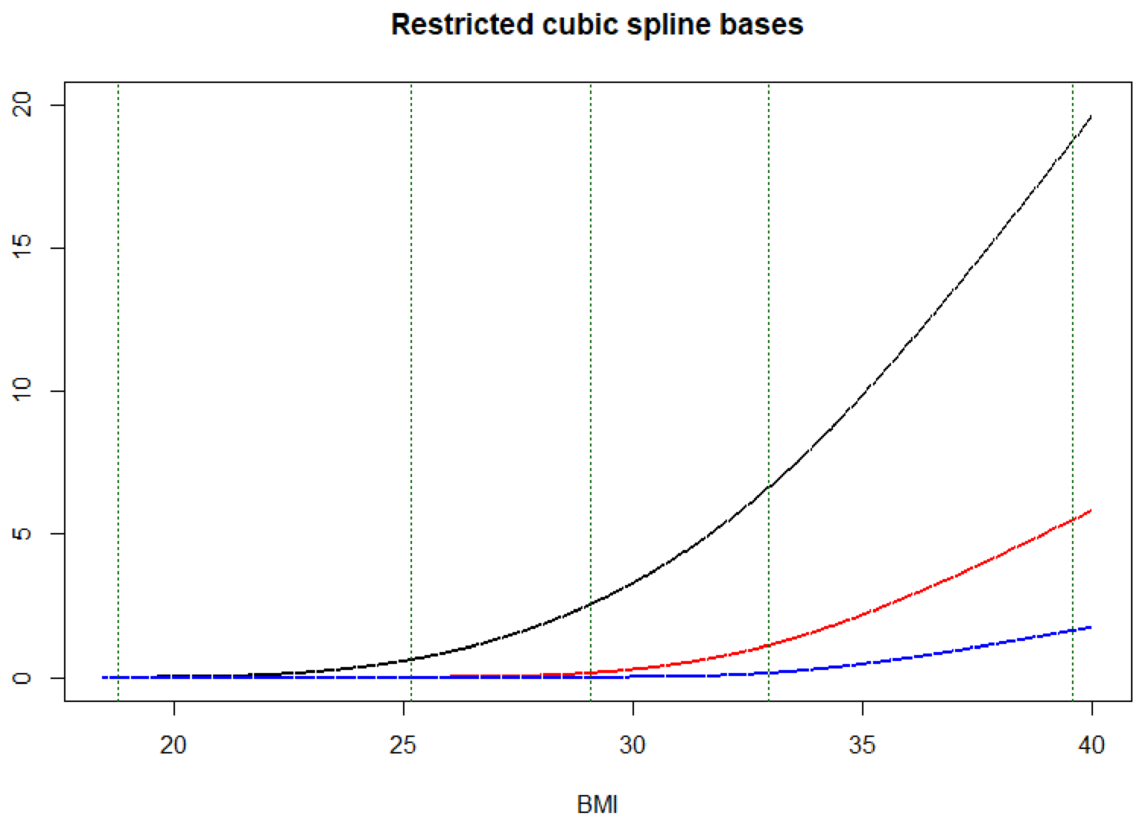

Figure 5.7.A Restricted cubic splines bases (package rms) for BMI, with five knots based on the 0.05, 0.275, 0.5, 0.725, and 0.95 quantiles. Five knots results in 5-1=4 parameters: y=x line, and 3 splines, see above (and intercept). We excluded the y=x linear basis from this plot, as BMI is a separate part of the specified GAMM model.

Black: first basis, Red: second basis; Blue: third basis. Vertical lines: knot positions.
Coefficients in Table 5.7.A are related to these three bases.

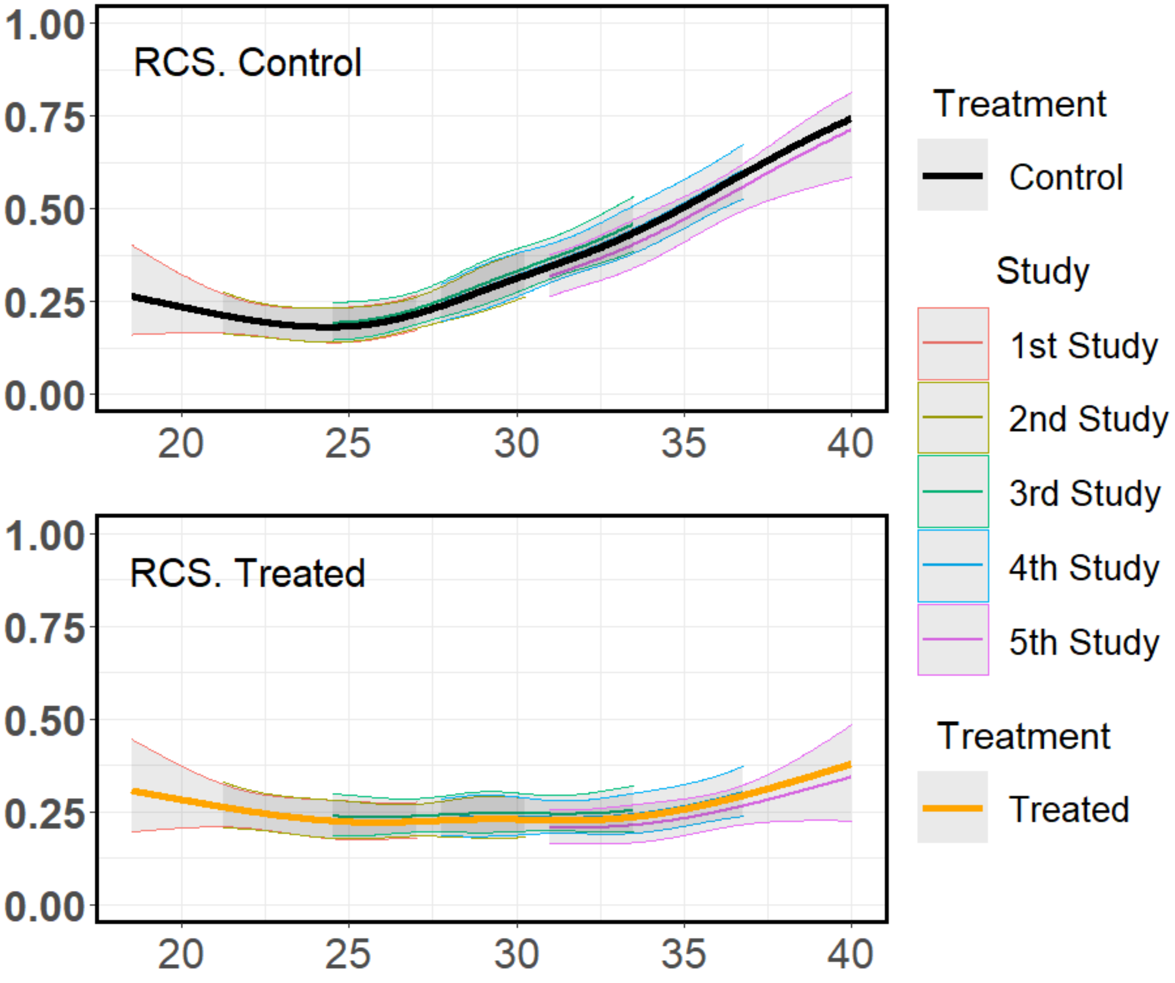

Figure 5.7.B Pooled predicted mortality per treatment group with 95% CI, resulting from GAMM, with the predicted restricted cubic splines per study, also resulting from GAMM. Variation between studies due to heterogeneity as estimated by GAMM.

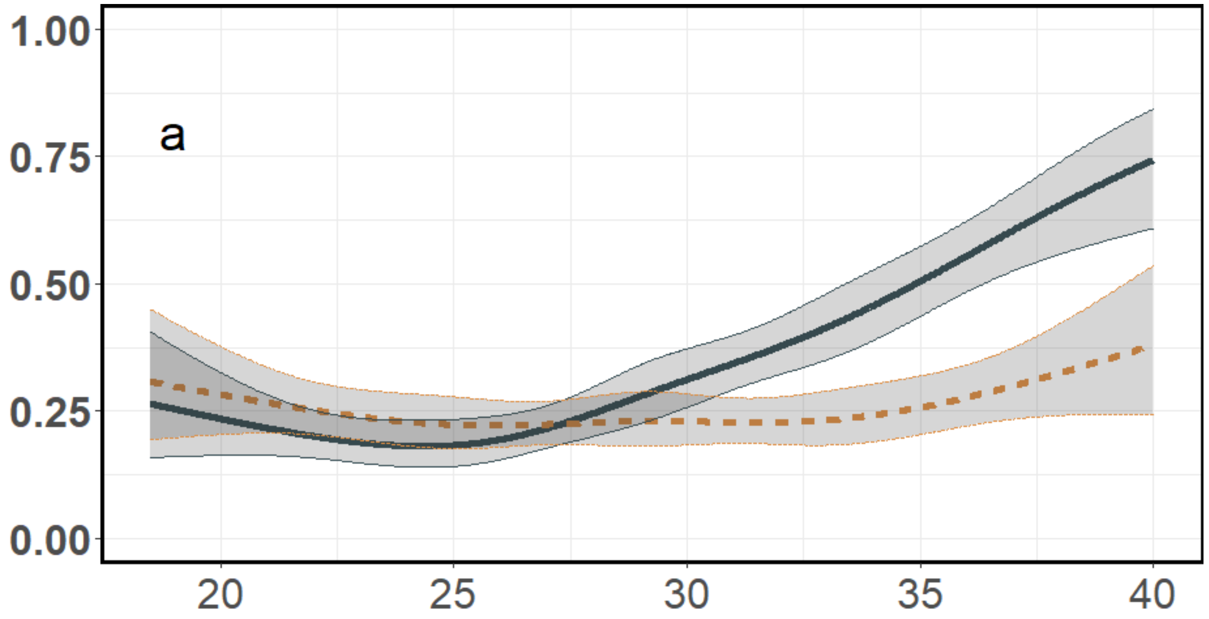

Figure 5.7.C Pooled mortality as predicted by GAMM in combination with restricted cubic splines, including 95% CI, based on the estimated coefficients from Table 5.7.A.
(Figure from main manuscript)

Table 5.7.A Pooled coefficients estimated with GAMM in combination with restricted natural splines, with knots based on the 0.05, 0.275, 0.5, 0.725, and 0.95 quantiles

```
Family: binomial
Link function: logit

Formula:
Y ~ BMI + Treatment + BMI * Treatment + rcspline.eval(BMI, knots = kn) *
    Treatment + s(Study, bs = "re") + s(Study, BMI, bs = "re") +
    s(Study, Treatment, bs = "re")


Parametric coefficients:
                                                Estimate Std. Error z value Pr(>|z|)
(Intercept)                                      0.90041    1.71525   0.525   0.5996
BMI                                             -0.10409    0.07653  -1.360   0.1738
TreatmentTreated                                -0.24430    2.35492  -0.104   0.9174
rcspline.eval(BMI, knots = kn)1                  0.75780    0.41471   1.827   0.0677.
rcspline.eval(BMI, knots = kn)2                 -2.09616    1.63352  -1.283   0.1994
rcspline.eval(BMI, knots = kn)3                  1.93408    2.32492   0.832   0.4055
BMI:TreatmentTreated                             0.02467    0.10520   0.235   0.8146
TreatmentTreated:rcspline.eval(BMI, knots = kn)1 -0.45692    0.57946  -0.789   0.4304
TreatmentTreated:rcspline.eval(BMI, knots = kn)2  1.09264    2.31065   0.473   0.6363
TreatmentTreated:rcspline.eval(BMI, knots = kn)3 -0.46115    3.33331  -0.138   0.8900
---
Signif. codes:  0 '***' 0.001 '**' 0.01 '*' 0.05 '.' 0.1 ' ' 1


Approximate significance of smooth terms:
                          edf Ref.df Chi.sq p-value
s(Study)            0.0009911      4  0.001   0.532
s(Study,BMI)        1.6745010      4  3.254   0.107
s(Study,Treatment)  0.0025053      8  0.001   0.758
```
